# Synthesis of Tryptamine-Thiazolidin-4-one Derivatives and the Combined *In Silico* and *In Vitro* Evaluation of their Biological Activity and Cytotoxicity

**DOI:** 10.1021/acsomega.4c04456

**Published:** 2024-10-23

**Authors:** Seher Aydın, Yavuz Ergün, Salma Ghazy, Asuman Çelebi, Turker Kilic, Timuçin Avşar, Serdar Durdağı

**Affiliations:** † Dokuz Eylul University, The Graduate School of Natural and Applied Sciences, Kaynaklar Campus, Buca, Izmir 35160, Türkiye; ‡ Dokuz Eylul University, Faculty of Sciences, Department of Chemistry, Kaynaklar Campus, Buca, Izmir 35160, Türkiye; § Computational Biology and Molecular Simulations Laboratory, Department of Biophysics, School of Medicine, Bahçeşehir University, Istanbul 34353, Türkiye; ∥ Lab for Innovative Drugs (Lab4IND), Computational Drug Design Center (HİTMER), Bahçeşehir University, İstanbul 34353, Türkiye; ⊥ Department of Medical Biology, School of Medicine, Bahcesehir University, Istanbul 34353, Türkiye; # Department of Neurosurgery, School of Medicine, Bahcesehir University, Istanbul 34353, Türkiye; ∇ Molecular Therapy Lab, Department of Pharmaceutical Chemistry, School of Pharmacy, Bahçeşehir University, Istanbul 34353, Türkiye

## Abstract

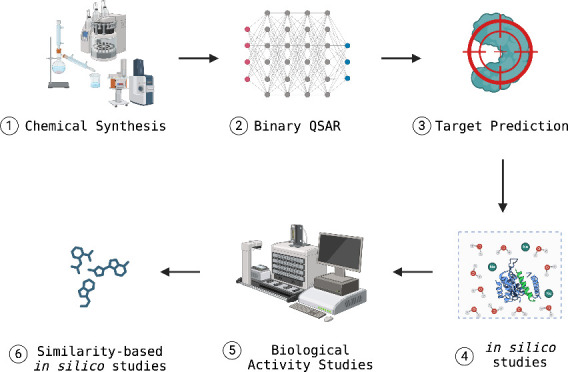

Tryptamine, a monoamine alkaloid with an indole ring structure, is derived from the decarboxylation of the amino acid tryptophan, which is present in fungi, plants, and animals. Tryptamine analogues hold significant therapeutic potential due to their broad pharmacological activities, including roles as neurotransmitters and potential therapeutic agents for various diseases. Structural modifications of tryptamine enhance receptor selectivity and metabolic stability, improving therapeutic efficacy. These modifications are crucial for optimizing pharmacokinetic and pharmacodynamic properties, making the analogues more effective and safer for clinical use. In this study, novel tryptamine-thiazolidin-4-one (**YS1**-**12**) derivatives were synthesized via a one-pot three-component condensation reaction. The synthesized compounds are characterized by different spectroscopy techniques such as FT-IR, ^1^H NMR, ^13^C NMR, and HR-NMS. The synthesized compounds were subjected to binary QSAR disease models for bioactivity prediction and a target prediction model for target analysis. Potential targets were identified, and physics-based molecular simulations were conducted. Additionally, MM/GBSA binding free energy analysis was performed to calculate the average binding free energies of **YS1**-**12** compared to reference molecules. Our computational results indicated promising biological activities for these new compounds. To further investigate these activities, the compounds were tested *in vitro* using two different cancer cell lines: YKG-1 glioblastoma and SH-SY5Y neuroblastoma cells. The results confirmed the potential activities of these novel compounds. Notably, compounds **YS4** and **YS10** exhibited favorable activities compared to the control compounds 5-FU and Temozolomide. **YS4** demonstrated an IC_50_ value of 20 nM against YKG-1 cells, while **YS10** exhibited an IC_50_ value of 0.44 nM against SH-SY5Y cells.

## 1 Introduction

**Figure 1:**
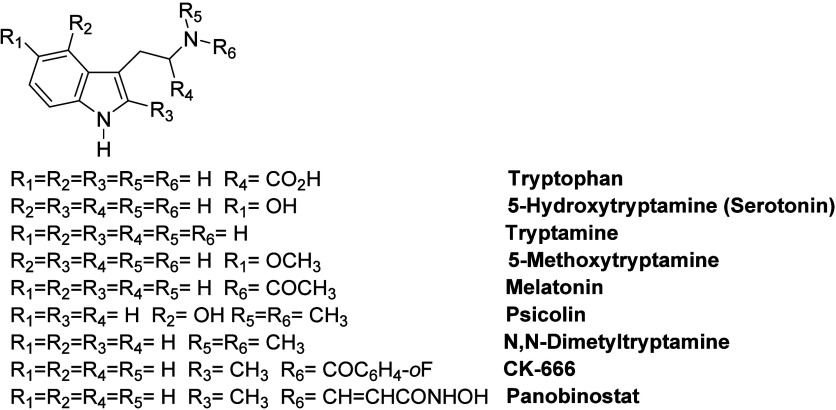
Some important known indol-3-yl ethyl amine derivatives.

Indole scaffold has been found in many of the key synthetic drug molecules and has opened up a reliable way to develop effective targets.^1−3^ The biological inclusion of the indole core pharmacophore recognized in medicinal compounds made it a versatile heterocyclic with a wide range of biological activities.^4^ Among the indole moiety-bearing compounds, many natural or synthetic compounds containing tryptamine (indol-3-yl-ethylamine) units stand out with their various biological activities.^5^ The importance of antidepressant tryptophan in animal and human nutrition and the discovery of plant hormones paved the way for indole chemistry. Serotonin, also known as 5-hydroxytryptamine (5-HT), is a neurotransmitter that is crucial for various physiological functions, including the regulation of pain, appetite, sexual behavior, emotions, sleep, and memory. It is also implicated in several pathological conditions, such as depression, anxiety, schizophrenia, social phobia, panic disorder, and obsessive-compulsive disorder.^6−14^ Melatonin is an important hormone that regulates the sleep cycle and acts as an antioxidant.^9^ Tryptamine, a monoamine alkaloid containing an indole ring structure, is derived by the decarboxylation of amino acid tryptophan.^15^ Psychoactive tryptamines such as psilocin and *N, N*-dimethyltryptamine are naturally found in toads, plants, and mushrooms.^15−20^ However, many synthetic tryptamine derivatives have been synthesized and used as novel psychoactive substances and serotonergic hallucinogens. Tryptamine analogs exhibit significant therapeutic potential due to their wide range of pharmacological activities, functioning as neurotransmitters and potential therapeutic agents for various diseases. Structural modifications to tryptamine enhance receptor selectivity and metabolic stability, thereby improving therapeutic efficacy. These modifications are essential for optimizing pharmacokinetic and pharmacodynamic properties, making the analogs more effective and safer for clinical use. The psychoactive effects of tryptamine-based hallucinogens are thought to be mediated mainly by the 5-HT2A receptor. Still, they may also be modulated by interactions with other targets, including other 5-HT receptors, monoamine transporters, and trace amine-associated receptors.^16^ Additionally, arp 2/3 inhibitor CK-666 and antileukemic drug panobinostat have indol-3-yl ethyl amine unit.^21,22^ Some important indol-3-yl ethyl amine derivatives are represented in Figure 1.

**Figure 2:**
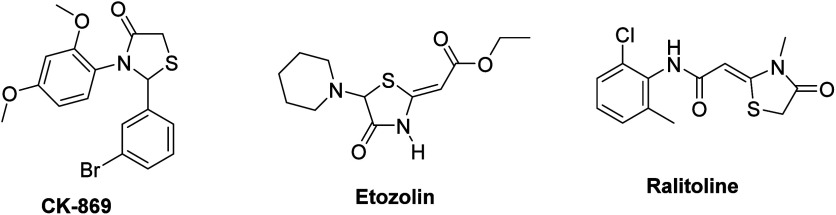
Some important known thiazolidin-4-one derivatives.

Compounds containing the thiazolidinone scaffold, which is another crucial part of this study, stand out with their structures and several biological activities such as antibacterial, antiviral/HIV, anticancer, anticonvulsant, antihistaminic, anti-inflammatory and cardiovascular agents.^23−30^ Additionally, the successful introduction of CK-869 as an arp 2/3 inhibitor,^31^ etozoline as an antihypertensive,^32^ and ralitoline as a potent anticonvulsant proved the potential of thiazolidinone moiety.^33^ Some important thiazolidin-4-one derivatives are depicted in Figure 2.

**Scheme 1:**
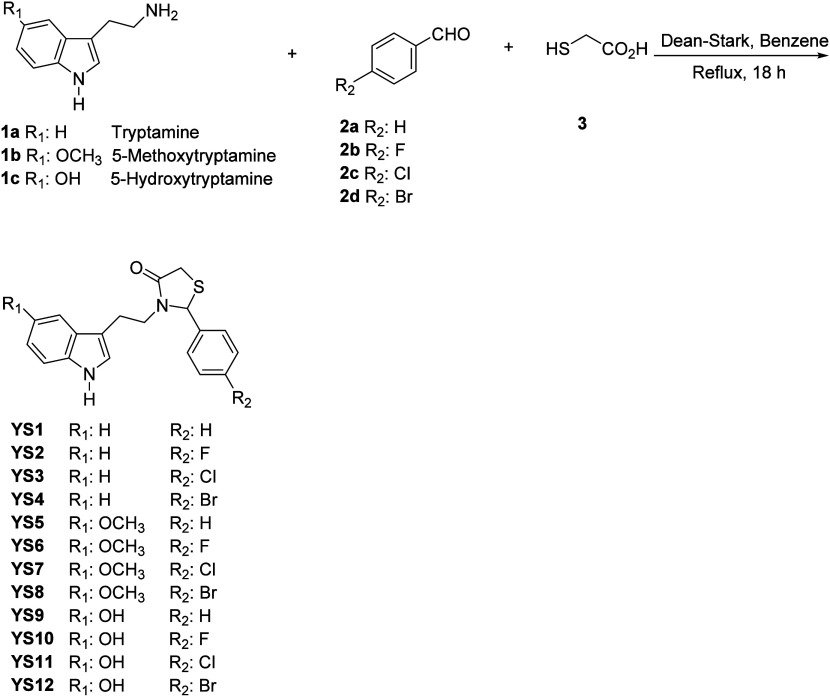
Synthesis of Tryptamine-Thiazolidin-4-one Derivatives

In this study, we have synthesized novel tryptamine-thiazolidin-4-one derivatives for the first time in the literature. (Scheme 1) The main synthetic routes to tryptamine-thiazolidin-4-ones (**YS1–12**) involve three components that are tryptamine derivatives (**1a**–**c**), benzaldehyde derivatives (**2a**–**d**), and mercaptoacetic acid (**3**) via a one-pot three-component condensation reaction. The reactions begin with the formation of imine (the nitrogen of amine attacks the carbonyl of aldehyde), which undergoes attack by a generated sulfur nucleophile, followed by intramolecular cyclization on the elimination of water.^34^

A thorough binary QSAR analysis for therapeutic activity prediction pinpointed mainly schizophrenia, heart disease, and hypertension as potential target diseases. The Adenosine-1 receptor (A1AR), a member of the G protein-coupled receptor (GPCR) family, was predicted to be one of the potential target proteins for these synthesized compounds. A1AR mediates most of the physiological effects of extracellular adenosine.^35^ It is a primary target of heart disease and hypertension.^36^ Both the inactive (PDB ID: 5N2S)^37^ and active (PDB ID: 7LD4)^38^ states of A1AR have been integrated into this study to observe the effect of the synthesized ligands on two different states of the protein. 5N2S is the crystal structure of the stabilized A1AR receptor in complex with PSB36 (PubChem CID: 92460631). The crystal structure was obtained using X-ray diffraction at 3.30 Å resolution. 7LD4 coded PDB is the cryo-EM structure of the human adenosine A1 receptor-G(i)2-protein complex bound to its endogenous agonist adenosine. The structure was produced via electron microscopy at 3.30 Å resolution.

In addition, phosphodiesterase 10A (PDE10A) (PDB ID: 8DI4)^39^ was also predicted as a potential target for these synthesized ligands and it is included in the current study. PDE10A is an enzyme found mainly in the striatal medium spiny neurons (MSN), regulating their excitability via deactivation of the transmitters cyclic adenosine monophosphate (cAMP) and cyclic guanosine monophosphate (cGMP) working in a postsynaptic basis on dopamine signaling. Well-recognized antipsychotic drugs primarily help cure schizophrenia by inhibiting D_2_ dopamine receptors in the striatum, effectively downregulating positive symptoms.^40^ 8DI4 is the crystal structure of PDE10A studied by X-ray diffraction at 2.20 Å resolution.

Thus, in the current study, 12 tryptamine-thiazolidin-4-one derivatives (**YS1–12**) were synthesized, and their biological activities were investigated using state-of-the-art physics-based *in silico* techniques and *in vitro* biological activity studies.

## 2 Results and Discussion

In this study, a series of tryptamine-thiazolidin-4-one derivatives (**YS1–12**) were successfully synthesized using a one-pot, three-component condensation reaction, with yields ranging between 40 and 45%. The formation of the thiazolidinone ring was verified by the detection of a carbonyl stretch in the FT-IR spectrum, appearing at 1650–1670 cm^–1^. In the ^1^H NMR spectra, the methylene group within the thiazolidin-4-one ring exhibited diastereotopic protons, with one proton resonating around 3.69–3.70 ppm and the other near 3.79–3.80 ppm. Additionally, a distinct signal at approximately 5.21–5.77 ppm in the ^1^H NMR spectrum was attributed to the hydrogen atom bonded to the carbon atom positioned between the nitrogen and sulfur atoms in the thiazolidin-4-one ring. These spectral findings align with previously reported data for thiazolidin-4-one rings. Furthermore, the ^1^H NMR spectra of the synthesized compounds revealed that both the methylene hydrogens in the thiazolidinone ring and the methylene hydrogens linked to the nitrogen in the tryptamine segment were diastereotopic, indicating the successful formation of the tryptamine-thiazolidin-4-one structure.^41,42^ Results of FT-IR, ^1^H NMR, ^13^C NMR and HRMS spectra of synthesized tryptamine-thiazolidin-4-one derivatives (**YS1–12**) were displayed in the Supplementary figures S1–S48.

Binary QSAR models (i.e., MetaDrug/MetaCore) from Clarivate Analytics (https://portal.genego.com/) were then used to predict the therapeutic activity values of these synthesized compounds. Results showed that, particularly, compounds represented activity against depression, HIV, heart failure, obesity, migraine, asthma, arthritis, and hypertension (Supplementary table S5). Furthermore, the SwissTargetPrediction tool (http://www.swisstargetprediction.ch/) was used for the prediction of crucial target proteins for these ligands, and A1AR and PDE10A targets were identified (Supplementary tables S6–S17). Thus, 7LD4^37^ and 5N2S^38^ were used as active and inactive forms of A1AR, respectively, to measure the effects of synthesized ligands against heart diseases and hypertension. PDE10A (PDB ID: 8DI4)^39^ was also incorporated to gauge the effects of ligands against heart failure.

**Figure 3:**
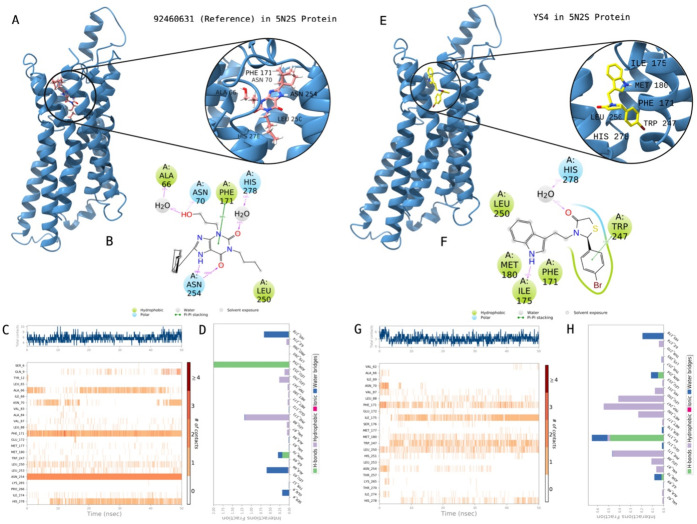
Interactions of compound **YS4** with the critical residues on the A1AR protein (PDB ID: 5N2S) in comparison with the reference molecule 92469631. Surface representation of 92469631on the A1AR protein (a). Ligand interaction diagram of 92469631 with the critical residues (b). Time-dependent protein-92469631 contact panel throughout 50 ns MD simulations. The upper panel indicates the total contacts, while the lower panel highlights the formed and broken interactions (c). Interaction fractions and characterization of interactions of binding pocket residues of A1AR protein with 92469631 throughout the MD simulation (d). Surface representation of **YS4** on the A1AR protein (e). Ligand interaction diagram of **YS4** with the critical residues (f). Time-dependent protein-**YS4** contact panel throughout 50 ns MD simulations. The upper panel indicates the total contacts, while the lower panel indicates the constructed and broken interactions (g). Interaction fractions and characterization of interactions of binding pocket residues of A1AR protein with **YS4** throughout MD simulations (h). This figure is created with BioRender and ChimeraX.

The docking scores and average MM/GBSA binding free energy values of synthesized compounds at the inactive state of the A1AR are displayed in Table 1. Figure 3 shows the 2D protein–ligand interactions and contacts of **YS4** which is the most active compound through biological activity studies. Crucial contacts were formed by Phe171, Ile175, Met180, Trp247, Leu250 and His278 with **YS4**. The same protocol was also conducted for the known active compound (i.e., reference PubChem CID: 92460631). Corresponding interactions were constructed by Ala66, Asn70, Phe171, Leu250, Asn254, and His278 with the reference ligand.

**Table 1:** Molecular Docking and Average MM/GBSA Scores of Studied Compounds at the Inactive State of A1AR Target Protein (PDB ID: 5N2S)

**ligand**	**docking score** (kcal/mol)	**average MM/GBSA** (kcal/mol)
PubChem CID: 92460631(Reference)	–12.03	–83.29 ± 4.16
**YS10**	–8.46	–75.80 ± 6.18
**YS11**	–8.23	–69.00 ± 4.17
**YS7**	–7.76	–67.94 ± 3.87
**YS2**	–8.01	–65.22 ± 4.09
**YS4**	–7.45	–64.07 ± 4.07
**YS3**	–7.33	–62.06 ± 4.12
**YS12**	–8.25	–59.25 ± 5.96
**YS5**	–7.13	–59.17 ± 4.23
**YS1**	–6.87	–58.23 ± 3.80
**YS8**	–7.43	–57.73 ± 5.28
**YS9**	–7.16	–56.48 ± 4.42
**YS6**	–7.10	–55.70 ± 5.29

**Figure 4:**
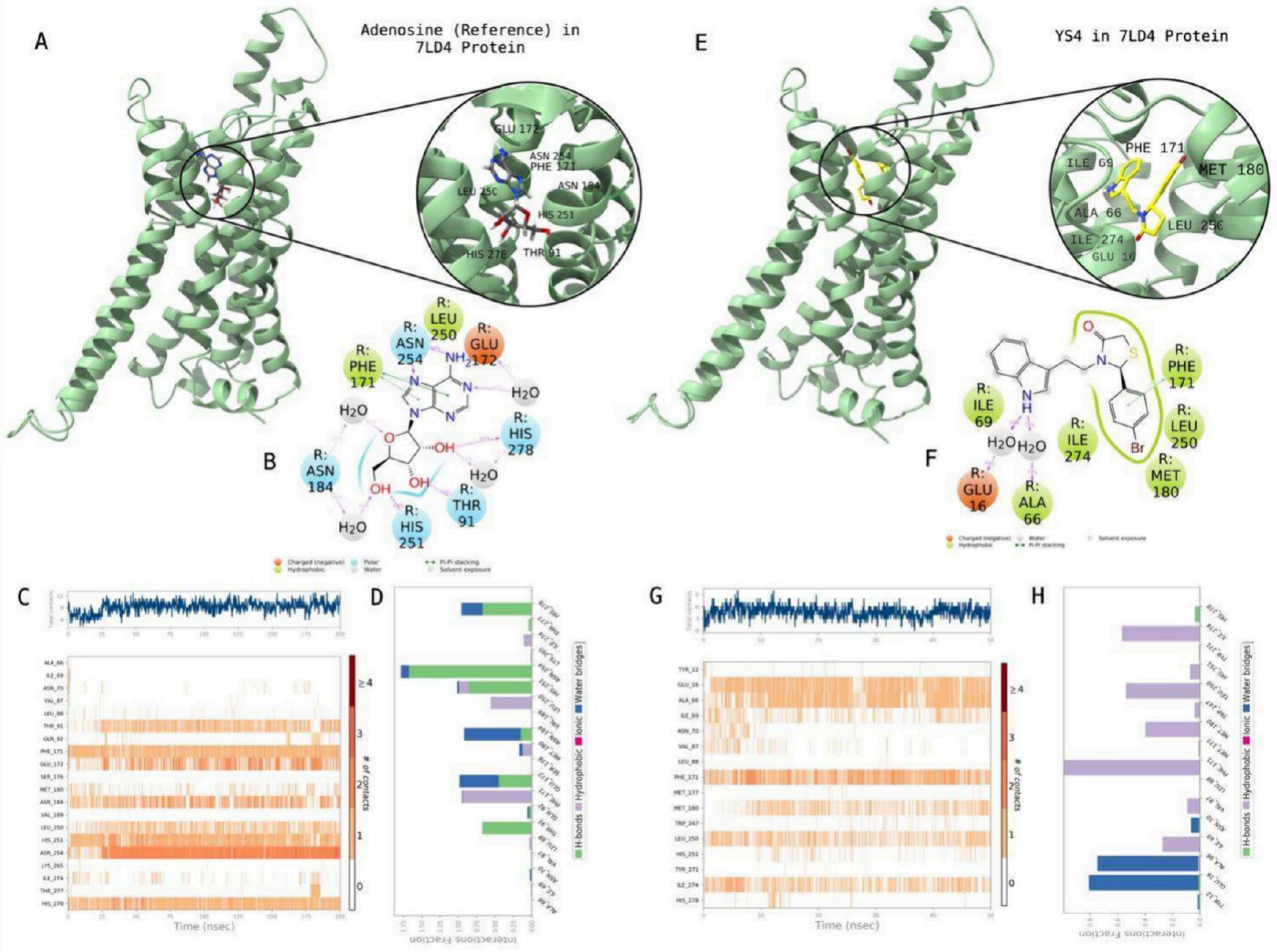
Interactions of compound **YS4** with the critical residues on the A1AR protein (PDB ID: 7LD4) in comparison with the reference molecule Adenosine. Surface representation of Adenosine on the A1AR protein (a). Ligand interaction diagram of Adenosine with the critical residues (b). Time-dependent protein-Adenosine contact panel throughout 50 ns MD simulations. The upper panel indicates the total contacts, while the lower panel highlights the formed and broken interactions (c). Interaction fractions and characterization of interactions of binding pocket residues of A1AR protein with Adenosine throughout the MD simulations (d). Surface representation of **YS4** on the A1AR protein (e). Ligand interaction diagram of **YS4** with the critical residues (f). Time-dependent protein-**YS4** contact panel throughout 50 ns MD simulations. The upper panel indicates the total contacts, while the lower panel indicates the formed and broken interactions (g). Interaction fractions and characterization of interactions of binding pocket residues of A1AR protein with **YS4** throughout MD simulations (h). This figure is created with BioRender and ChimeraX.

Table 2 shows molecular docking and average MM/GBSA scores of synthesized compounds at the active state of the A1AR. Figure 4 highlights the 2D protein–ligand contacts and interactions of **YS4** in comparison with the reference molecule Adenosine ligand. While Thr91, Phe171, Glu172, Asn184, Leu250, His251, Asn254, and His278 were found crucial residues for the contacts with the reference molecule, the corresponding amino acids were listed as Glu16, Ala66, Ile69, Phe171, Met180, Leu250, and Ile274 for constructing the stable contacts with **YS4**.

**Table 2:** Docking and Average MM/GBSA Scores of Studied Compounds at the Active State of A1AR Target Protein (PDB ID: 7LD4)

**ligand**	**docking score** (kcal/mol)	**average MM/GBSA** (kcal/mol)
**YS7**	–6.40	–69.70 ± 4.84
**YS6**	–6.52	–67.98 ± 5.03
**YS12**	–7.44	–65.42 ± 5.25
**YS11**	–7.97	–64.03 ± 0.38
**YS10**	–5.99	–62.76 ± 5.21
Adenosine (Reference)	–9.39	–62.26 ± 5.36
**YS3**	–7.16	–61.20 ± 5.53
**YS2**	–7.22	–60.02 ± 4.68
**YS5**	–7.51	–59.96 ± 5.39
**YS9**	–7.74	–59.68 ± 4.30
**YS8**	–5.16	–59.42 ± 5.30
**YS4**	–6.64	–59.17 ± 4.83
**YS1**	–7.38	–57.64 ± 3.99

**Figure 5:**
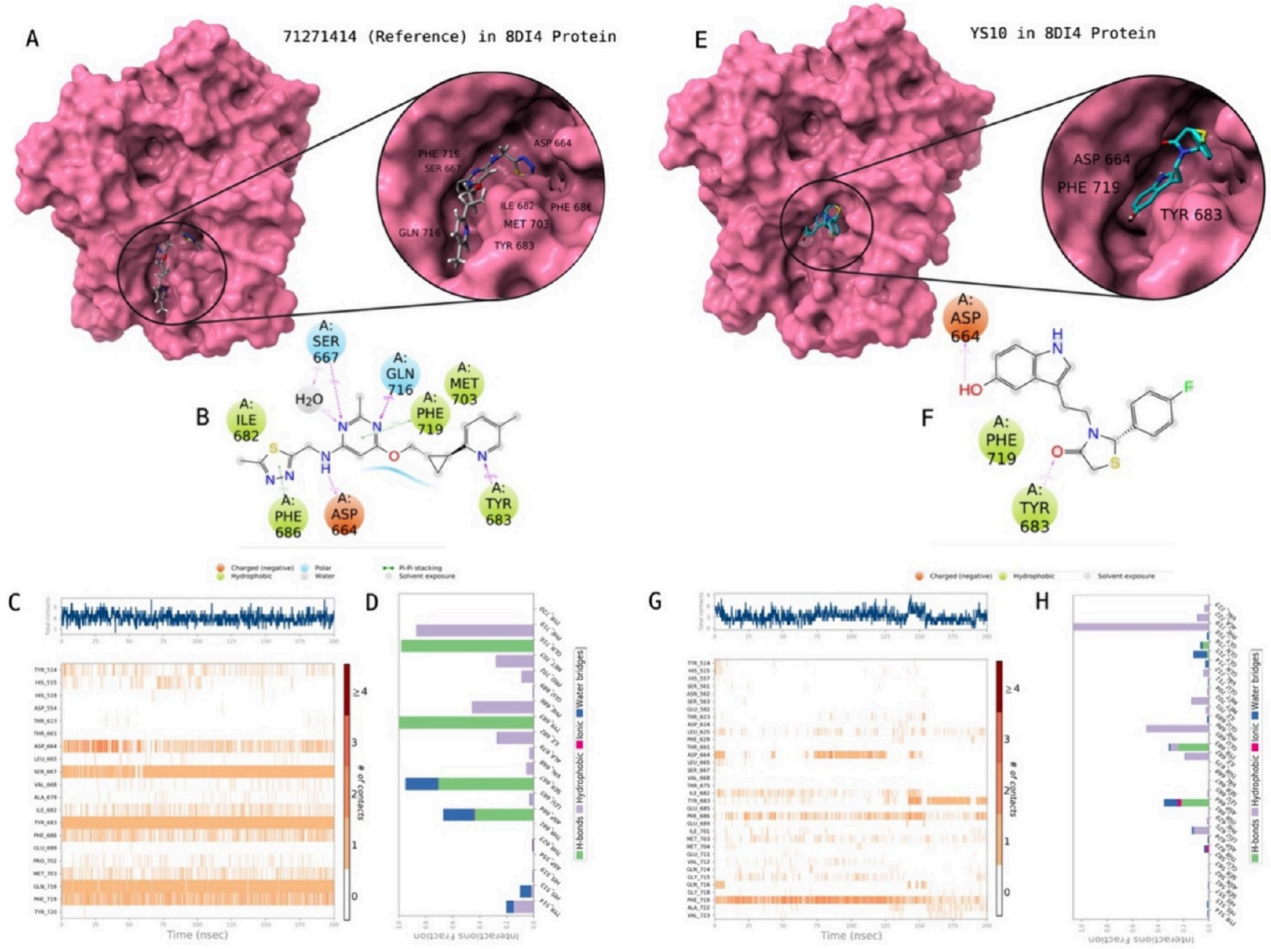
Interactions of compound **YS10** with the critical residues on the PDE10A protein (PDB ID: 8DI4) in comparison with the reference molecule 71271414. Surface representation of 71271414 on the PDE10A protein (a). Ligand interaction diagram of 71271414 with the critical residues (b). Time-dependent protein-71271414 contact panel throughout 200 ns MD simulations. The upper panel indicates the total contacts, while the lower panel highlights the formed and broken interactions (c). Interaction fractions and characterization of interactions of binding pocket residues of PDE10A protein with 71271414 throughout the MD simulation (d). Surface representation of **YS10** on the PDE10A protein (e). Ligand interaction diagram of **YS10** with the critical residues (f). Time-dependent protein-**YS10** contact panel throughout 200 ns MD simulations. The upper panel indicates the total contacts, while the lower panel indicates the constructed and broken interactions (g). Interaction fractions and characterization of interactions of binding pocket residues of PDE10A protein with **YS10** throughout MD simulations (h). This figure is created with BioRender and ChimeraX.

Table 3 shows the interaction energies of studied compounds at the PDE10A. Figure 5 illustrates the 2D protein–ligand contacts and interactions of **YS10** which is the most active compound through biological activity studies. The same protocol was also conducted for the known active compound (i.e., reference PubChem CID: 71271414). While Asp664, Ser667, Ile682, Tyr683, Phe686, Met703, Gln716, and Phe719 were found crucial residues for the contacts with the reference ligand, the corresponding amino acid residues were Asp664, Tyr683, and Phe719 with **YS10**.

**Table 3:** Docking and Average MM/GBSA Scores of Studied Compounds at Target Protein PDE10A (PDB ID: 8DI4)

**ligand**	**docking score** (kcal/mol)	**average MM/GBSA** (kcal/mol)
PubChem CID: 71271414 (Reference)	–7.67	–82.54 ± 5.09
**YS7**	–7.06	–60.90 ± 5.77
**YS12**	–7.17	–58.63 ± 4.17
**YS11**	–7.32	–57.11 ± 4.49
**YS5**	–7.24	–55.39 ± 6.50
**YS8**	–7.22	–52.28 ± 5.98
**YS6**	–6.89	–48.86 ± 6.70
**YS3**	–9.11	–47.88 ± 13.92
**YS10**	–8.09	–46.08 ± 8.74
**YS1**	–7.15	–45.95 ± 8.83
**YS2**	–7.35	–45.57 ± 7.10
**YS4**	–6.68	–41.65 ± 6.49
**YS9**	–7.95	–37.88 ± 6.21

**Figure 6:**
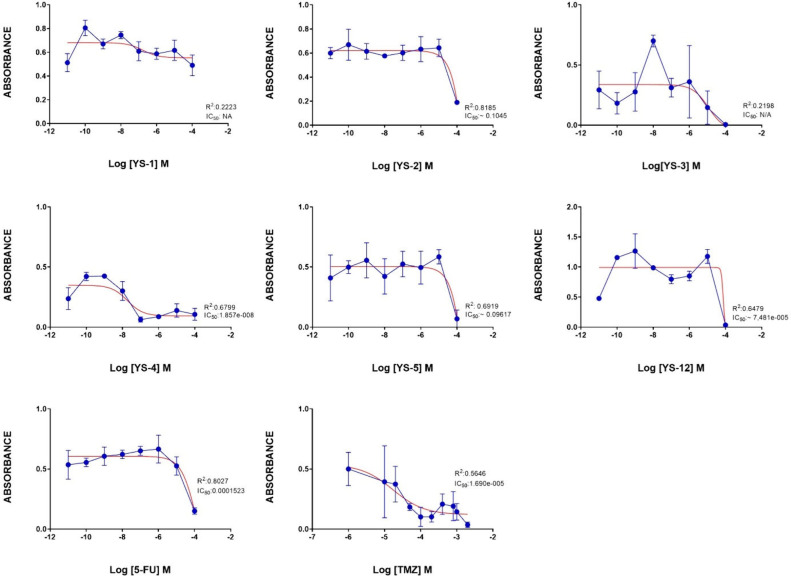
Antiproliferative effects of the positive control compounds 5-FU and Temozolomide (TMZ), and the studied ligands (**YS1–12**) in glioblastoma YKG1 cells. Data are presented as the mean ± standard error of the mean (SEM). IC_50_ values were calculated by Prism 8 software. IC_50_ value was considered NA when R^2^ value was less than 0.5. (**YS6–11** did not show activity against YKG-1 cells).

Glioblastoma and neuroblastoma cells were preferred in *in vitro* analysis because *A1AR* and *PDE10A* genes are predominantly expressed in the brain and central nervous system.^43,44^ The nTPM of these genes in YKG-1 and SH-SY5Y are shown in the supplementary figures S49–S52 (The Human Protein Atlas, https://www.proteinatlas.org). To identify which ligands were able to attenuate the proliferation of YKG-1 glioblastoma cells, we tested the cell viability activities of twelve synthesized ligands using an MTT assay. Table 4 shows the IC_50_ values of all ligands. Of all tryptamine-thiazolidin-4-one derivatives, particularly **YS1**, **YS2**, **YS3**, **YS4**, **YS5**, and **YS12** derivatives had inhibitory activities in glioblastoma cell line (YKG1). Of these six compounds, **YS4** was the most active inhibitor with the lowest IC_50_ value (IC_50_:18.57 nM), others had IC_50_ values in the μM level (Figure 6). Since the R^2^ values of **YS1** and **YS3** compounds were below 0.5, their IC_50_ values were shown as NA (not applicable) in Table 4.

**Table 4:** IC_50_ Values of **YS1-12** in Glioblastoma Cells (YKG-1) and Neuroblastoma Cells (SH-SY5Y) in μM

**ligand**	**YKG-1 (μM)**	**SH-SY5Y (μM)**
**5-FU**	152.30	3.40
**TMZ**	16.90	1.51
**YS1**	NA	NA
**YS2**	NA	NA
**YS3**	NA	56.86
**YS4**	**0.02**	NA
**YS5**	NA	74.01
**YS6**	NA	62.78
**YS7**	NA	7.715
**YS8**	NA	NA
**YS9**	NA	7.894
**YS10**	NA	**0.00044**
**YS11**	NA	18.78
**YS12**	74.81	NA

**Figure 7:**
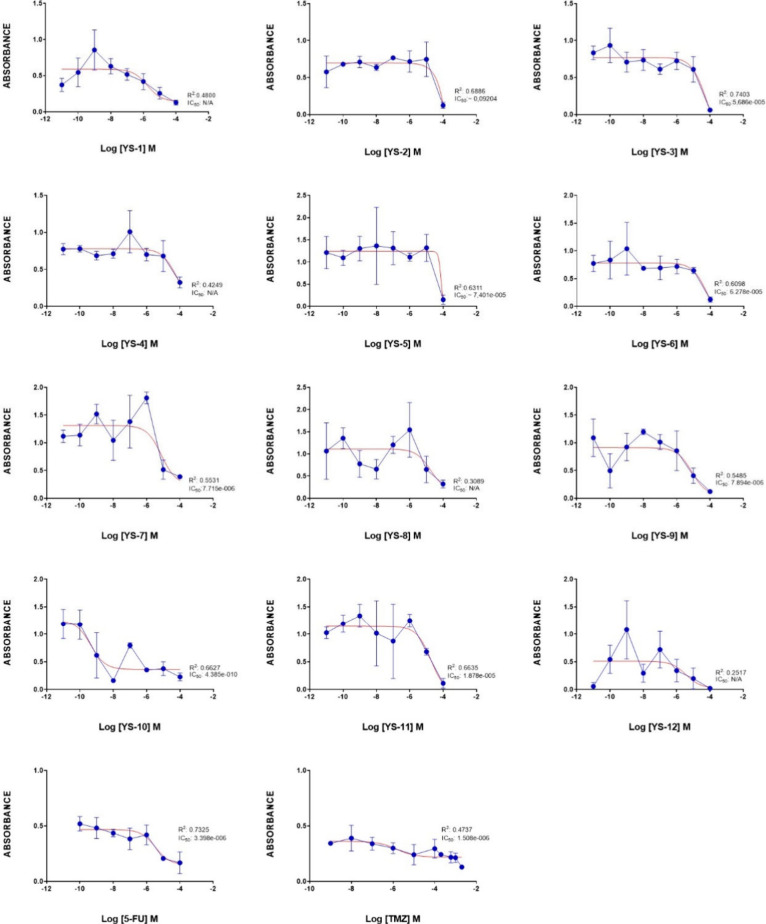
Antiproliferative effects of the positive control compounds 5-FU and TMZ, and the compounds **YS1–12** in neuroblastoma SH-SY5Y cells. Data are presented as the mean ± standard error of the mean (SEM). IC_50_ values were calculated by Prism 8 software. IC_50_ value was considered NA when R^2^ value was less than 0.5.

Most tryptamine-thiazolidin-4-one derivatives (**YS1–12**) had inhibitory activity in the neuroblastoma cell line (SHSY5Y). **YS10** was the most active compound with submicromolar concentration (IC_50_: 0.44 nM), others had IC_50_ values in the μM level (Figure 7). Additionally, the R^2^ values of **YS1**, **YS4**, **YS8** and **YS12** molecules were below 0.5, their IC_50_ values were shown as NA (not applicable) in Table 4.

**Figure 8:**
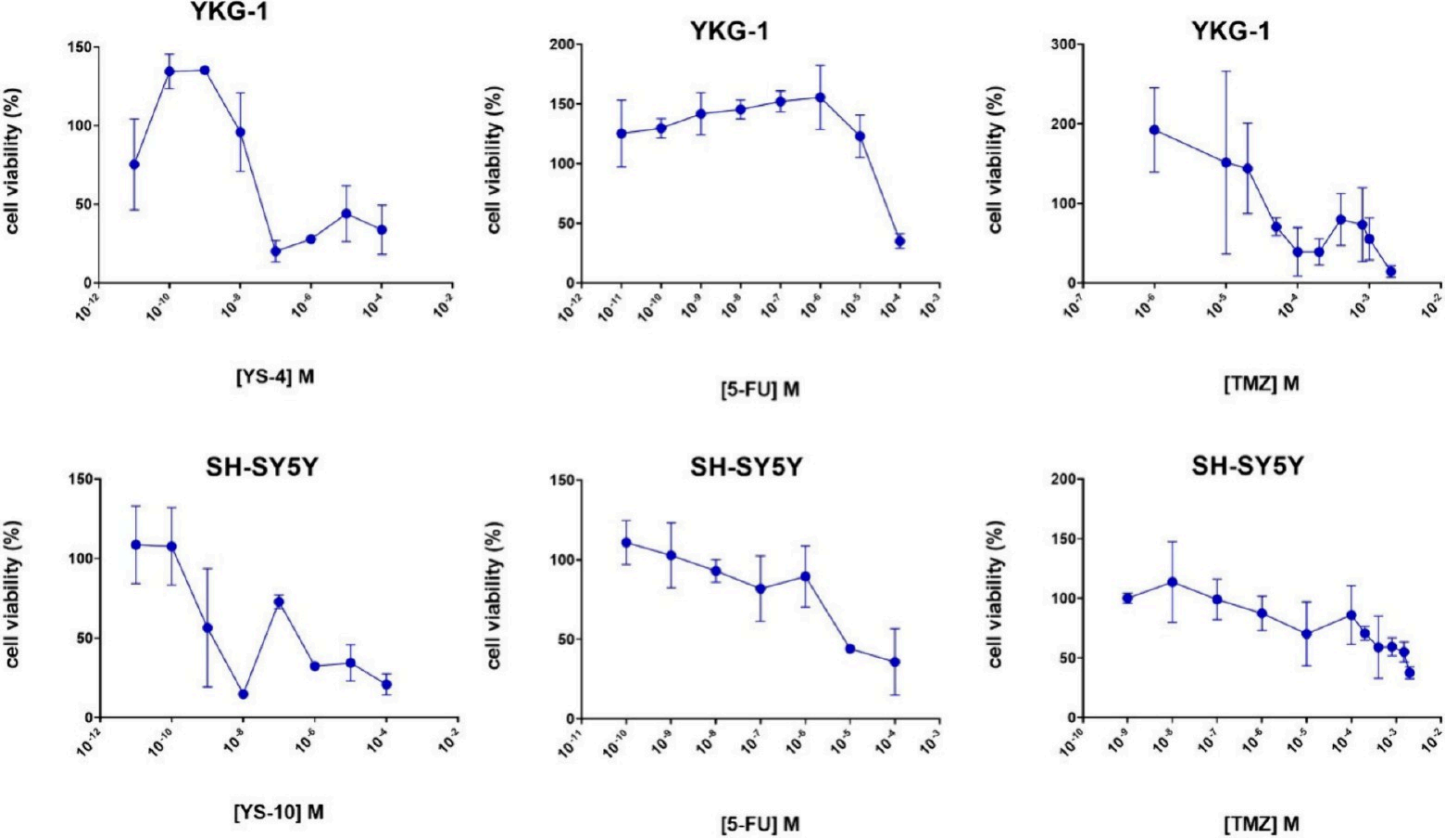
Figure presents the effects of selected hit compounds and positive controls on cell viability across different cell lines (SH-SY5Y, YKG1) under different treatment conditions. The panels YS-4_YKG1 and YS-10_SH-SY5Y show the dose-dependent impact of the compounds YS-4 and YS-10 on cell viability, respectively. The SH-SY5Y and YKG1 panels display the effects of control compounds 5-FU and TMZ on cell viability, indicating a reduction in viability with increasing concentrations. . Each data point represents the mean cell viability percentage, with error bars indicating the Standard Error of the Mean (SEM). Statistical analyses confirm significant dose–response relationships, demonstrating substantial decreases in cell viability at higher concentrations for both the test and control compounds.

Figure 8 displays the cell viability (%) of the top active molecules **YS4** and **YS10** in YKG-1 and SH-SY5Y cell lines, respectively, in comparison with the control compounds 5-FU and TMZ in both cell lines.

In neuroblastoma cell line (SH-SY5Y), when we compare the effectiveness of the **YS10** with FDA approved drugs for brain tumors treatment as positive control, the effect of **YS10** on cell viability was found to be 8.5-fold greater than 5-FU and 3.8-fold greater than TMZ. *In vitro* cell line studies of the derivatives revealed that **YS4** had better and selective anticancer potential on glioblastoma cells (YKG1) compared to 5-FU and TMZ as positive control drugs. The effect of **YS4** on cell cytotoxicity was found to be more effective than 5-FU and TMZ.

**Figure 9:**
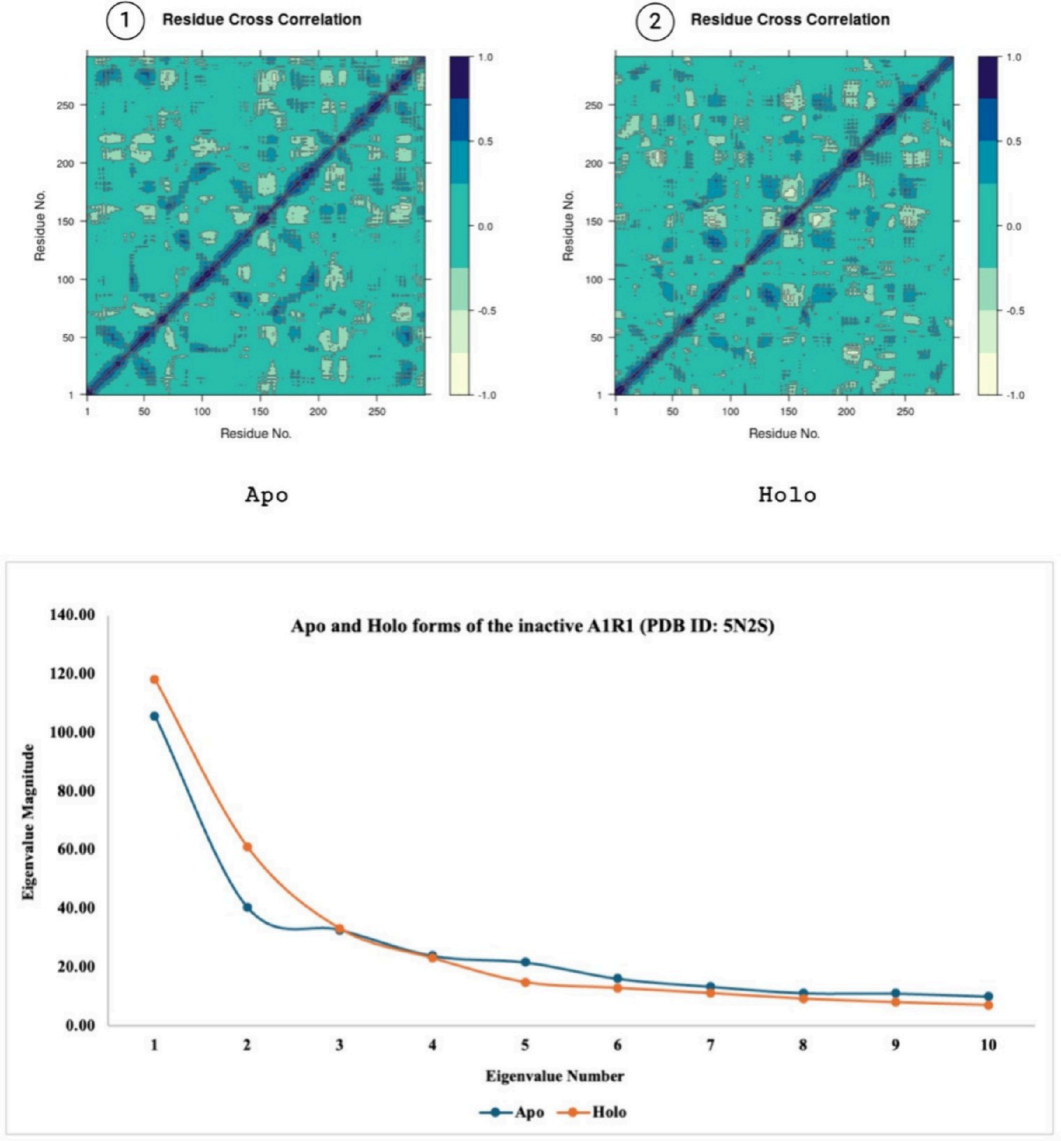
Panels 1 and 2 show the residue cross correlation maps for apo and holo states. PCA showing the eigenvalue magnitudes of 50 ns MD simulations (bottom) of apo and holo forms of the inactive A1R1 (PDB ID: 5N2S). Created with BioRender.com.

**Figure 10:**
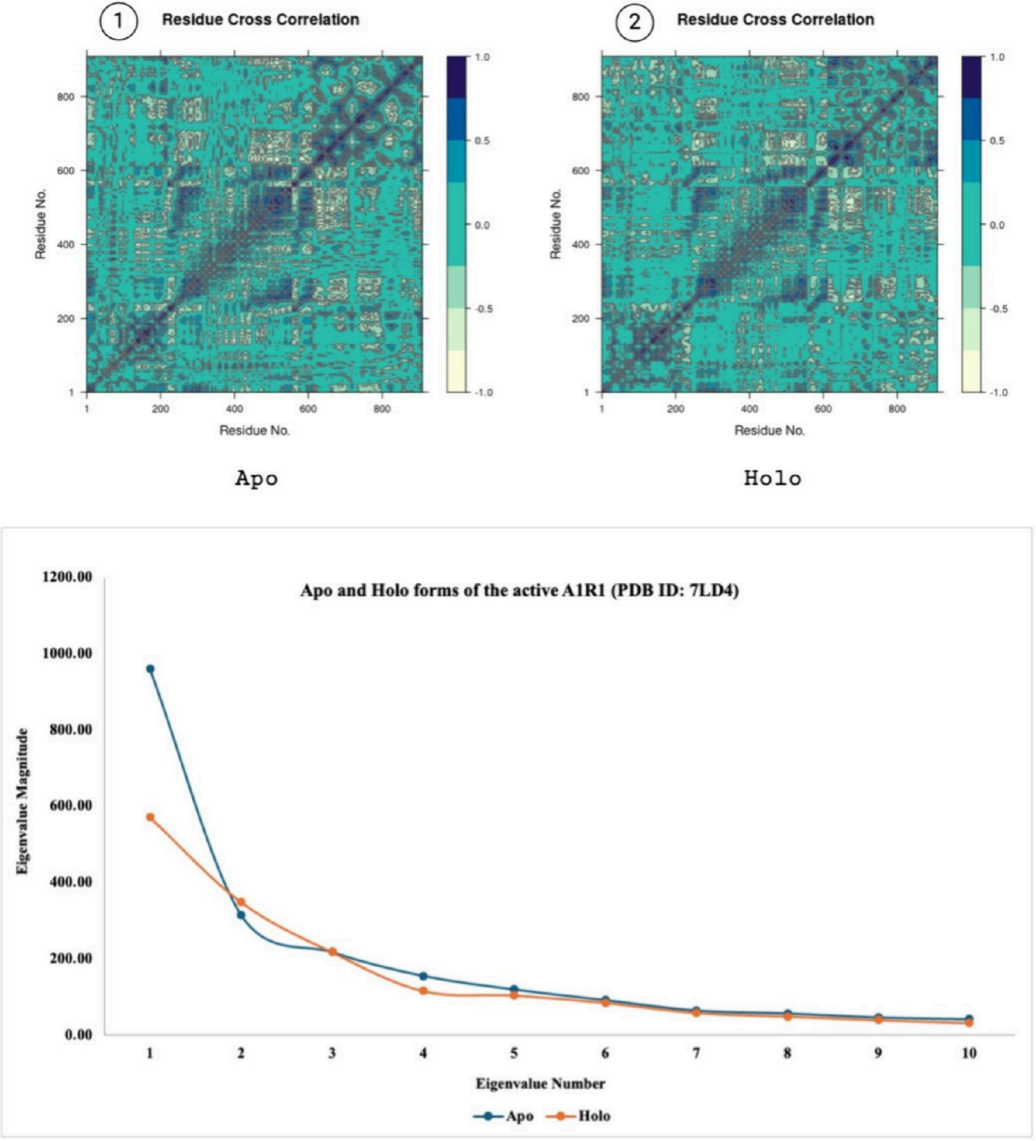
Panels 1 and 2 show the residue cross correlation maps for apo and holo states. PCA showing the eigenvalue magnitudes of 50 ns MD simulations (bottom) of apo and holo forms of the active A1R1 (PDB ID: 7LD4). Created with BioRender.com.

**Figure 11:**
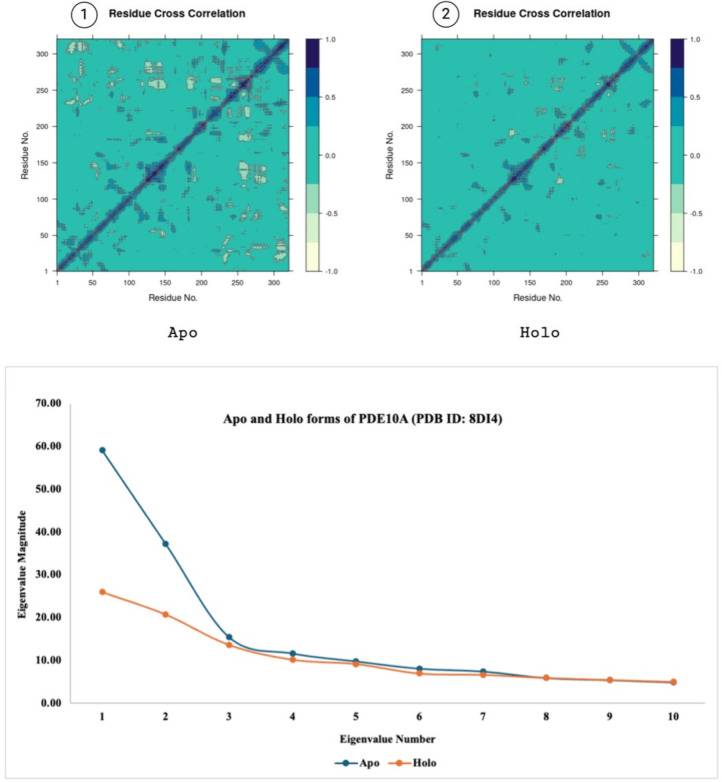
Panels 1 and 2 show the residue cross correlation maps for apo and holo states. PCA showing the eigenvalue magnitudes of 50 ns MD simulations (bottom) of apo and holo forms of PDE10A (PDB ID: 8DI4). Created with BioRender.com.

Building on the synthesis and *in vitro* evaluation of the tryptamine-thiazolidin-4-one derivatives, further computational studies were conducted to understand the dynamical behavior of the most promising compounds to the binding pocket and global structure of the proteins. Temperature-based replica exchange molecular dynamics (t-REMD) simulations were utilized to assess the stability and interactions of these molecules with their target proteins. In the t-REMD, the top biologically active molecules, **YS4** and **YS10**, identified from the *in vitro* study, displayed a similar trend at each replica throughout the temperature range of 300–320 K at both molecules’ relevant target proteins. Supplementary tables S18–S20 and figures S53–S55 illustrate the principal component analysis (PCA) results, further emphasizing the conformational changes observed at the target proteins with regard to temperature. The magnitudes of the eigenvalues displayed are compounds among replicas at each eigenvector index with a higher motion associated with higher temperatures. It has been discussed in earlier studies that 10 indices of mobility are sufficient to substantiate the motion divergence throughout the simulation^45^ thus, 10 eigenvector indices were plotted. Figures 9–11 highlight the differences between the apo and holo forms of the target proteins and show the results obtained from the PCA performed on 50 ns MD simulations. With minor exceptions the apo form was generally found to be more mobile than the holo form due to the holo form being more stabilized with the ligand bound to its active site.

**Figure 12:**
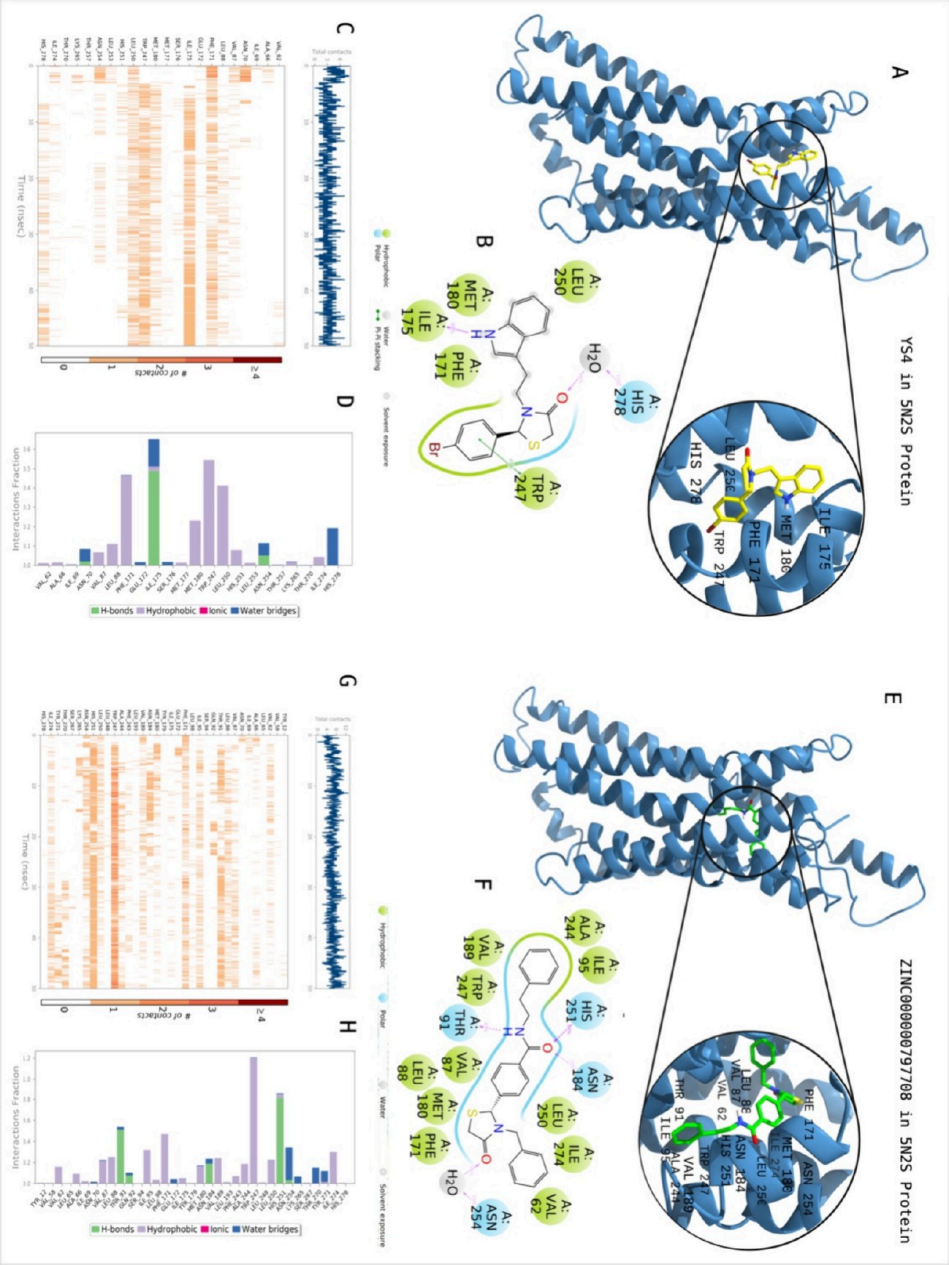
Interactions of compound ZINC000000797708 with the critical residues on the A1AR protein (PDB ID: 5N2S) in comparison with the hit molecule **YS4**. Surface representation of **YS4** on the A1AR protein (a). Ligand interaction diagram of **YS4** with the critical residues (b). Time-dependent protein-**YS4** contact panel throughout 50 ns MD simulations. The upper panel indicates the total contacts, while the lower panel indicates the formed and broken interactions (c). Interaction fractions and characterization of interactions of binding pocket residues of A1AR protein with ZINC000000797708 throughout MD simulations (d). Surface representation of ZINC000000797708 on the A1AR protein (a). Ligand interaction diagram of ZINC000000797708 with the critical residues (b). Time-dependent protein- ZINC000000797708 contact panel throughout 50 ns MD simulations. The upper panel indicates the total contacts, while the lower panel highlights the formed and broken interactions (c). Interaction fractions and characterization of interactions of binding pocket residues of A1AR protein with ZINC000000797708 throughout the MD simulation (d). This figure is created with BioRender and ChimeraX.

To explore more active compounds, a similarity-based analysis was also conducted to find analogs of the top-active compounds **YS4** and **YS10**. For this purpose, we used the SwissSimilarity server (http://www.swisssimilarity.ch/). **YS4** and **YS10** were used as query input structures, and 400 molecules with a Tanimoto coefficient of 0.75 or higher were obtained for each of the hit molecules. Subsequently, Glide/XP docking was performed on these compounds. The docking results of all molecules are represented in the Supplementary tables S21–S23. The top-3 analog molecules from each query compound were passed through 50 ns MD simulations, and the output data presented in Table 5. Figure 12 shows the 2D protein–ligand interactions and contacts of ZINC000000797708 which is the top scoring molecule with an average MM/GBSA score of −99.79 kcal/mol. Crucial contacts were formed by Val62, Val87, Leu88, Thr91, Ile 95, Phe171, Met180, Asn184, Val189, Ala244, Trp247, Leu250, His251, Asn254, and Ile274 with ZINC000000797708. **YS4** was included for reference purposes and its interactions have been explained in detail above.

**Table 5:** Docking and Average MM/GBSA Scores of Analog Compounds of **YS4** and **YS10** at Target Proteins 5N2S, 7LD4, and 8DI4

**protein**	**ID**	**similarity score**	**docking score** (kcal/mol)	**average MM/GBSA score** (kcal/mol)
**5N2S**	**YS4**	**1.00**	**-7.45**	**–**64.07 ± 4.07
	ZINC000000797708	0.94	–11.40	–99.79 **± 7.86**
	ZINC000409174406	0.93	–11.03	–90.74 **± 8.14**
	ZINC000003328672	0.90	–11.02	–60.71 **± 5.13**
**7LD4**	**YS4**	**1.00**	**-6.64**	**-**59.17 ± 4.83
	ZINC000013161844	0.96	–9.33	–78.57 **± 5.84**
	ZINC000000753084	0.96	–9.55	–71.03 **± 7.46**
	ZINC000019927337	0.90	–10.13	–64.10 **± 4.34**
**8DI4**	**YS10**	**1.00**	**-8.09**	**-**46.08 ± 8.74
	ZINC000000660131	0.94	–10.58	–78.75 **± 7.71**
	ZINC000408612171	0.87	–10.54	–74.99 **± 9.43**
	ZINC000012774036	0.88	–10.31	–61.52 **± 6.25**

**Figure 13:**
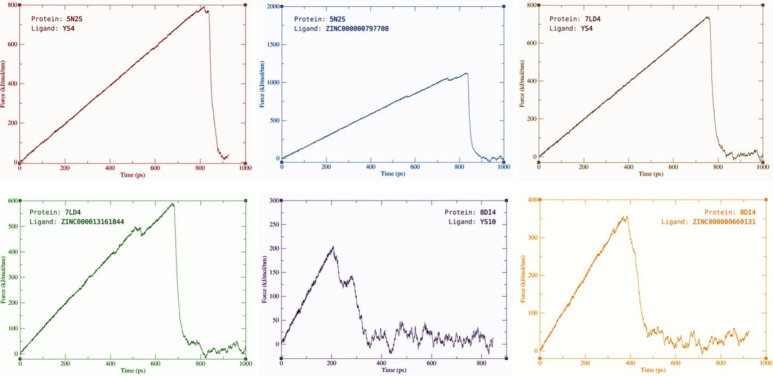
sMD simulations of the hit molecules and the relative top-analog molecule at the relevant target protein. Created with BioRender.com.

To further investigate active compounds dynamic and binding behavior at the target sites, steered MD simulations (sMD) were also conducted. The sMD simulations revealed ZINC000000797708 and ZINC000000660131 to require more force to be pulled from the relevant target protein than **YS4** and **YS10** respectively and separating at a later time point in the 1000 ps simulations. ZINC000013161844, on the other hand, proved to require less force to be pulled from the active form of A1R1 (PDB ID:7LD4) than YS4 and separates at an earlier time point around 650 ps. Figure 13 displays the force (kJ/(mol nm)) against the time (ps) for each hit molecule and its top similar compound at their relative target protein.

Indole and thiazolidine derivatives are found to exhibit potent anticancer activity against many human cancer cells and show numerous biological activities, including cytotoxic, antiviral, antimicrobial, anti-inflammatory.^46−48^ To that end, an ADME/Toxicity analysis was conducted using MetaDrug/MetaCore. The results, which indicate mainly suitable ADME/Tox properties, are presented in Supplementary Table S24.

In a study at 2022, the cytotoxic activity of thiazolidine derivatives were assessed against HeLa (cervical carcinoma), MCF-7 (breast carcinoma), and HuH-7 (liver carcinoma) cell lines.^46^*In vitro* DNA binding and cytotoxicity studies have shown that thiazolidine derivatives are potent anticancer drug candidates.^49^ Furthermore, it has been shown in a number of studies that indole derivatives are important in cancer by acting through mechanisms such as induction of apoptosis, regulation of estrogen receptor and inhibition of tyrosine kinase, NFkB/PI3/Akt/mTOR pathway.^50^ Parallel to these findings, in our study, it was observed that tryptamine-thiazolidin-4-one derivatives (**YS1**-**12**), which have promising interaction energies with the A1AR and PDE10A, had cytotoxic activity on glioblastoma and neuroblastoma cell lines.

In this study, tryptamine-thiazolidin-4-one derivatives (**YS1–12**) were synthesized via a one-pot three-component condensation reaction in 40–45% yield. The presence of the carbonyl group in the FT-IR spectrum at 1650–1670 cm^–1^ indicated the formation of the thiazolidinone ring. In the thiazolidin-4-one ring, one of the two protons belonging to the methylene group between the carbonyl group and the sulfur atom comes in around 3.69–3.70 ppm, the other around 3.79–3.80 ppm detected ^1^H NMR spectra as diastereotopic protons. In addition, a peak around 5.21–577 ppm in the ^1^H NMR spectrum indicates the hydrogen atom attached to the carbon atom between the nitrogen and sulfur atoms in the thiazolidin-4-one ring. These values at ^1^H NMR for the thiazolidin-4-one ring are consistent with those in the literature.^41,42^ In addition, when the tryptamine-thiazolidin-4-one structure is formed as a result of the reaction, it is seen in the ^1^H NMR spectrum of the synthesized compounds that both the methylene group hydrogens in the thiazolidinone ring and the methylene group hydrogens attached to the nitrogen atom in the tryptamine part are diastereotopic hydrogen atoms. One of the studies in the literature is in line with our opinion.^53^ Bhusnure et al. synthesized tryptophan-thiazolidin-4-one derivatives in two steps as anticonvulsants and antidepressants.^51^ For comparative purposes, we tried to synthesize the NL2 **(6)** coded compound in this literature according to their method, but we could not obtain it. The relevant literature shows that the peaks belonging to the thiazolidinone ring in the ^1^H NMR data are evaluated as unusual. The values of the 1H-NMR spectrum of the NL2 **(6)** coded compound given in the literature as follows; 1H-NMR: (DMSO): δ 3.64 (s, 2H, −CH_2_), 3.66 (s, 2H, −CH_2_), 3.82 (s, H, −CH), 3.87 (s, H, −CH), 5.35 (s, 1H, Ar–OH), 6.91–7.16 (d, 2H, Ar–H), 7.19–7.57 (d, 2H, Ar–H), 9.84 (s, 1H, NH), 12.04 (s, 1H, OH).

A comprehensive target prediction analysis was initially performed using 25 different disease QSAR models on MetaDrug/MetaCore followed by SwissTargetPrediction of the **YS1–12** synthesized ligands. Heart failure and hypertension were chosen as promising targets. To this end, the inactive (5N2S) and active (7LD4) forms of the A1AR and PDE10A (8DI4) were selected to measure the activity of the ligands. The results showed that the synthesized molecules **YS7**, **YS6**, **YS12**, **YS11,** and **YS10** have an average interaction energy (average MM/GBSA score) of −69.70, −67.98, −65.42, −64.03, and −62.76 kcal/mol, respectively, as opposed to the endogenous agonist adenosine in the active form of the A1AR protein (PDB ID: 7LD4) with a score of −62.26 kcal/mol (Table 2). Although none of the synthesized ligands showed better interaction energy compared to the cocrystallized ligand at the inactive form of the A1AR, **YS10**, **YS11**, and **YS7** showed promising average MM/GBSA scores of −75.80, −69.00, −67.94 kcal/mol, respectively (Table 1). In PDE10A, although synthesized compounds displayed weaker interaction energies than the cocrystallized reference molecule, **YS7** showed a favorable free binding energy of −60.90 kcal/mol (Table 3).

The comparison of synthesized compounds with control drugs, TMZ and 5-FU, is critical for evaluating their potential as anticancer agents. TMZ, a standard chemotherapeutic agent primarily used for treating glioblastoma, works by alkylating or methylating DNA, leading to DNA damage and initiating cell death pathways. However, its efficacy is often hindered by the development of resistance, highlighting the need for alternative treatments. Similarly, 5-FU, a well-established chemotherapeutic drug, is widely used for treating various cancers, including colorectal, breast, and head and neck cancers. It functions as a thymidylate synthase inhibitor, interfering with DNA synthesis and leading to cell death. However, its effectiveness in brain cancers is limited due to poor blood-brain barrier (BBB) penetration and systemic toxicity. *In vitro* analyses showed that at least half of the ligands demonstrated a suppressive effect on cell viability in glioblastoma cells. Notably, in neuroblastoma cells, nearly all ligands (except **YS8**) exhibited antiproliferative effects. In the neuroblastoma cell line (SH-SY5Y), the **YS10** showed significantly higher effectiveness in reducing cell viability compared to FDA-approved brain cancer treatments used as positive controls. Specifically, YS10 was found to be 8.5-fold more effective than 5FU and 3.8-fold more effective than TMZ. Additionally, *in vitro* studies on these synthesized compounds indicated that **YS4** exhibited superior and selective anticancer potential in glioblastoma cells (YKG1) compared to 5-FU and TMZ. The cytotoxic effect of the **YS4** molecule was at least a thousand fold more potent than that of 5-FU and TMZ. Further *in vitro* studies are necessary to elucidate the mechanisms of the ligands on the *A1AR* and *PDE10A* genes.

**YS4** and **YS10** compounds proved to be the most potent derivatives, with bromine and hydroxy substitutions respectively providing significant anticancer activity. Substitutions on the phenyl ring (fluorine, chlorine, bromine) and on the indole ring (methoxy, hydroxy) play critical roles in modulating biological activities. Hydroxy substitution generally enhances activity compared to methoxy substitution. Molecules obtained from the similarity-based screening of **YS4** and **YS10** showed much lower binding free energies and required more force and longer durations to be removed from their respective protein. The top molecule was ZINC000000797708 displayed about 31% decrease in binding free energy (−99.74 kcal/mol) than its original query compound **YS4** (−64.07 kcal/mol) in A1R1 (PDB ID; 5N2S). Overall the docking scores (Glide XP) of analog molecules where much higher than the original compounds **YS4** and **YS10** as shown in the supplementary tables S21–S23 thus, inviting further research that would extend into the *in vitro* to observe their biological activity in comparison with the existing biological activities obtained in this study.

## 3 Methods

**Figure 14:**
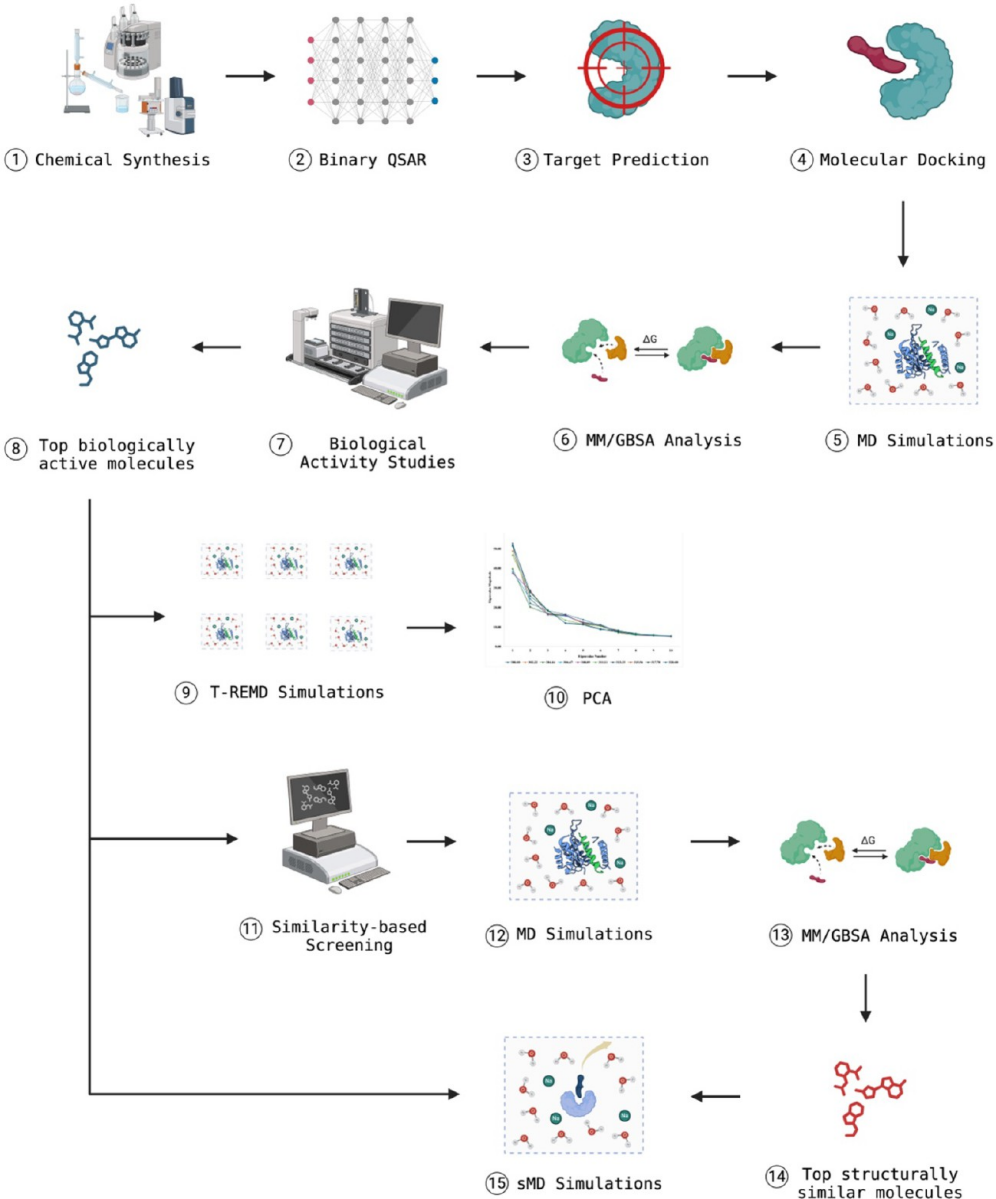
Figure illustrates a detailed workflow for the methodology followed in this study. The process begins with the chemical synthesis of compounds **(1)**, followed by the application of binary QSAR models to predict their biological activity **(2)**. Target prediction methods are used to identify potential biological targets **(3)**, which are then subjected to molecular docking studies to assess binding affinities **(4)**. MD simulations **(5)** and MM/GBSA analysis **(6)** are performed to evaluate the stability and binding free energies of the compound-target complexes. Experimental biological activity studies **(7)** validate these findings, leading to the identification of the top biologically active molecules **(8)**. T-REMD simulations **(9)** and PCA **(10)** further refine the understanding of the conformational space and dynamics of these molecules. Similarity-based screening **(11)** identifies structurally similar compounds, which undergo additional MD simulations **(12)** and MM/GBSA analysis **(13)** to assess their potential. The top structurally similar molecules **(14)** are selected, and sMD simulations **(15)** are conducted to study their unbinding processes and refine their binding modes, culminating in the identification of promising candidates for further development. Created with BioRender.com.

All solvents and chemicals were used as purchased without further purification. Thin-layer chromatography (TLC) was conducted on aluminum sheets coated with silica gel 60 F254, obtained from Merck, with visualization by UV lamp (254 or 360 nm). Column chromatography was carried out with silica gel 60 (particle size 0.040–0.063 mm, 230–400 mesh; Merck) and commercially available solvents. All melting points were measured on a Gallenkamp melting-point apparatus in open capillaries and were uncorrected. Fourier transform infrared spectroscopy (FTIR) analysis was studied with PerkinElmer Spectrum BX-II Model FTIR spectrophotometer to characterize synthesized molecules. The samples within KBr pellets were measured in the 4000 and 400 cm^–1^ range. The ^1^H and ^13^C NMR spectra were obtained on a Varian AS-400 NMR spectrometer with tetramethylsilane as an internal standard. The high-resolution mass spectra were measured on a Waters SYNAPT G1MS mass spectrometer. The methodology observed in this study is illustrated below (Figure 14).

### Synthesis of Tryptamine-Thiazolidone Derivatives (YS1–12)

A solution of tryptamine derivatives **1a**–**c** (1 mmol), benzaldehyde derivatives **2a**–**d** (1 mmol), and thioglycolic acid **3** (1.2 mmol) in dry benzene was refluxed under Dean–Stark apparatus for 18 h. Then the solvent was evaporated under reduced pressure, and the crude product was chromatographed using silica gel and ethyl acetate: hexane (1:1). The solvent was evaporated under reduced pressure, and the product was recrystallized from ether yielded tryptamine-thiazolidone derivatives (**YS1–12**).

#### 3-(2-(1*H*-Indol-3-yl) ethyl)-2-phenylthiazolidin-4-one (YS1)

Yield: 45%; mp: 155–156 °C; IR (KBr): ν 3213 (NH), 2933 (CH), 1655 (NHCO) cm^–1^; ^1^H NMR (400 MHz, CDCl_3_): δ 2.79–2.90 (1H, m, CH_2_), 2.99–3.11 (2H, m, CH_2_), 3.71 (1H, d, *J*: 15.4 Hz, OCCH_2_S), 3.82 (1H, dd, *J*: 15.5, 2.0 Hz, OCCH_2_S), 3.86–3.99 (1H, m, CH_2_), 5.37 (1H, d, *J*: 1.4 Hz, NCHS), 6.97 (1H, d, *J*: 2.1, ArH), 7.07 (1H, t, *J*: 7.7 Hz, ArH), 7.14–7.22 (3H, m, ArH), 7.32–7.38 (5H, m, ArH), 8.18 (1 H, s, NH); ^13^C NMR (400 MHz, CDCl_3_): δ 22.91, 33.09, 43.60, 64.16, 111.22, 112.44, 118.55 119.43 122.02, 122.15, 127.12, 127.29 (2C), 128.98 (2C), 129.19, 136.24, 139.27, 171.28; HR-MS (ESI+) C_19_H_19_N_2_OS ([M + H]^+^) Calc. 323.1218, Found 323.1220.

#### 3-(2-(1*H*-Indol-3-yl) ethyl)-2-(4-fluorophenyl) thiazolidin-4-one (YS2)

Yield: 47%; mp: 134–135 °C; IR (KBr): ν 3357 (NH), 2913 (CH), 1663 (NHCO) cm^–1^; ^1^H NMR (400 MHz, CDCl_3_): δ 2.79–2.90 (1H, m, CH_2_), 2.97–3.10 (2H, m, CH_2_), 3.70 (1H, d, *J*: 15.4 Hz, OCCH_2_S), 3.80 (1H, dd, *J*: 15.5, 2.0 Hz, OCCH_2_S), 3.86–3.98 (1H, m, CH_2_), 5.28 (1H, s, NCHS), 6.96–7.04 (3H, m, ArH), 7.05–7.11 (3H, m, ArH), 7.20 (1H, t, *J*: 7.1 Hz, ArH), 7.36 (2H, dd, *J*: 8.1, 3.8 Hz, ArH), 8.22 (1H, s, NH); ^13^C NMR (400 MHz, CDCl_3_): δ 22.98, 33.05, 43.51, 63.54, 111.32, 112.37, 115.82, 116.04, 118.50, 119.48, 122.15 (2C) (d, *J*: 12.2 Hz), 127.07, 129.25 (2C) (d, *J*: 8.5 Hz), 134.95 (d, *J*: 3.3 Hz), 136.29, 162.99 (d, *J*: 248.7 Hz), 171.15; HR-MS (ESI+) C_19_H_18_N_2_OFS ([M + H]^+^) Calc. 341.1124, Found 341.1124.

#### 3-(2-(1*H*-Indol-3-yl) ethyl)-2-(4-chlorophenyl) thiazolidin-4-one (YS3)

Yield: 46%; mp: 138–139 °C; IR(KBr): ν 3277 (NH), 2938 (CH), 1669 (NHCO) cm^–1^; ^1^H NMR (400 MHz, CDCl_3_): δ 2.81–2.88 (1H, m, CH_2_), 2.96–3.08 (2H, m, CH_2_), 3.69 (1H, d, *J*: 15.2 Hz, OCCH_2_S), 3.79 (1H, dd, *J*: 15.6, 2.0 Hz, OCCH_2_S), 3.89–3.97 (1H, m, CH_2_), 5.23 (1H, s, NCHS), 6.98 (1H, d, *J*: 2.4 Hz, ArH), 7.02 (2H, d, *J*: 8.8 Hz, ArH), 7.08 (1H, t, *J*: 8.0 Hz, ArH), 7.20 (1H, t, *J*: 8.8 Hz, ArH), 7.29 (2H, d, *J*: 8.4 Hz, ArH), 7.35 (2H, t, *J*: 7.6 Hz, ArH), 8.13 (1H, s, NH); ^13^C NMR (400 MHz, CDCl_3_): δ 22.99, 32.98, 43.53, 63.49, 111.29, 112.41, 118.51, 119.54, 122.04, 122.27, 127.05, 128.68 (2C), 129.14 (2C), 134.97, 136.26, 137.81, 171.16; HR-MS (ESI+) C_19_H_17_N_2_OSClNa ([M + Na]^+^) Calc. 379.0648, Found 379.0648.

#### 3-(2-(1*H*-Indol-3-yl) ethyl)-2-(4-bromophenyl) thiazolidin-4-one (YS4)

Yield: 44%; mp: 149–150 °C; IR (KBr): ν 3263 (NH), 2932 (CH), 1666 (NHCO) cm^–1^; ^1^H NMR (400 MHz, CDCl_3_): δ 2.81–2.90 (1H, m, CH_2_), 2.96–3.11 (2H, m, CH_2_), 3.69 (1H, d, *J*: 15.4 Hz, OCCH_2_S), 3.79 (1H, dd, *J*: 15.5, 1.6 Hz, OCCH_2_S), 3.87–3.99 (1H, m, CH_2_), 5.21 (1H, s, NCHS), 6.94–7.00 (3H, m, ArH), 7.09 (1H, t, *J*: 7.4 Hz, ArH), 7.21 (1H, t, *J*: 7.4 Hz, ArH), 7.36 (2H, t, *J*: 7.9 Hz, ArH), 7.44 (2H, d, *J*: 8.3 Hz, ArH), 8.15 (1H, s, NH); ^13^C NMR (400 MHz, CDCl_3_): δ 22.99, 32.98, 43.55, 63.55, 111.30, 112.40, 118.51, 119.55, 122.05, 122.27, 123.13, 127.05, 128.96 (2C), 132.11 (2C), 136.26, 138.35, 171.17; HR-MS (ESI+) C_19_H_16_N_2_OSBr ([M-H]^+^) Calc. 401.0146, Found 401.0146.

#### 3-(2-(5-Methoxy-1*H*-indol-3-yl) ethyl)-2-phenylthiazolidin-4-one (YS5)

Yield: 46%; mp: 158–159 °C; IR (KBr): ν 3292 (NH), 2929 (CH), 1665 (NHCO) cm^–1^; ^1^H NMR (400 MHz, d_6_DMSO): δ 2.75–2.85 (1H, m, CH_2_), 2.97–3.08 (2H, m, CH_2_), 3.70 (1H, dd, *J*: 15.6 Hz, OCCH_2_S) 3.78 (3H, s, CH_3_), 3.81 (1H, dd, *J*: 15.4, 1.6 Hz, OCCH_2_S), 3.87–3.94 (1H, m, CH_2_), 5.33 (1H, d, *J*: 2.0 Hz, NCHS), 6.81 (1H, d, *J*: 2.4 Hz, ArH), 6.84 (1H, dd, *J*: 8.8, 2.4 Hz, ArH), 6.96 (1H, d, *J*: 2.4 Hz, ArH), 7.12–7.17 (2H, m, ArH), 7.24 (1H, d, *J*: 8.8 Hz, ArH), 7.31–7.36 (3H, m, ArH), 7.94 (1H, s, NH); ^13^C NMR (400 MHz, CDCl_3_): δ 23.04, 33.06, 43.42, 55.86, 64.13, 100.19, 111.97, 112.29, 112.62, 122.75, 127.22 (2C), 127.44, 128.93 (2C), 129.13, 131.32, 139.35, 154.03, 171.24; HR-MS (ESI+) C_20_H_19_N_2_O_2_S ([M-H]^+^) Calc. 351.1167, Found 351.1167.

#### 2-(4-Fluorophenyl)-3-(2-(5-methoxy-1*H*-indol-3-yl) ethyl) thiazolidin-4-one (YS6)

Yield: 45%; mp: 165–166 °C; IR(KBr): ν 3294 (NH), 2979 (CH), 1651 (NHCO) cm^–1^; ^1^H NMR (400 MHz, CDCl_3_): δ 2.75–2.86 (1H, m, CH_2_), 2.96–3.06 (2H, m, CH_2_), 3.70 (1H, d, *J*: 15.4 Hz, OCCH_2_S), 3.78 (1H, d, *J*: 15.6, 2.0 Hz, OCCH_2_S), 3.79 (3H, s, CH_3_), 3.85–3.96 (1H, m, CH_2_), 5.27 (1H, s, NCHS), 6.80 (1H, d, *J*: 2.2 Hz, ArH),6.86 (1H, dd, *J*: 8.8, 2.4 Hz, ArH), 6.96 (1H, d, *J*: 2.3 Hz, ArH), 7.00 (2H, t, *J*: 8.5 Hz, ArH), 7.08 (2H, dd, *J*: 8.7, 5.3 Hz, ArH), 7.25 (2H, d, *J*: 8.8 Hz, ArH), 7.94 (1H, s, NH); ^13^C NMR (400 MHz, CDCl_3_): δ 23.08, 33.02, 43.32, 55.83, 63.53, 100.22, 112.02, 112.27, 112.63, 115.77, 115.99, 122.76 (2C), 127.44, 129.22 (2C) (d, *J*: 8.4 Hz), 131.34, 135.01 (d, *J*: 3.0 Hz), 154.09, 171.10; HR-MS (ESI+) C_20_H_20_N_2_O_2_FS ([M + H]^+^) Calc. 371.1230, Found 371.1229.

#### 2-(4-Chlorophenyl)-3-(2-(5-methoxy-1*H*-indol-3-yl) ethyl) thiazolidin-4-one (YS7)

Yield: 45%; mp: 178–179 °C; IR(KBr): ν 3269 (NH), 2933 (CH), 1654 (NHCO) cm^–1^; ^1^H NMR (400 MHz, CDCl_3_): δ 2.72–2.86 (1H, m, CH_2_), 2.97–3.05 (2H, m, CH_2_), 3.69 (1H, d, *J*: 15.6 Hz, OCCH_2_S), 3.78 (1H, dd, *J*: 15.6, 2.0 Hz, OCCH_2_S), 3.79 (3H, s, CH_3_), 3.88–3.95 (1H, m, CH_2_), 5.25 (1H, s, NCHS), 6.79 (1H, d, *J*: 2.0 Hz, ArH), 6.86 (1H, dd, *J*: 8.8, 2.4 Hz, ArH), 6.95 (1H, d, *J*: 2.0 Hz, ArH), 7.02 (2H, d, *J*: 8.8 Hz, ArH), 7.26 (1H, d, *J*: 2.4 Hz, ArH), 7.28 (2H, d, *J*: 8.4 Hz, ArH), 7.98 (1H, s, NH); ^13^C NMR (400 MHz, CDCl_3_): δ 23.12, 32.98, 43.37, 55.82, 63.49, 100.16, 112.05, 112.20, 112.65, 122.78, 127.41, 128.66 (2C), 129.10 (2C), 131.34, 134.96, 137.82, 154.09, 171.16; HR-MS (ESI+) C_20_H_20_N_2_O_2_SCl ([M + H]^+^) Calc. 387.0934, Found 387.0933.

#### 2-(4-Bromophenyl)-3-(2-(5-methoxy-1*H*-indol-3-yl) ethyl) thiazolidin-4-one (YS8)

Yield: 47%; mp: 185–186 °C; IR(KBr):ν 3321 (NH), 2932 (CH), 1670 (NHCO) cm^–1^; ^1^H NMR (400 MHz, CDCl_3_): δ 2.78–2.87 (1H, m, CH_2_), 2.96–3.06 (2H, m, CH_2_), 3.69 (1H, d, *J*: 15.5 Hz, OCCH_2_S), 3.78 (1H, dd, *J*: 15.6, 2.0 Hz, OCCH_2_S), 3.79 (3H, s, CH_3_), 3.87–3.96 (1H, m, CH_2_), 5.23 (1H, s, NCHS), 6.79 (1H, d, *J*: 2.5 Hz, ArH), 6.86 (1H, dd, *J*: 8.8, 2.3 Hz, ArH), 6.92–6.97 (3H, m, ArH), 7.25 (1 H, d, *J*: 8.8 Hz, ArH), 7.44 (2H, d, *J*: 8.4 Hz, ArH), 7.98 (1H, s, NH); ^13^C NMR (400 MHz, CDCl_3_): δ 23.13, 32.98, 43.38, 55.84, 63.55, 100.15, 112.07, 112.17, 112.65, 122.80, 123.11, 127.40, 128.93 (2C), 131.35, 132.06 (2C), 138.35, 154.08, 171.19; HR-MS (ESI+) C_20_H_20_N_2_O_2_SBr ([M + H]^+^) Calc. 433.0408, Found 433.0390.

#### 3-(2-(5-Hydroxy-1*H*-indol-3-yl) ethyl)-2-phenylthiazolidin-4-one (YS9)

Yield: 40%; yellow oil; IR(KBr): ν 3400–3100 (broad OH), 3294 (NH), 2938 (CH), 1651 (NHCO) cm^–1^; ^1^H NMR (400 MHz, *d*6-DMSO): δ 2.53–2.59 (1H, m, CH_2_), 2.74–2.86 (2H, m, CH_2_), 3.67 (1H, d, *J*: 15.6 Hz, OCCH_2_S), 3.68–3.73 (1H, m, CH_2_), 3.85 (1H, d, *J*: 15.6 Hz, OCCH_2_S), 5.71 (1H, s, NCHS), 6.58 (1H, dd, *J*: 8.4, 1.2 Hz, ArH), 6.69 (1H, s, ArH), 6.95 (1H, s, ArH), 7.10 (1H, d, *J*: 8.4 Hz, ArH), 7.30–7.39 (5H, m, ArH), 8.58 (1H, s, NH), 10.48 (1H, s, OH); ^13^C NMR (400 MHz, CDCl_3_): δ 22.94, 33.16, 43.79, 64.41, 103.04, 111.60, 111.95, 112.13, 123.02, 127.36 (2C), 127.90, 129.01 (2C), 129.24, 131.35, 139.04, 149.89, 171.86; HR-MS (ESI+) C_19_H_19_N_2_O_2_S ([M + H]^+^) Calc. 339.1167, Found 339.1169.

#### 2-(4-Fluorophenyl)-3-(2-(5-hydroxy-1*H*-indol-3-yl) ethyl) thiazolidin-4-one (YS10)

Yield: 41%; yellow oil; IR(KBr): ν 3400–3100 (broad OH), 3251 (NH), 2937 (CH), 1657 (NHCO) cm^–1^; ^1^H NMR (400 MHz, CDCl_3_): δ 2.67–2.72 (1H, m, CH_2_), 2.82–2.89 (2H, m, CH_2_), 3.63 (1H, d, *J*: 15.2 Hz, OCCH_2_S), 3.71 (1H, dd, *J*: 15.6, 2.0 Hz, OCCH_2_S), 3.79–3.86 (m, 1H, CH_2_), 5.20 (1H, s, NCHS), 6.67 (1H, dd, *J*: 8.8, 2.4 Hz, ArH), 6.84 (1H, d, *J*: 2.4 Hz, ArH), 6.86 (1H, d, *J*: 2.0 Hz, ArH), 6.95 (2H, d, *J*: 8.4 Hz, ArH), 7.16 (1H, d, *J*: 8.4 Hz, ArH), 7.39 (2H, d, *J*: 8.4 Hz, ArH), 8.79 (1H, s, NH), 9.92 (1H, s, OH); ^1^H NMR (400 MHz, *d*_6_-DMSO): δ 23.02, 32.48, 43.44, 61.95, 102.50, 110.22, 111.85, 112.13, 116.04, 116.25, 123.59 (2C), 128.03, 129.85 (2C) (d, *J*: 8.4 Hz), 131.18, 136.90 (d, *J*: 2.8 Hz), 150.68, 170.75; HR-MS (ESI+) C_19_H_16_N_2_O_2_FS ([M-H]^+^) Calc. 355.0917, Found 355.0918.

#### 2-(4-Chlorophenyl)-3-(2-(5-hydroxy-1*H*-indol-3-yl) ethyl) thiazolidin-4-one (YS11)

Yield: 41%; yellow oil; IR(KBr): ν 3400–3100 (broad OH), 3376 (NH), 2929 (CH), 1661 (NHCO) cm^–1^; ^1^H NMR (400 MHz, CDCl_3_): δ 2.68–2.75 (1H, m, CH_2_), 2.87–3.00 (2H, m, CH_2_), 3.71 (1H, dd, *J*: 15.6, 0.8 Hz, OCCH_2_S), 3.79 (1H, dd, *J*: 15.6, 2.0 Hz, OCCH_2_S), 3.83–3.90 (m, 1H, CH_2_), 5.23 (1H, s, NCHS), 6.81 (1H, dd, *J*: 8.8, 2.4 Hz, ArH), 6.87 (1H, d, *J*: 2.4 Hz, ArH), 6.90 (1H, d, *J*: 2.4 Hz, ArH), 7.04 (2H, d, *J*: 8.8 Hz, ArH), 7.19 (1H, d, *J*: 8.8, 0.8 Hz, ArH), 7.28 (2H, d, *J*: 8.4 Hz, ArH), 8.02 (1H, s, NH); ^1^H NMR (400 MHz, CDCl_3_): δ 23.02, 33.02, 43.67, 63.67, 102.97, 111.74, 111.95, 112.16, 123.00, 127.84, 128.75 (2C), 129.18 (2C), 131.38, 134.99, 137.71, 149.82, 171.50; HR-MS (ESI+) C_19_H_18_N_2_O_2_SCl ([M + H]^+^) Calc. 373.0778, Found 373.0778.

#### 2-(4-Bromophenyl)-3-(2-(5-hydroxy-1*H*-indol-3-yl) ethyl) thiazolidin-4-one (YS12)

Yield: 42%; yellow oil; IR(KBr): ν 3400–3100 (broad OH), 3294 (NH), 2925 (CH), 1657 (NHCO) cm^–1^; ^1^H NMR (400 MHz, CDCl_3_): δ 2.63–2.70 (1H, m, CH_2_), 2.85–2.94 (2H, m, CH_2_), 3.66 (1H, d, *J*: 15.6 Hz, OCCH_2_S), 3.74 (1H, dd, *J*: 15.6, 1.6 Hz, OCCH_2_S), 3.79–3.86 (m, 1H, CH_2_), 5.18 (1H, s, NCHS), 6.80 (1H, dd, *J*: 8.8, 2.0 Hz, ArH), 6.84 (1H, d, *J*: 2.0 Hz, ArH), 6.88 (1H, d, *J*: 1.6 Hz, ArH), 6.94 (2H, d, *J*: 8.4 Hz, ArH), 7.14 (1H, d, *J*: 8.8 Hz, ArH), 7.39 (2H, d, *J*: 8.4 Hz, ArH), 8.23 (1H, s, NH), 9.94 (1H, s, OH); ^1^H NMR (400 MHz, CDCl_3_): δ 23.00, 33.05, 43.71, 63.73, 102.98, 111.58, 112.02, 112.22, 123.05, 123.13, 127.84, 129.03 (2C), 131.35, 132.13 (2C), 138.20, 149.94, 171.62; HR-MS (ESI+) C_19_H_18_N_2_O_2_SBr ([M + H]^+^) Calc. 419.0252, Found 419.0253.

### Binary QSAR Analysis

The synthesized compounds **YS1–12** were subjected to the MetaDrug/MetaCore therapeutic activity binary-QSAR models for activity prediction for 25 diseases (https://portal.genego.com/).^38^ The parameters used to build each binary-QSAR model are explained in detail in the supplementary section, table S1.

### SwissTargetPrediction

All 12 synthesized compounds **YS1–12** were run through the SwissTargetPrediction (http://www.swisstargetprediction.ch) online tool to be compared with around 376,342 molecules in 2D and 3D formats already established as active against almost 3068 macromolecular targets. The similarity between the synthesized molecules and the vast molecule library determines their possible targets.^52^ The probability of arriving at one true target in the top 15 targets is determined to be more than 70% of the external compounds (Supplementary tables S6–S17).

### Protein Preparation

Human adenosine A1 receptor-Gi2-protein complex (PDB ID: 7LD4), the stabilized A1AR (PDB ID: 5N2S), and PDE10A (PDB ID: 8DI4) were processed through the Protein Preparation module^53^ in the Maestro molecular modeling package. The initial preprocessing involved assigning bond orders, adding hydrogens, establishing zero-order bonds to metals, and forming disulfide bonds. Subsequently, the Prime module^54,55^ was applied to address missing side chains and loops. Hydrogen bond assignments were carried out using PROPKA^56^ at pH 7.0, followed by protein minimization employing the OPLS3e force field.^57^

### Receptor Grid Generation

The receptor grid was constructed based on the cocrystallized ligand within each of the three proteins. The corresponding x, y, and z Cartesian coordinates of the grid center points were as follows: (87.76, 71.28, 79.79) for 7LD4, (104.78, 129.28, 46.41) for 5N2S, and (4.70, 14.48, 41.52) for 8DI4. The outer and inner box dimensions were set at 30 and 10 Å, respectively. All hydroxyl and thiol groups encompassed by the grid box were permitted to rotate freely.

### Ligand Preparation

The previously synthesized 12 ligands **YS1–12** were prepared via the LigPrep tool,^53^ where all possible 3D conformation states were produced through Epik^58,59^ at a pH of 7.0 ± 2.0 with the OPLS3e force field applied.^57^

### Molecular Docking

Glide’s XP (extra precision) ligand docking module was used to dock the ligands in each of the three proteins in succession.^60^ The ligand sampling was set to flexible with the Epik state penalties assigned to the docking scores.^59^ Postdocking minimization was carried out with ten poses included per ligand, and the threshold for rejecting minimized pose was designated at 0.50 kcal/mol.

### Molecular Dynamics (MD) Simulations

The top conformation of each ligand with the lowest docking energy merged with its respective protein was simulated. The initial step involved setting up the system; for 7LD4 and 5N2S proteins, a membrane was created using the membrane model POPC and aligned on each protein’s OPM structure, respectively. (Ligand-bound PDE10A complex structures were used directly in water models). Next, each ligand in complex with its respective protein was submerged within a TIP3P solvent model water box featuring 10 Å buffering edges.^61^ Counterions were strategically placed to achieve neutralization, and 0.15 M NaCl was added. All atoms were parametrized using the OPLS3e force field.^57^ Subsequent to system setup, MD simulations were executed using Desmond,^62^ employing specific parameters: a simulation duration of 50 ns (for 5N2S and 7LD4) and 200 ns (for 8DI4), a recording interval of 50 ps (for 5N2S and 7LD4) and 200 ps (for 8DI4), 1000 frames, and an NPT ensemble class utilizing the Nose-Hoover chain thermostat method at 310 K. The Martyna-Tobias-Klein barostat method^63^ was applied to maintain a constant pressure of 1.01325 bar, employing an isotropic coupling style to attain thermodynamic equilibrium. The RESPA integrator^64^ operated at intervals of 2.0 fs. Short-range electrostatic and van der Waals interactions were subject to a 9 Å radius cutoff, while long-range interactions were computed through the particle mesh Ewald method with periodic boundaries.

### Molecular Mechanics with Generalized Born and Surface Area Solvation (MM/GBSA)

The average binding energy between ligands and proteins was determined using the MM/GBSA method, which is incorporated into Prime.^54,55^ To achieve this, the MD simulation trajectory was sampled at intervals of 10 for a total of 1000 frames. From these, 100 frames were extracted to compute each compound’s average MM/GBSA score and standard deviation. These MM/GBSA scores were derived employing the VSGB 2.0 implicit solvation model,^65^ which evaluates the binding free energy using MD simulation snapshots featuring an explicit solvent in the protein–ligand complex. The OPLS3e force field^57^ was employed in this calculation.

### Cell Viability Assay

The activity measurements against tryptamine-thiazolidin-4-one derivatives (YS1–12) were performed using a glioblastoma (YKG-1), and a neuroblastoma (SH-SY5Y) cell line. Concentrations of 12 different synthesized ligands, ranging from 100 μM to 10 pM, were tested for 72 h of treatment. Additionally, two control compounds were included: TMZ, tested at dilutions of 2000 μM, 1000 μM, 800 μM, 400 μM, 200 μM, 100 μM, 50 μM, 20 μM, 10 μM, and 1 μM; and 5-FU, tested at dilutions of 100 μM, 10 μM, 1 μM, 100 nM, 10 nM, 1 nM, and 100 pM.^66,67^ The protocol was summarized as follows: 10,000 cells were seeded into a sterile 96-well plate and incubated at 37 °C for 24 h in an incubator with 5% CO_2_ and 95% humidity. Later, the medium was removed, and ligand-containing cell media was added and incubated for 72h. Later, 10 μL of 5 mg/mL MTT solution was added to each well and incubated for 3 h at 37 °C. Finally, 100 μL of solubilization buffer was added to each well to dissolve the formazan crystals formed and an additional 15 min of incubation was done at room temperature. After incubation, absorbance was measured at a wavelength of 570 nm in a Variskan LUX microplate reader (ThermoFisher Scientific, MA, USA). Percent cell viability scores were evaluated by normalizing the data to untreated cells on the corresponding day of incubation.

### t-REMD Simulations

The t-REMD simulations were performed using Desmond with a total of 10 replicas. A parallel tempering method (REMD) was employed, utilizing a linear temperature profile to ensure effective sampling spanning across a temperature range of 300–320 K. Each simulation was conducted for a duration of 50 ns, with data recorded at 10 ps intervals, resulting in 1000 frames. The simulations were carried out under the NPT ensemble class, maintaining a pressure of 1.01325 bar. Prior to the simulation, the model was thoroughly relaxed.^62^

### PCA

PCA was performed using the Bio3D library^68^ in R Studio to analyze the results obtained from the t-REMD simulations and 50 ns MD simulations of apo and holo forms of each of the target proteins. The Bio3D package facilitated the extraction and analysis of essential motions from the 1000 frames generated during the simulations. By constructing a covariance matrix of atomic fluctuations, the dominant modes of motion were identified, highlighting the key conformational changes within the system. This analysis provided valuable insights into the structural dynamics and functional mechanisms, further enhancing the understanding of the molecular behavior under varying temperature conditions.

### Similarity-Based Screening

Using the Swiss Similarity tool (http://www.swisssimilarity.ch),^69,70^ a comprehensive virtual screening was conducted to identify structurally similar compounds to the top biologically active molecules, **YS4** and **YS10**. This screening yielded 400 molecules for each hit molecule, all exhibiting a Tanimoto coefficient of 0.75 or higher. The high Tanimoto coefficient indicates a significant structural similarity to **YS4** and **YS10**, suggesting these compounds may share similar biological activities.

### Molecular Docking, MD Simulations, and MM/GBSA Analysis

Following the Swiss Similarity screening, the 400 structurally similar molecules were subjected to molecular docking studies using Glide XP.^60^ This process identified the top three molecules with the highest docking scores. These top candidates were then subjected to 50 ns MD simulations using Desmond^62^ to evaluate their stability and interactions within the binding site. Subsequently, MM/GBSA analysis was performed using Prime^54,55^ to calculate the free energy of binding for these molecules. The parameters implemented in Glide XP, MD simulations, and MM/GBSA analysis are explained in detail above. This comprehensive approach allowed for a detailed assessment of the binding affinities and potential efficacy of the top three candidate molecules.

### sMD Simulations

The sMD simulations using GROMACS were performed to evaluate the binding interactions and stability of **YS4**, **YS10**, and the top-scoring molecules obtained from Swiss Similarity within their respective target proteins. Specifically, **YS4** was studied in the inactive form of A1R1 (PDB ID: 5N2S) using a pulling force of 100 kJ·mol^–1^·nm^–1^ and a duration of 1000 ps. For the top-scoring molecule ZINC000000797708, a pulling force of 150 kJ·mol^–1^·nm^–1^ was required (as 100 kJ·mol^–1^·nm^–1^ was insufficient to pull the ligand from the protein) with a duration of 1000 ps. Additionally, **YS4** and ZINC000013161844 were analyzed in the active form of A1R1 (PDB ID: 7LD4) using a force of 100 kJ·mol^–1^·nm^–1^ and a duration of 1000 ps. Finally, **YS10** and ZINC000000660131 were investigated within PDE10A (PDB ID: 8DI4) under the same conditions of 100 kJ·mol^–1^·nm^–1^ force and 1000 ps duration. These sMD simulations provided insights into the dynamic interactions and binding forces of the ligands within their respective targets, contributing to the understanding of their potential efficacy and stability.

### ADME/Toxicity Analysis

To evaluate the drug metabolism and pharmacokinetics of the final potential inhibitors, ADME/T predictions were performed using MetaDrug/MetaCore ADME/T QSAR models (https://portal.genego.com). These models assess drug candidates based on their physicochemical and pharmacokinetic properties before reaching the preclinical phase, including factors such as blood-brain penetration, lipophilicity, human serum protein binding, affinity to human serum albumin, and water solubility. Additionally, MetaDrug/MetaCore toxicity QSAR models (https://portal.genego.com) provide 26 independent toxicity filters to further analyze pharmacokinetic profiles. Detailed parameters of the model building are provided in the supplementary tables S3–S4.

## 4 Conclusions

In conclusion, the synthesis of tryptamine-thiazolidin-4-one derivatives (**YS1–12**) demonstrated the formation of the thiazolidinone ring, confirmed through FT-IR and 1H-NMR spectroscopy. These derivatives showed significant potential in targeting heart failure and hypertension, with specific compounds such as **YS7**, **YS6**, **YS12**, **YS11**, and **YS10** displaying promising interaction energies with the A1AR protein. Despite none surpassing the endogenous agonist adenosine in interaction energy for the inactive form of A1AR, **YS10**, **YS11**, and **YS7** still presented favorable scores. In PDE10A, **YS7** exhibited a notable free binding energy. Similarity-based screening highlighted ZINC000000797708 as a top molecule with a significant increase in free binding energy. *In vitro* analyses revealed the suppressive effects of these ligands on cell viability in glioblastoma and neuroblastoma cells, with **YS10** and **YS4** showing remarkable antiproliferative effects compared to FDA-approved treatments. These findings warrant further *in vitro* studies to elucidate the mechanisms of these ligands on *A1AR* and *PDE10A* genes, potentially paving the way for new therapeutic avenues in treating these diseases.

## Supporting Information Available

FT-IR ^1^H NMR, ^13^C NMR, and HRMS Spectra of Synthesized Tryptamine-thiazolidin-4-one derivatives **YS1**-**12**, binary QSAR disease model parameters, MetaDrug/MetaCore Disease QSAR Model results of **YS1**-**YS12**, SwissTargetPrediction results of **YS1**-**YS12**, PCA results, Glide XP results of molecules obtained from the Swiss Similarity server, and ADME/T results (PDF)
Tables: Metacore/Metadrug QSAR models, SwissTargetPredictions, Targets, Eigenvalues, Glide XP docking scores, Metacore/MetaDrug ADME/T Analysis (XLSX)
