# Supplementary material for: Synthesis of Tryptamine-Thiazolidin-4-one Derivatives and the Combined In Silico and In Vitro Evaluation of their Biological Activity and Cytotoxicity
Source: ACS Omega. 2024 Oct 23;9(44):44262–81. doi: 10.1021/acsomega.4c04456 (PMC11541526; doi:10.1021/acsomega.4c04456)
Supplement: Supplementary file 1 — ao4c04456_si_001.pdf [file ao4c04456_si_001.pdf]

Supporting Information

Synthesis of Tryptamine-Thiazolidin-4-one Derivatives and the Combined *in silico* and *in vitro* Evaluation of their Biological Activity and Cytotoxicity

Seher Aydın[a], Yavuz Ergün[b,*], Salma Ghazy[c,d], Asuman Çelebi[e], Turker Kilic[f], Timuçin Avşar[e], Serdar Durdağı[c,d,g,*]

[a]Dokuz Eylul University, The Graduate School of Natural and Applied Sciences, Kaynaklar Campus, 35160 Buca, Izmir, Türkiye

[b]Dokuz Eylul University, Faculty of Sciences, Department of Chemistry, Kaynaklar Campus, 35160 Buca, Izmir, Türkiye

[c]Computational Biology and Molecular Simulations Laboratory, Department of Biophysics, School of Medicine, Bahçeşehir University, Istanbul, Türkiye

[d]Lab for Innovative Drugs (Lab4IND), Computational Drug Design Center (HİTMER), Bahçeşehir University, İstanbul, Türkiye

[e]Department of Medical Biology, School of Medicine, Bahcesehir University, Istanbul, Türkiye.

[f]Department of Neurosurgery, School of Medicine, Bahcesehir University, Istanbul, Türkiye.

[g]Molecular Therapy Lab, Department of Pharmaceutical Chemistry, School of Pharmacy, Bahçeşehir University, Istanbul, Türkiye

FTIR, 1H-NMR, 13C-NMR and HRMS Spectrums of Synthesized Tryptamine-thiazolidin-4-one derivatives (**YS1-12**)

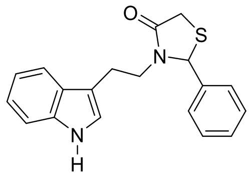

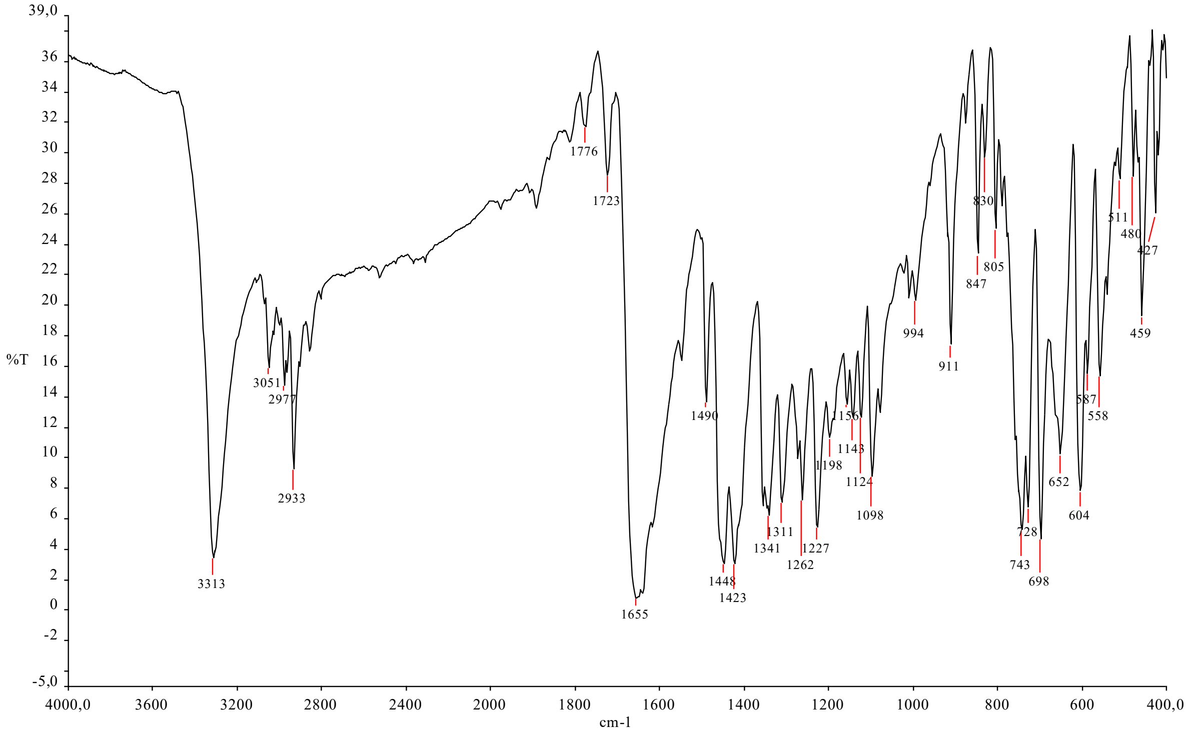

Figure S1: IR Spectrum of Compound **YS1**

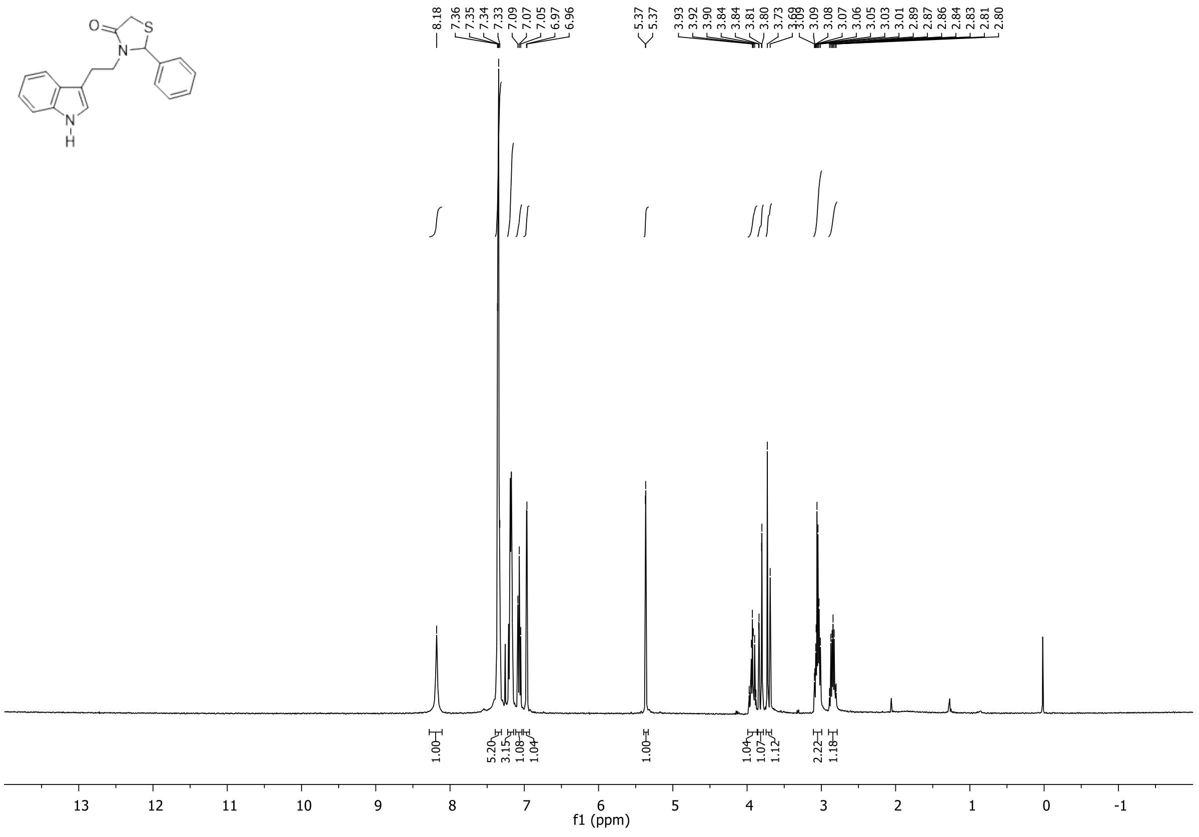

Figure S2: ^{1}H-NMR Spectrum of Compound **YS1**

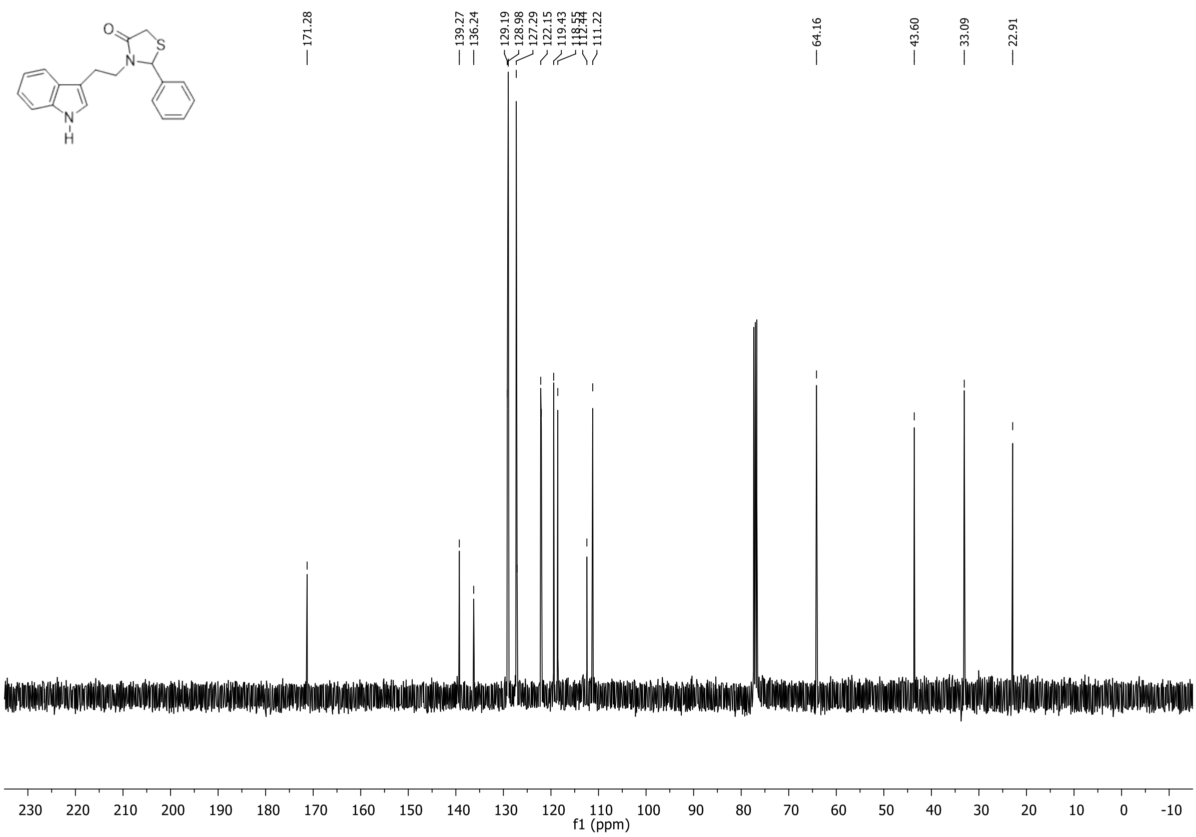

Figure S3: ^{13}C-NMR Spectrum of Compound **YS1**

Elemental Composition Report

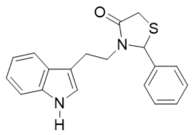

Single Mass Analysis

Tolerance = 10000.0 PPM / DBE: min = -5.5, max = 1000.0
Element prediction: Off
Number of isotope peaks used for i-FIT = 9

Monoisotopic Mass, Odd and Even Electron Ions
3 formula(e) evaluated with 1 results within limits (all results (up to 1000) for each mass)
Elements Used:
C: 19-19 H: 18-19 N: 2-5 O: 1-1 S: 1-1
Seher Aydin
41102_20230926_01-01 4 (0.172) Cm (1:5)

1: TOF MS ES+
1.02e+004

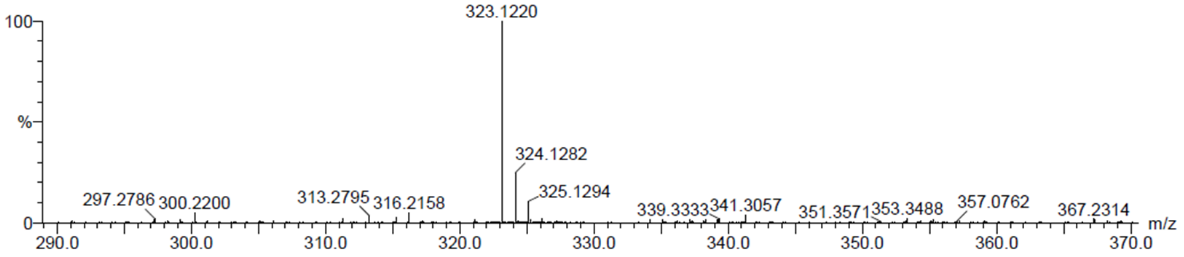

	Mass	Calc. Mass	mDa	PPM	DBE	i-FIT	i-FIT (Norm)	Formula
Minimum:					-5.5			
Maximum:			1000.0	10000.0	1000.0			
	323.1220	323.1218	0.2	0.6	11.5	637.1	0.0	C19 H19 N2 O S

Figure S4: HRMS Spectrums of Compound **YS1**

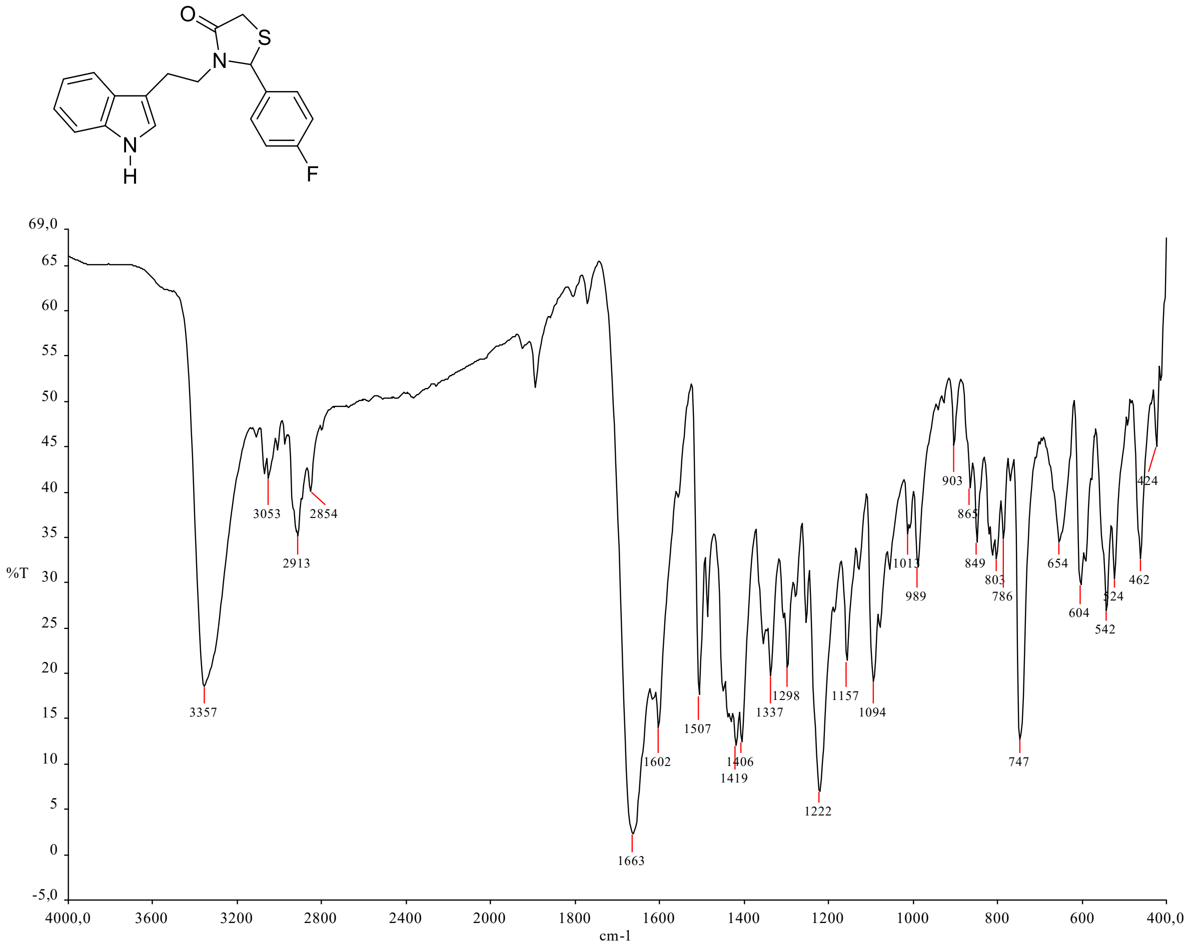

Figure S5: IR Spectrum of Compound **YS2**

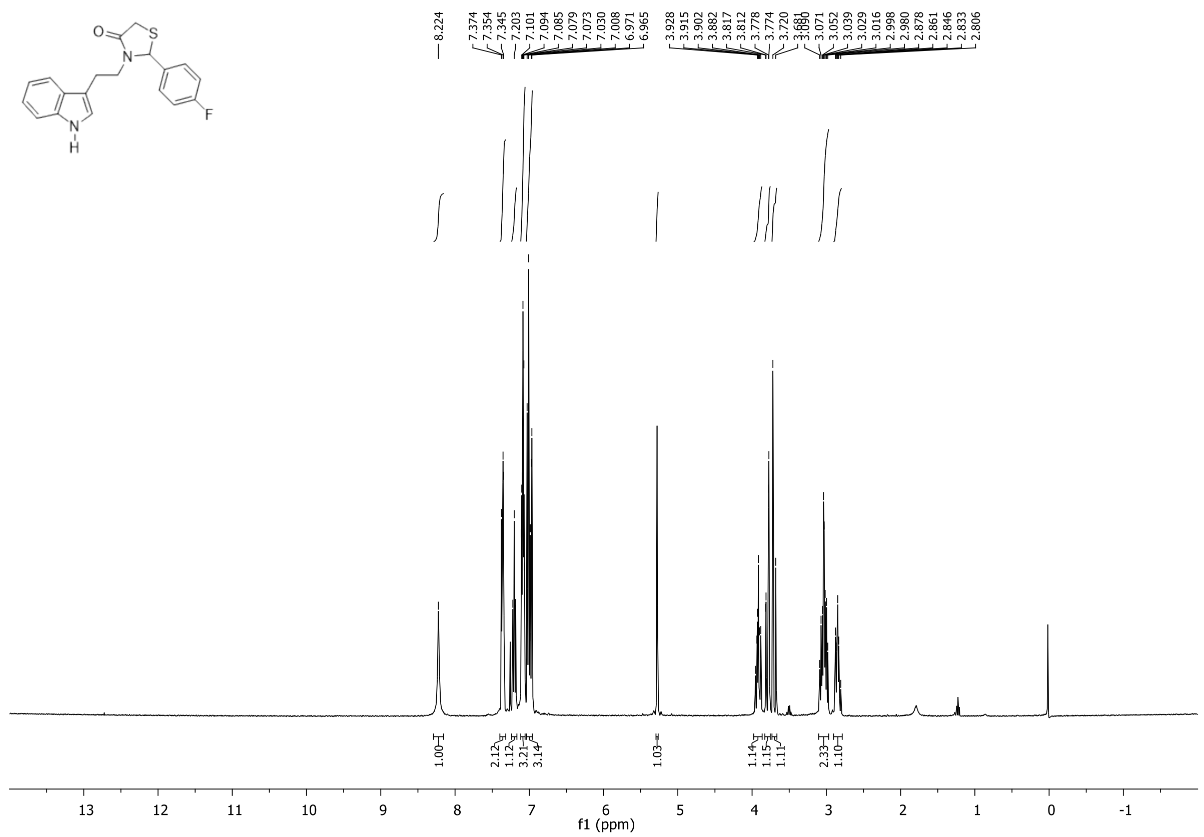

Figure S6: ¹H-NMR Spectrum of Compound **YS2**

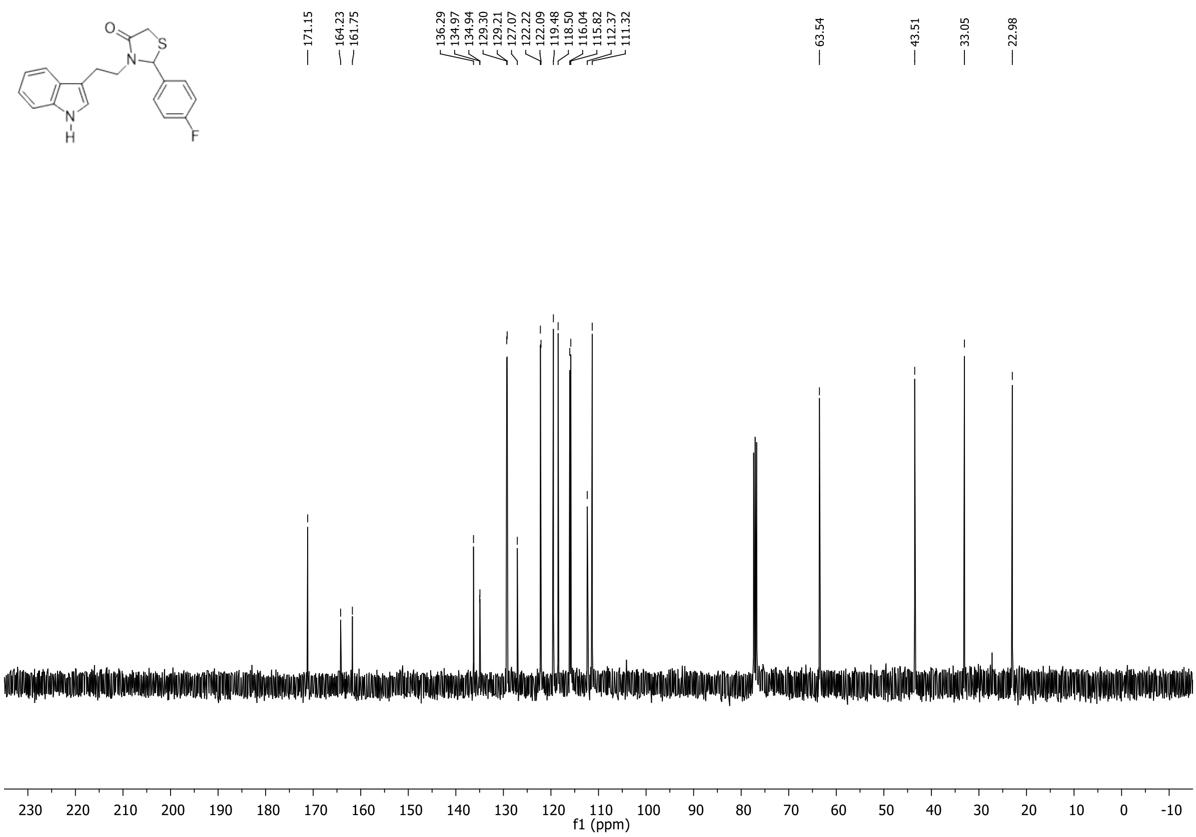

Figure S7: ^{13}C-NMR Spectrum of Compound **YS2**

Elemental Composition Report

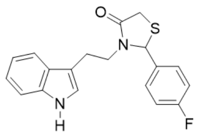

Single Mass Analysis

Tolerance = 10000.0 PPM / DBE: min = -5.5, max = 1000.0
Element prediction: Off
Number of isotope peaks used for i-FIT = 9

Monoisotopic Mass, Odd and Even Electron Ions
2 formula(e) evaluated with 1 results within limits (all results (up to 1000) for each mass)
Elements Used:
C: 19-19 H: 16-21 N: 2-2 O: 1-2 F: 1-2 S: 1-1
Seher Aydin
41102_20230928_02-03 5 (0.206) Cm (5:7)
1: TOF MS ES+
3.53e+006

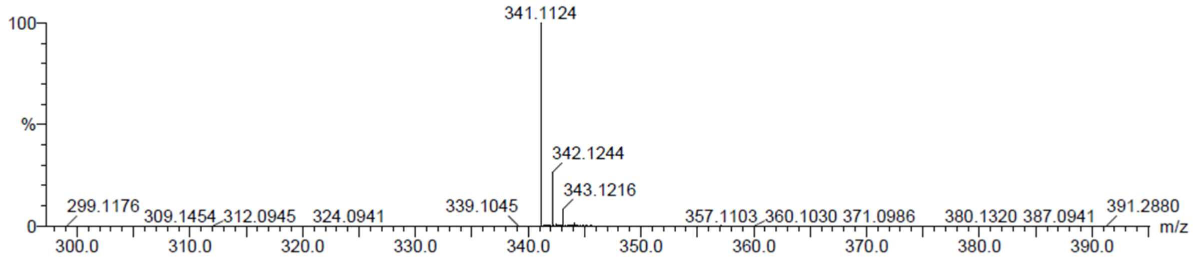

Minimum:				-5.5			
Maximum:		1000.0	10000.0	1000.0			
Mass	Calc. Mass	mDa	PPM	DBE	i-FIT	i-FIT (Norm)	Formula
341.1124	341.1124	0.0	0.0	11.5	1285.2	0.0	C19 H18 N2 O F S

Figure S8: HRMS Spectrums of Compound **YS2**

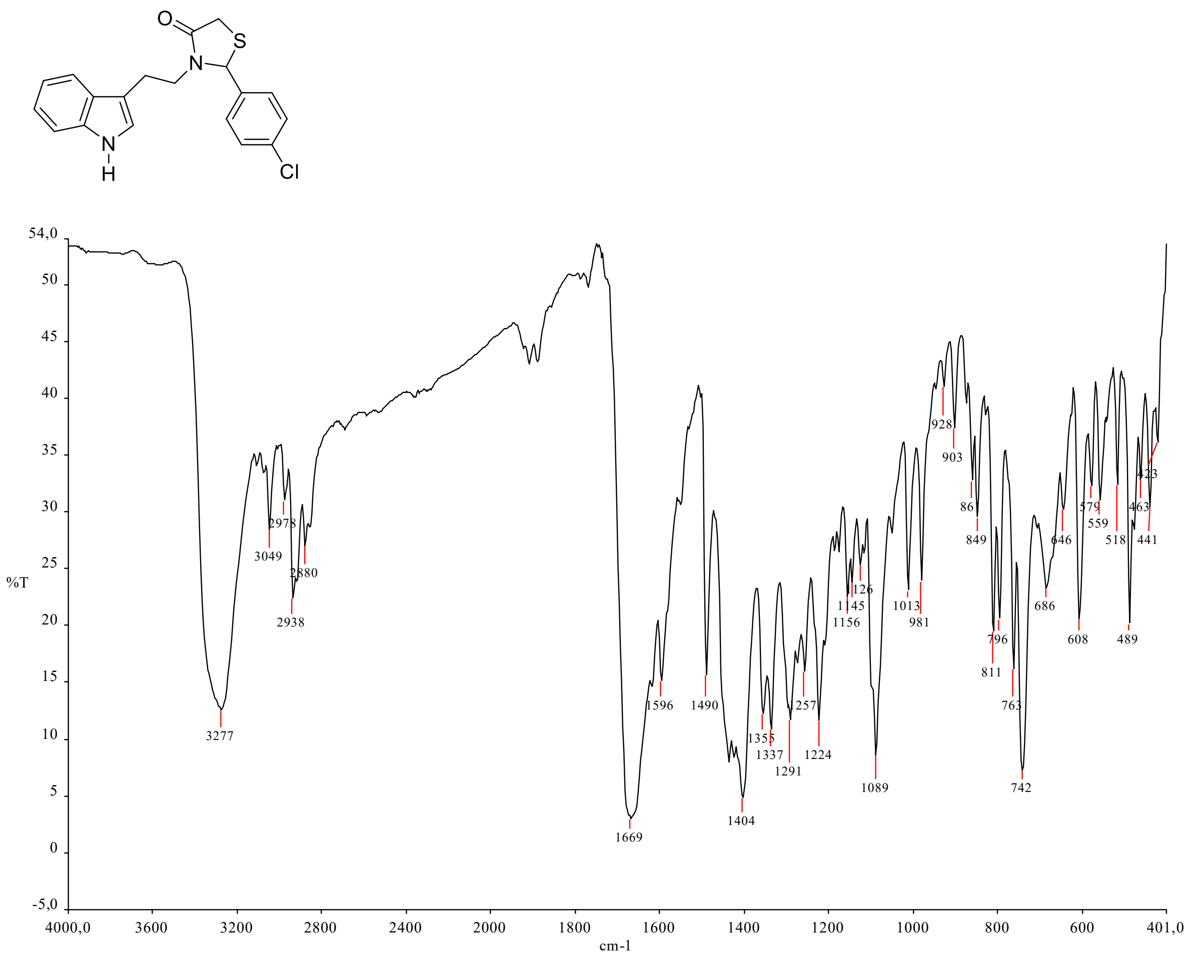

Figure S9: IR Spectrum of Compound **YS3**

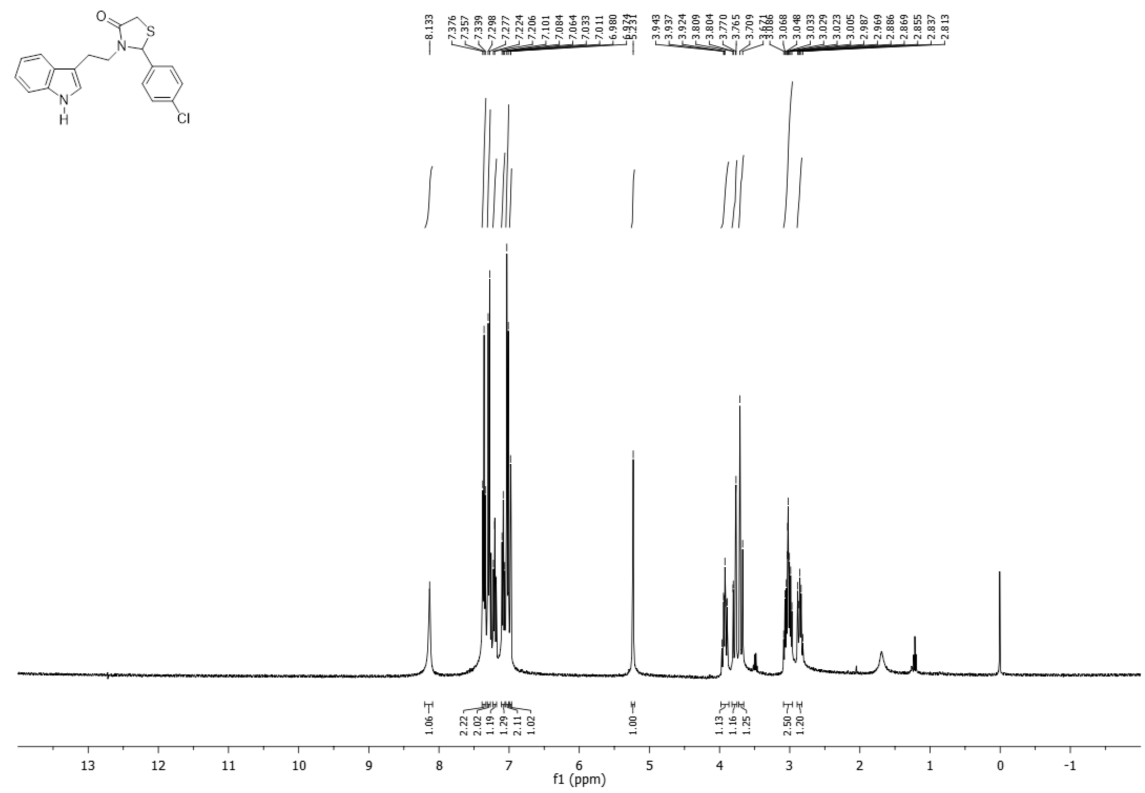

Figure S10: ^{1}H-NMR Spectrum of Compound **YS3**

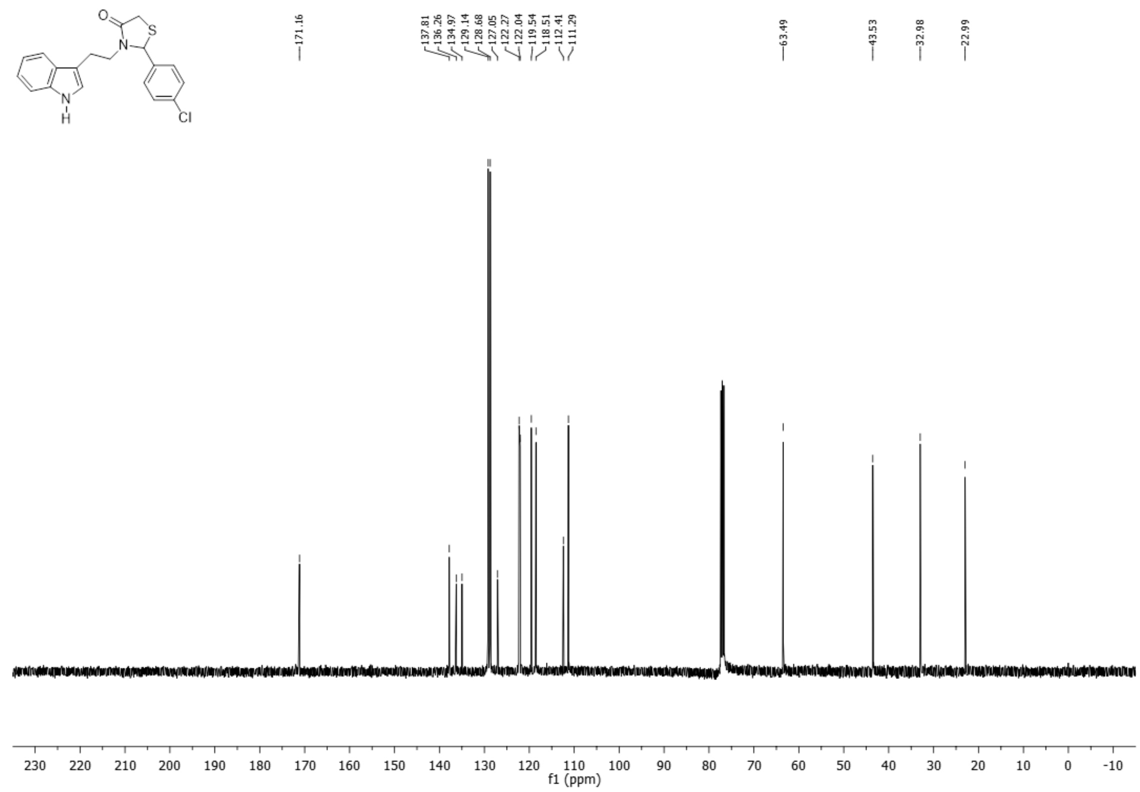

Figure S11: ^{13}C-NMR Spectrum of Compound **YS3**

Elemental Composition Report

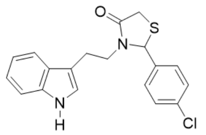

Single Mass Analysis

Tolerance = 10000.0 PPM / DBE: min = -5.5, max = 1000.0
Element prediction: Off
Number of isotope peaks used for i-FIT = 9

Monoisotopic Mass, Even Electron Ions
8 formula(e) evaluated with 1 results within limits (all results (up to 1000) for each mass)
Elements Used:
C: 19-19 H: 16-21 N: 2-2 O: 1-2 S: 1-1 Cl: 1-3 Na: 0-1

Seher Aydin
41102_20230928_03-02 20 (0.775) Cm (18:22)

1: TOF MS ES+
2.46e+004

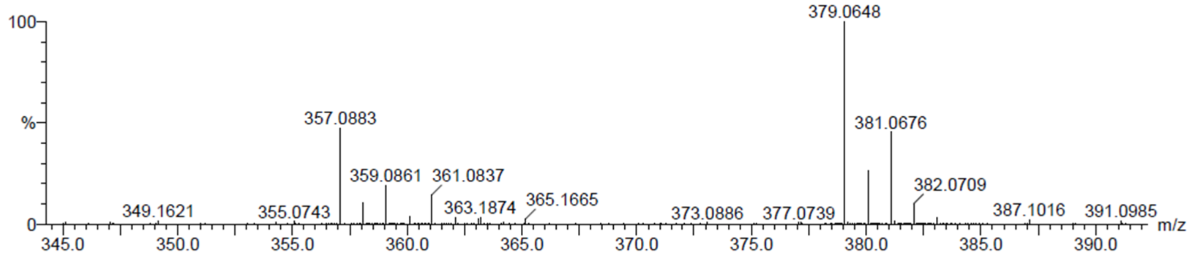

	Mass	Calc. Mass	mDa	PPM	DBE	i-FIT	i-FIT (Norm)	Formula
Minimum:					-5.5			
Maximum:			1000.0	10000.0	1000.0			
	379.0648	379.0648	0.0	0.0	11.5	783.0	0.0	C19 H17 N2 O S Cl

Figure S12: HRMS Spectrums of Compound **YS3**

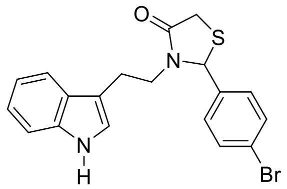

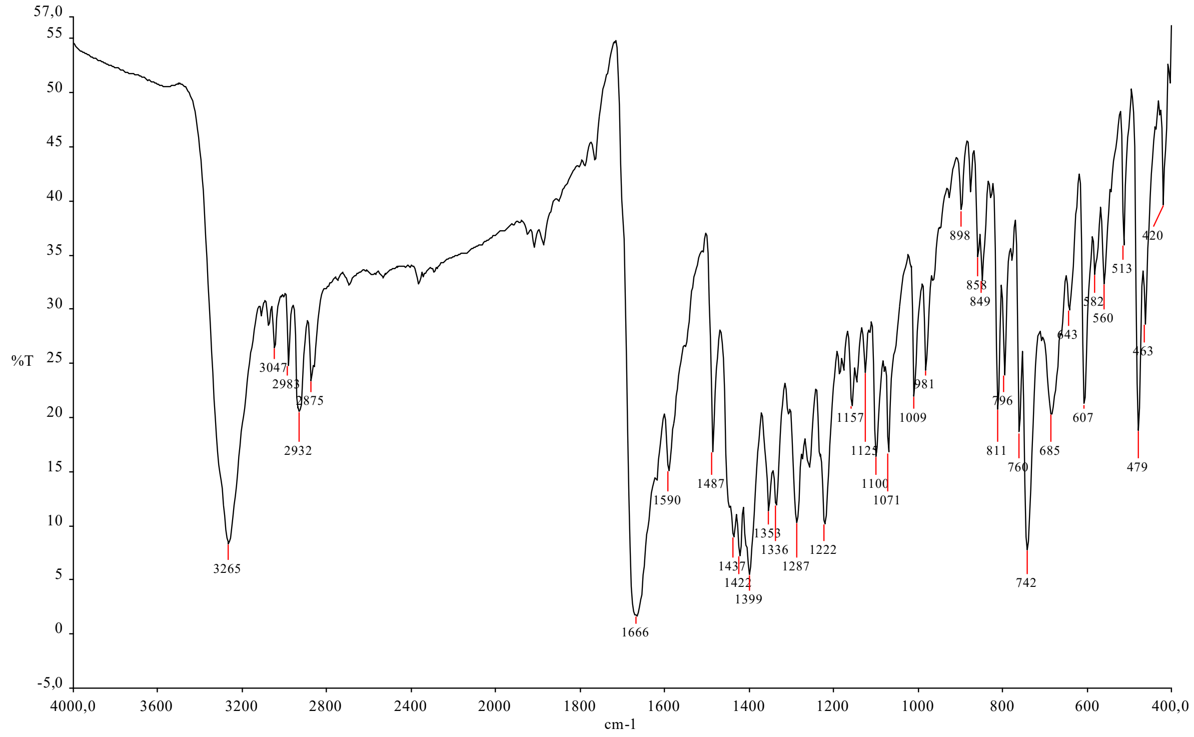

Figure S13: IR Spectrum of Compound **YS4**

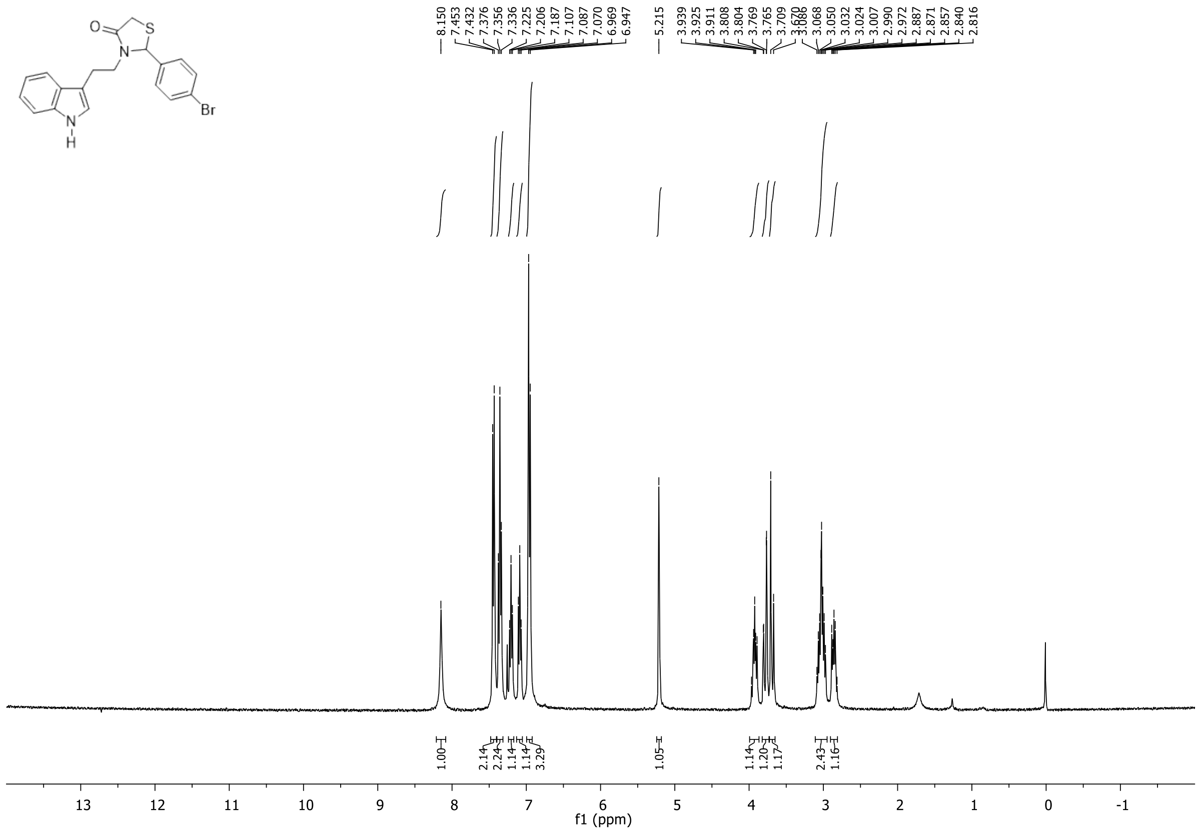

Figure S14: ^{1}H-NMR Spectrum of Compound **YS4**

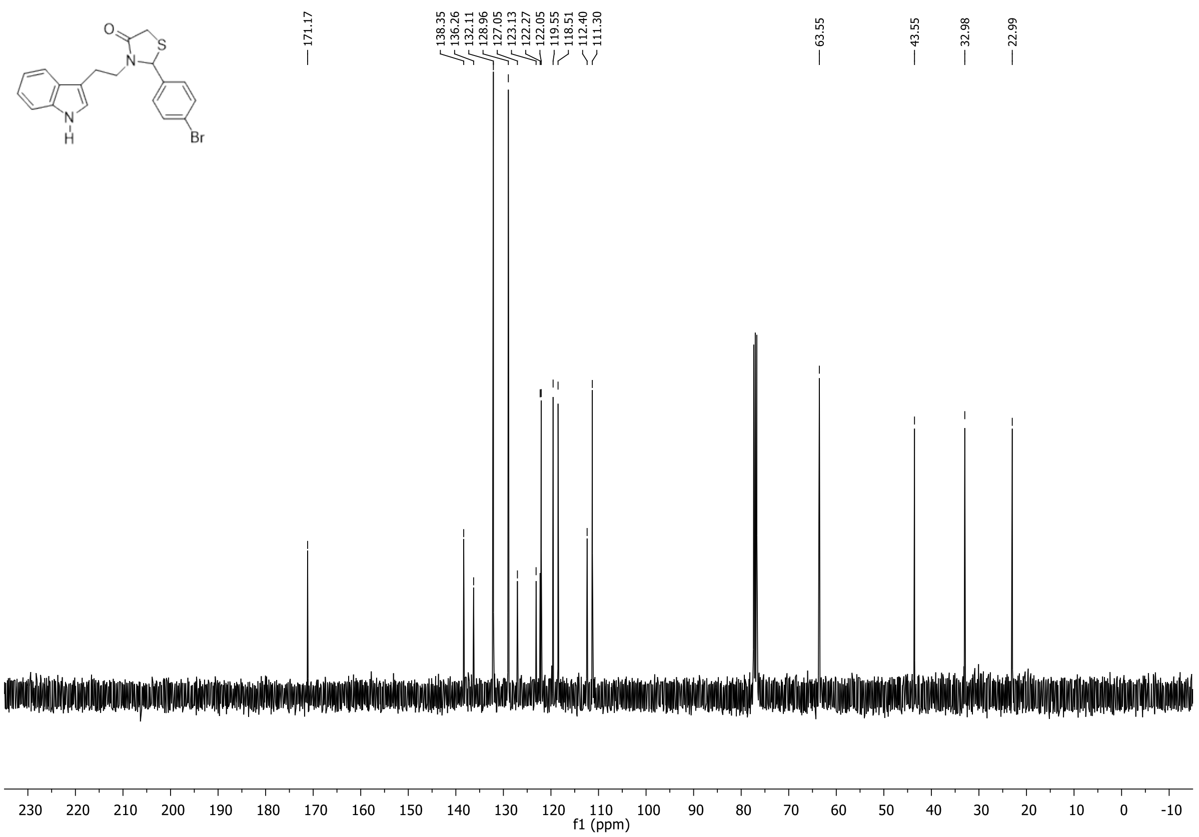

Figure S15: ^{13}C-NMR Spectrum of Compound **YS4**

Elemental Composition Report

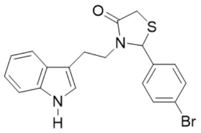

Single Mass Analysis

Tolerance = 10000.0 PPM / DBE: min = -5.5, max = 1000.0
Element prediction: Off
Number of isotope peaks used for i-FIT = 9

Monoisotopic Mass, Even Electron Ions
14 formula(e) evaluated with 1 results within limits (all results (up to 1000) for each mass)
Elements Used:
C: 19-19 H: 16-17 N: 2-2 O: 1-2 S: 1-1 79Br: 0-2 81Br: 0-2

Seher Aydin
41102_20230928_04-N03 1 (0.070) Cm (1:4)

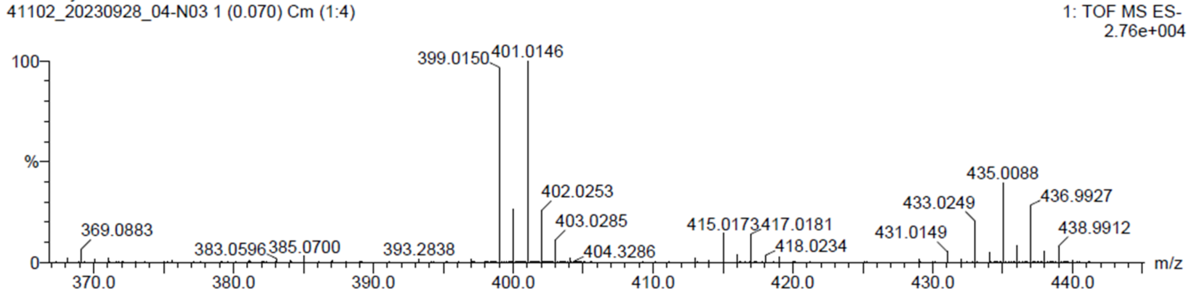

	Calc. Mass	mDa	PPM	DBE	i-FIT	i-FIT (Norm)	Formula
Minimum:				-5.5			
Maximum:		1000.0	10000.0	1000.0			
Mass	Calc. Mass	mDa	PPM	DBE	i-FIT	i-FIT (Norm)	Formula
401.0146	401.0146	0.0	0.0	12.5	727.7	0.0	C19 H16 N2 O S 81Br

Figure S16: HRMS Spectrums of Compound **YS4**

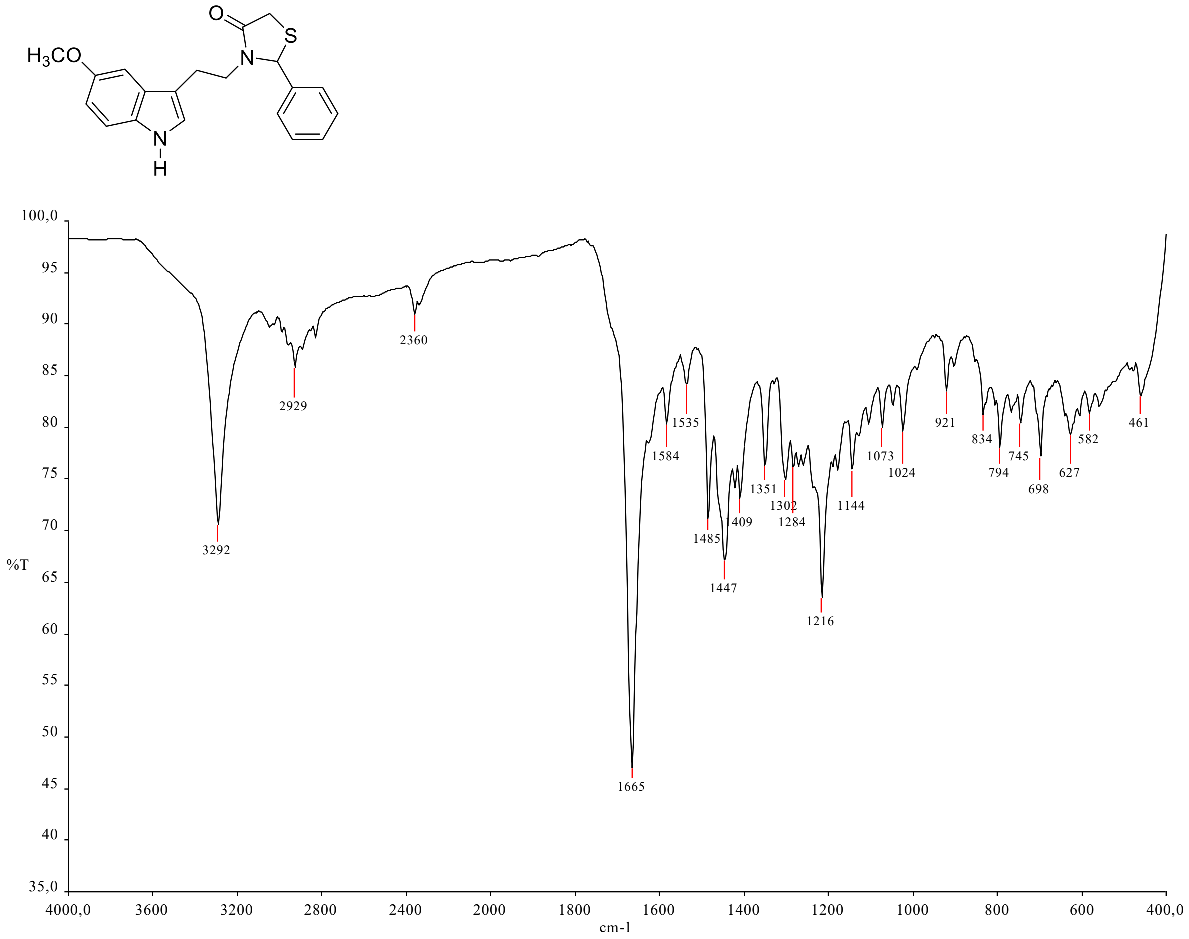

Figure S17: IR Spectrum of Compound **YS5**

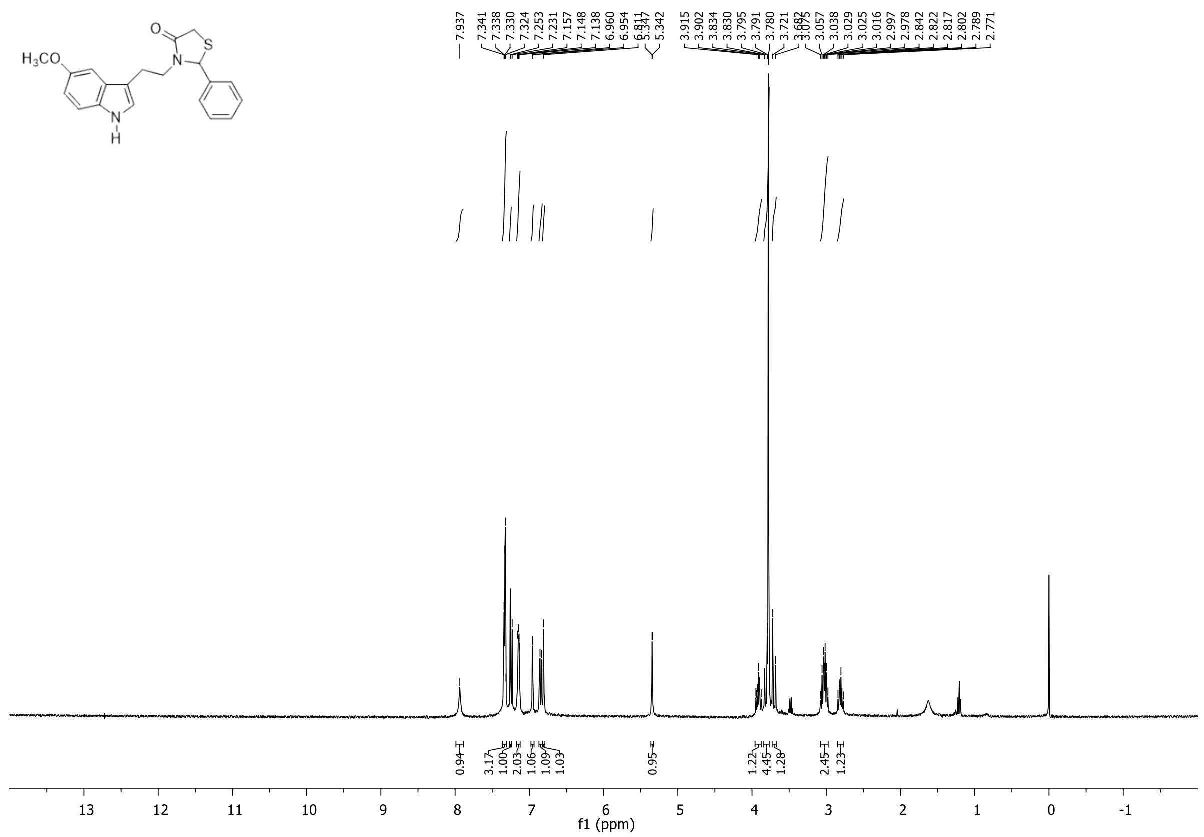

Figure S18: ^{1}H-NMR Spectrum of Compound **YS5**

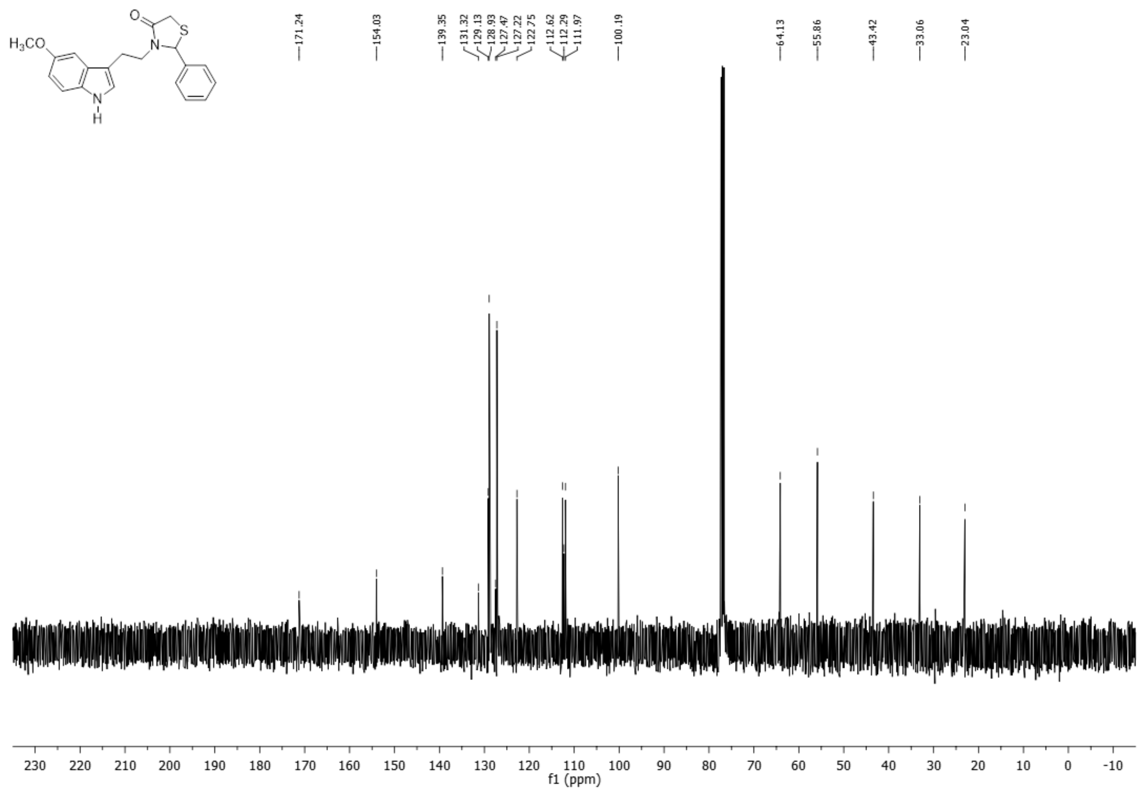

Figure S19: ^{13}C-NMR Spectrum of Compound **YS5**

Elemental Composition Report

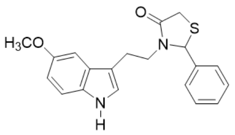

Single Mass Analysis
Tolerance = 10000.0 PPM / DBE: min = -5.5, max = 1000.0
Element prediction: Off
Number of isotope peaks used for i-FIT = 9

Monoisotopic Mass, Odd and Even Electron Ions
1 formula(e) evaluated with 1 results within limits (all results (up to 1000) for each mass)
Elements Used:
C: 20-20 H: 16-21 N: 2-2 O: 1-2 S: 1-1
Seher Aydin
41102_20230928_05-N01 18 (0.707) Cm (15:22)

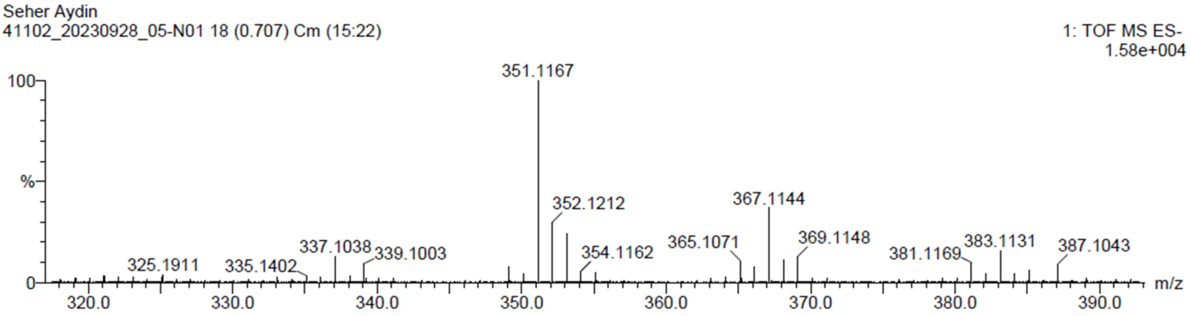

Minimum:				-5.5			
Maximum:		1000.0	10000.0	1000.0			
Mass	Calc. Mass	mDa	PPM	DBE	i-FIT	i-FIT (Norm)	Formula
351.1167	351.1167	0.0	0.0	12.5	920.4	0.0	C20 H19 N2 O2 S

Figure S20: HRMS Spectrums of Compound **YS5**

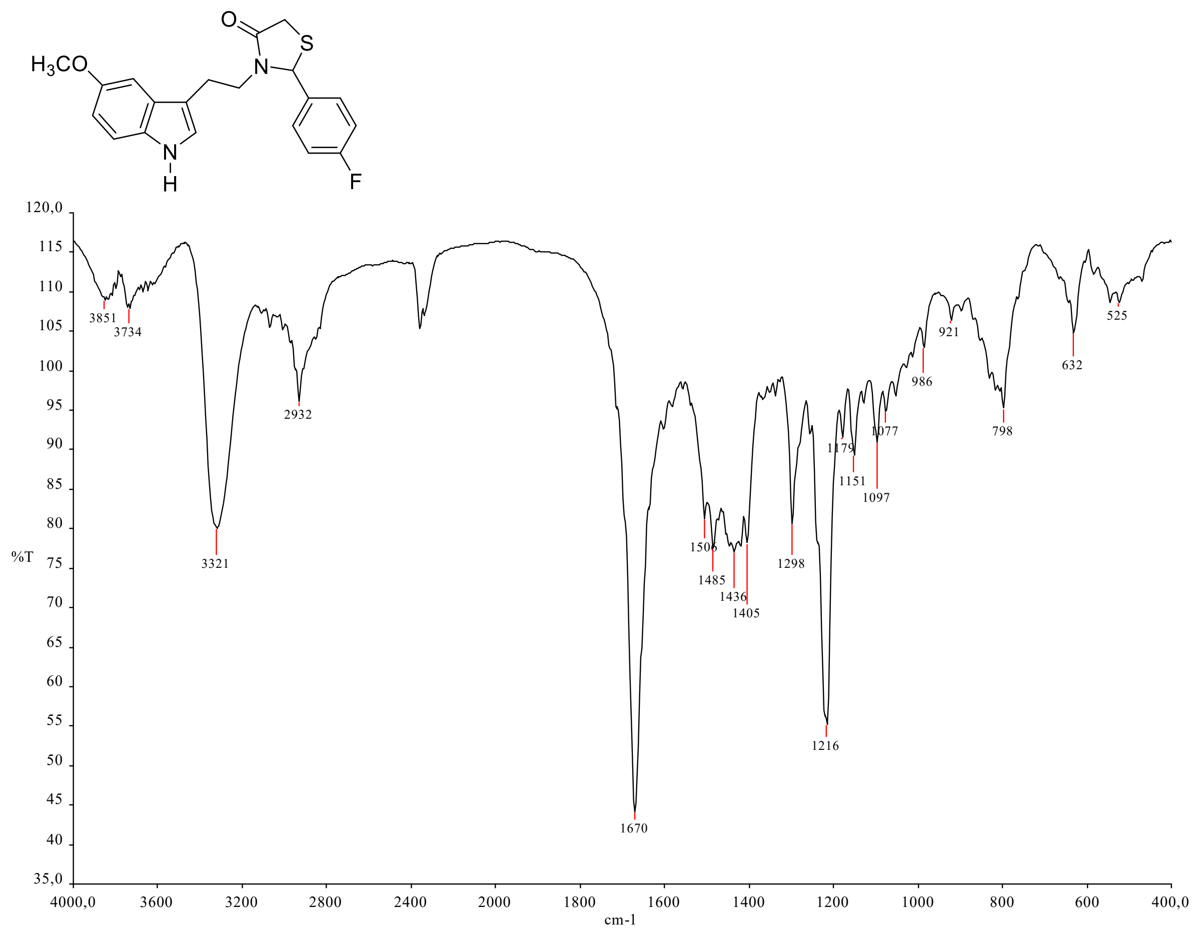

Figure S21: IR Spectrum of Compound **YS6**

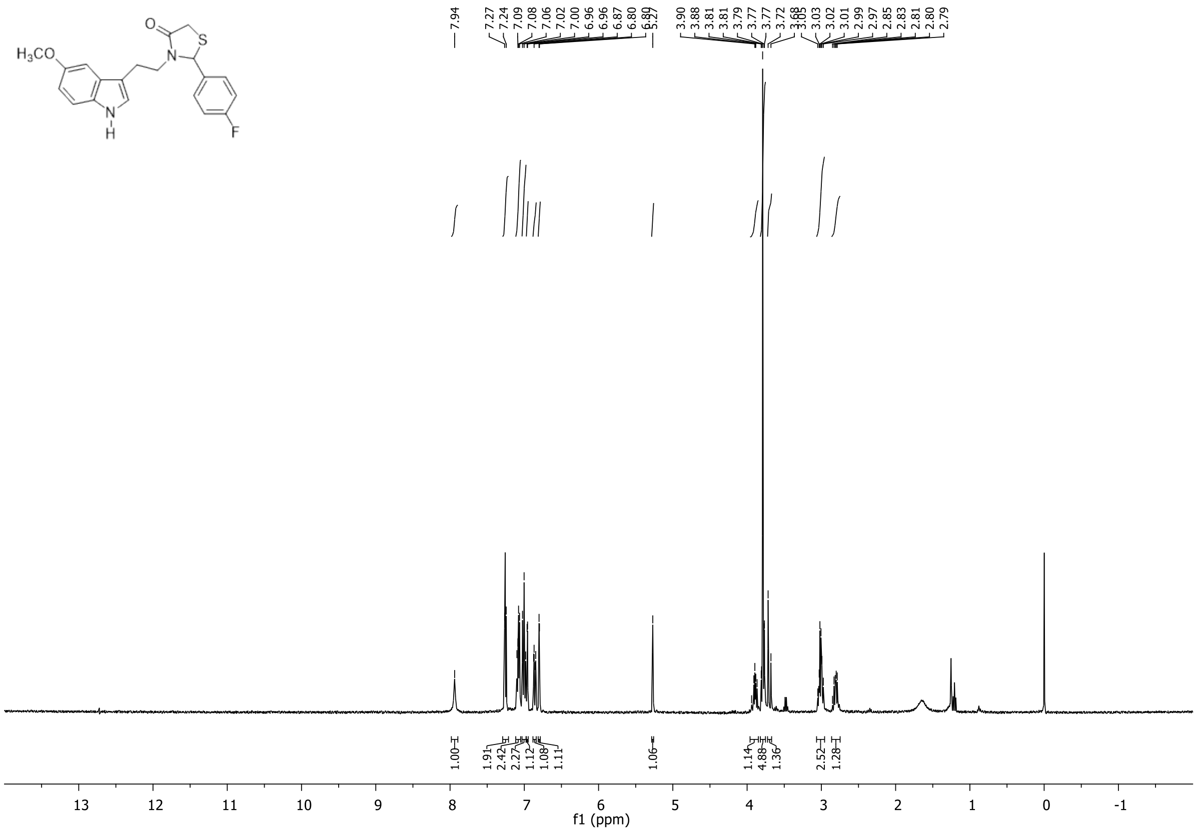

Figure S22: ^{1}H-NMR Spectrum of Compound **YS6**

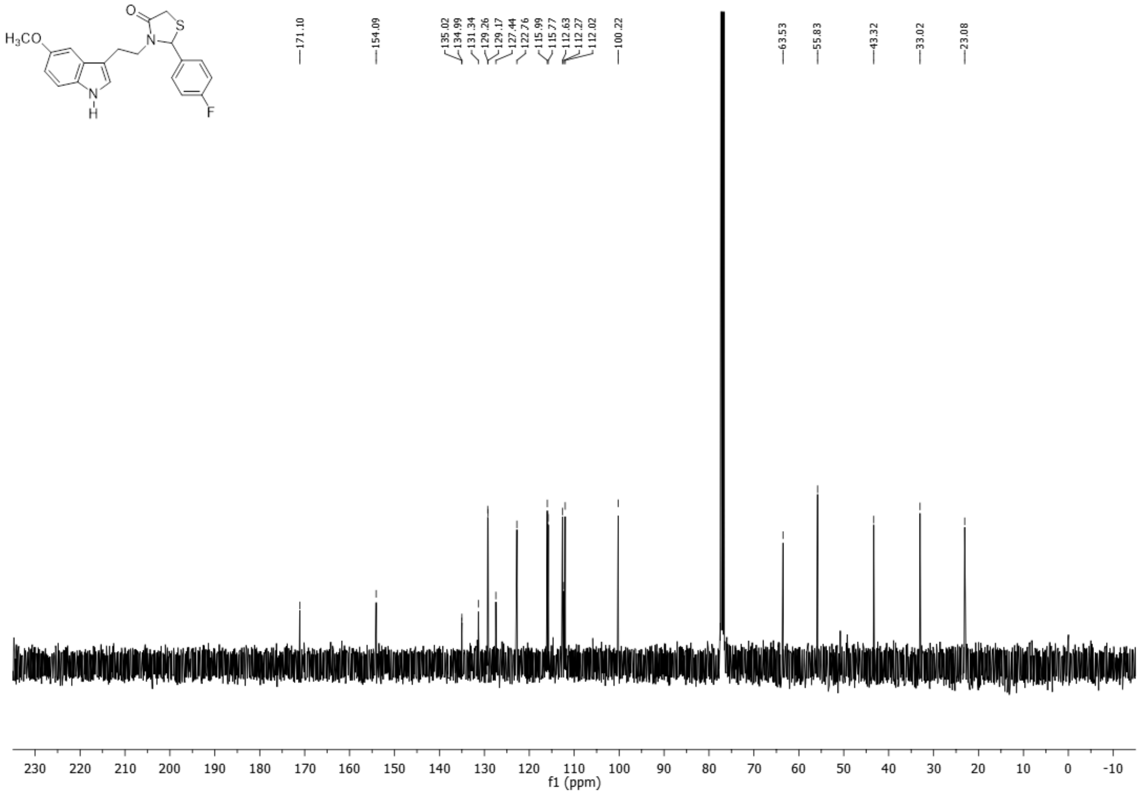

Figure S23: ^{13}C-NMR Spectrum of Compound **YS6**

Elemental Composition Report

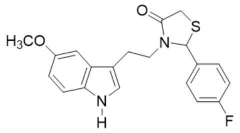

Single Mass Analysis

Tolerance = 10000.0 PPM / DBE: min = -5.5, max = 1000.0
Element prediction: Off
Number of isotope peaks used for i-FIT = 9

Monoisotopic Mass, Odd and Even Electron Ions
6 formula(e) evaluated with 1 results within limits (all results (up to 1000) for each mass)
Elements Used:
C: 20-20 H: 16-21 N: 2-5 O: 2-2 F: 1-2 S: 1-1
Seher Aydin
41102_20230928_06-01 12 (0.484) Cm (8:13)

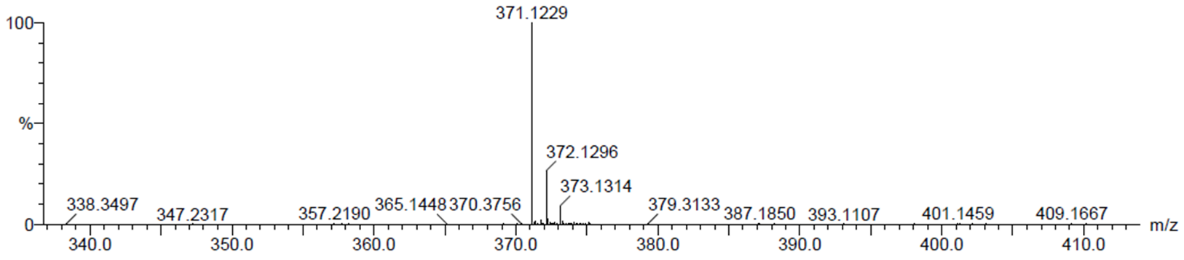

Minimum:				-5.5			
Maximum:		1000.0	10000.0	1000.0			
Mass	Calc. Mass	mDa	PPM	DBE	i-FIT	i-FIT (Norm)	Formula
371.1229	371.1230	-0.1	-0.3	11.5	1138.0	0.0	C20 H20 N2 O2 F S

Figure S24: HRMS Spectrums of Compound **YS6**

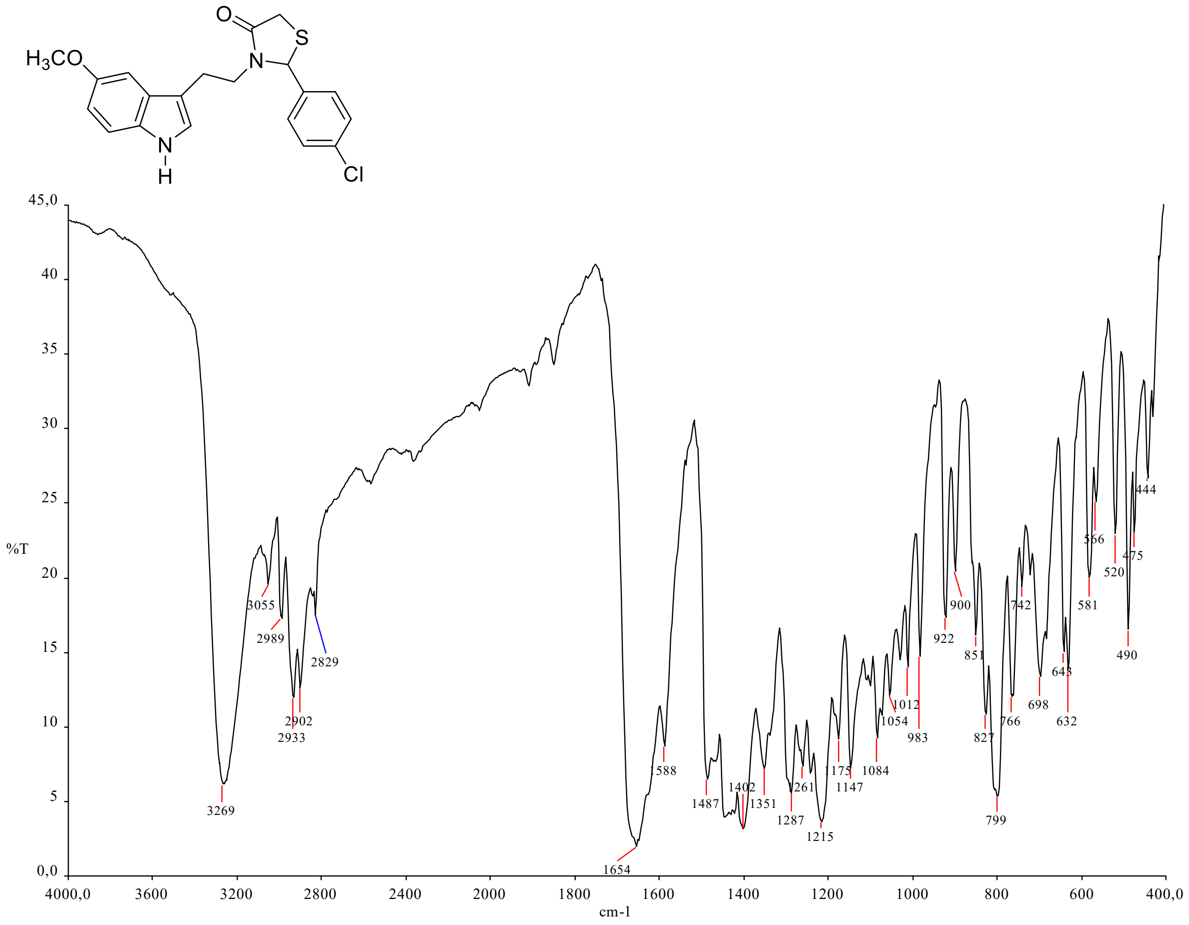

Figure S25: IR Spectrum of Compound **YS7**

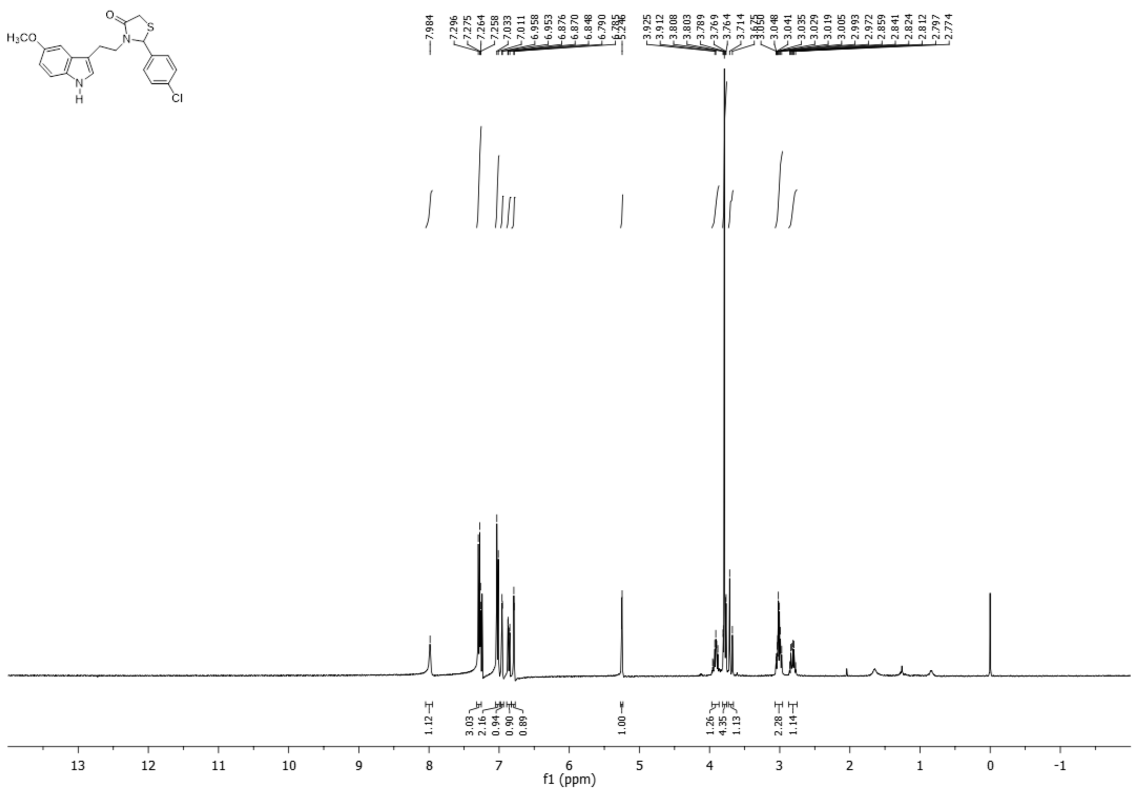

Figure S26: ^{1}H-NMR Spectrum of Compound **YS7**

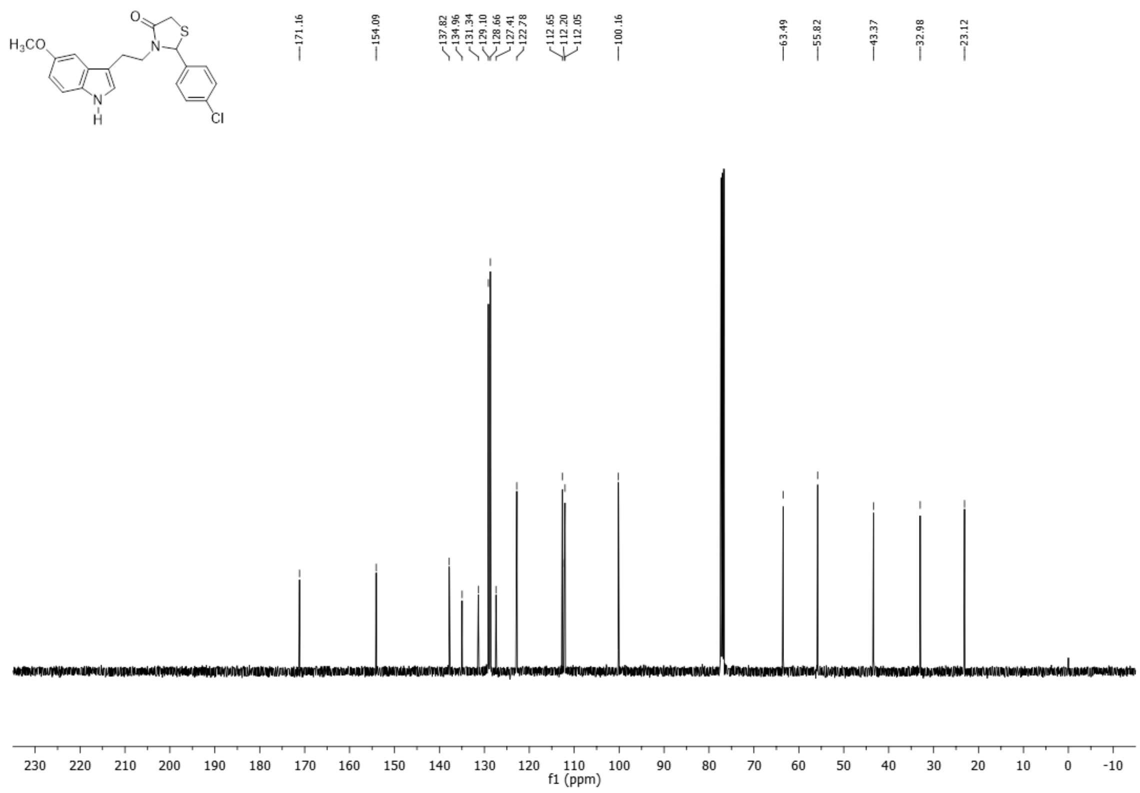

Figure S27: ^{13}C-NMR Spectrum of Compound **YS7**

Elemental Composition Report

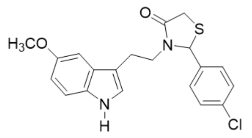

Single Mass Analysis

Tolerance = 10000.0 PPM / DBE: min = -5.5, max = 1000.0
Element prediction: Off
Number of isotope peaks used for i-FIT = 9

Monoisotopic Mass, Even Electron Ions
11 formula(e) evaluated with 1 results within limits (all results (up to 1000) for each mass)
Elements Used:
C: 20-20 H: 16-21 N: 2-5 O: 2-2 S: 1-1 Cl: 1-3
Seher Aydin
41102_20230928_07-02 4 (0.172) Cm (3:9)
1: TOF MS ES+
3.41e+004

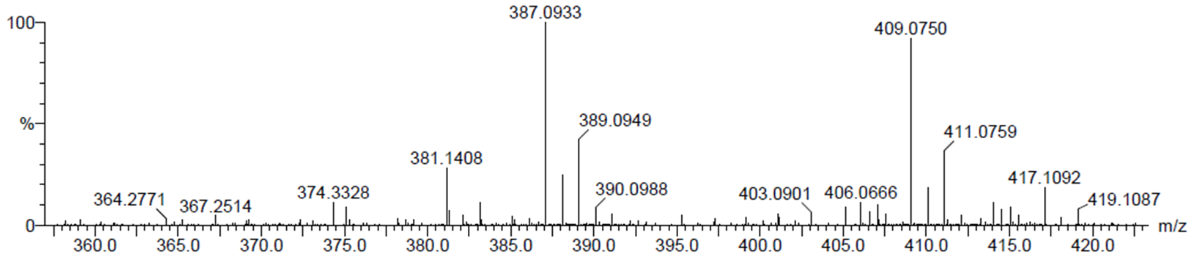

Minimum:				-5.5			
Maximum:		1000.0	10000.0	1000.0			
Mass	Calc. Mass	mDa	PPM	DBE	i-FIT	i-FIT (Norm)	Formula
387.0933	387.0934	-0.1	-0.3	11.5	848.5	0.0	C20 H20 N2 O2 S Cl

Figure S28: HRMS Spectrums of Compound **YS7**

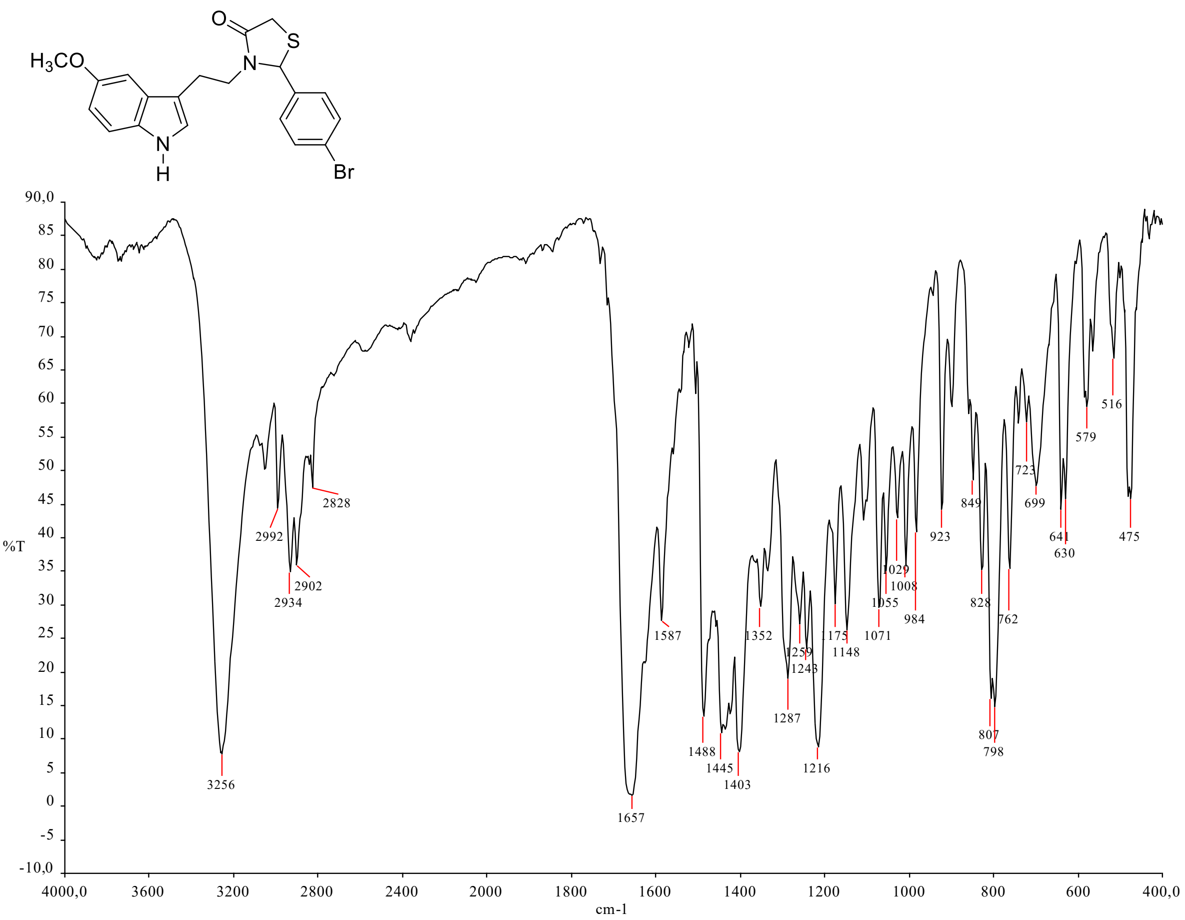

Figure S29: IR Spectrum of Compound **YS8**

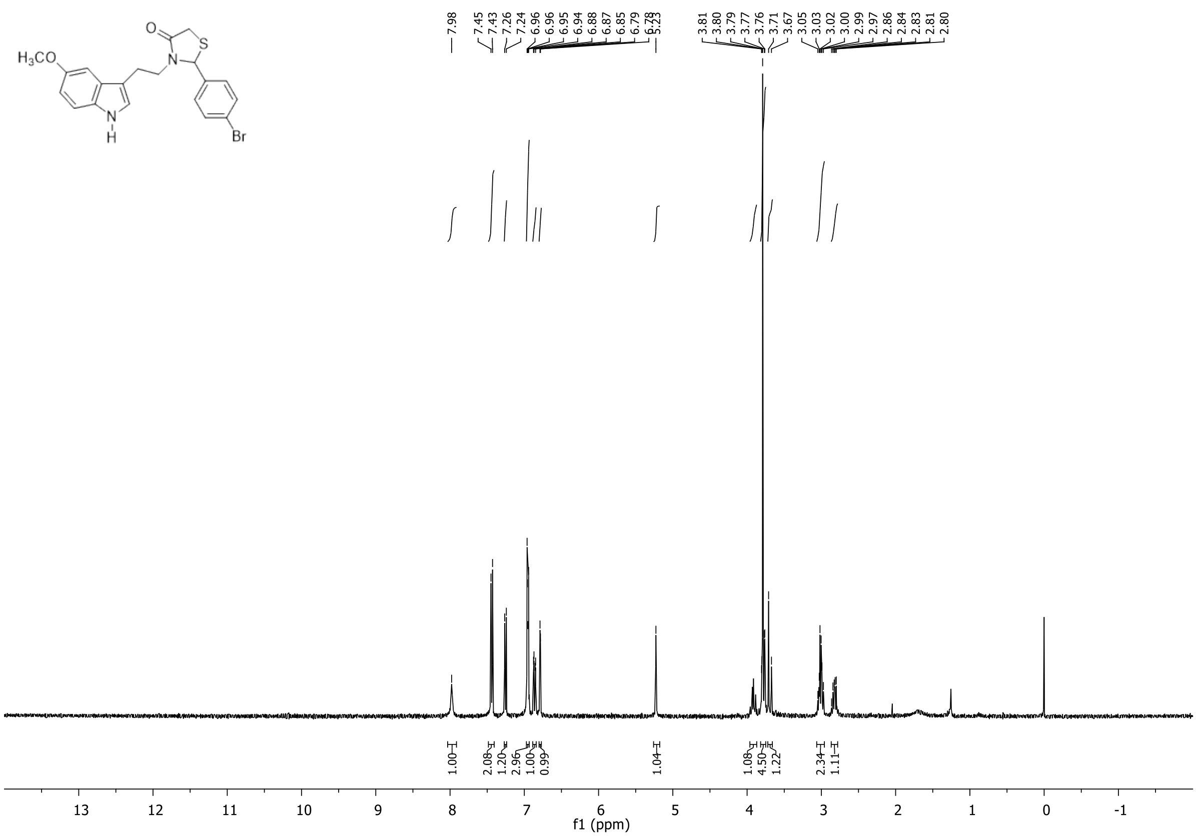

Figure S30: ^{1}H-NMR Spectrum of Compound **YS8**

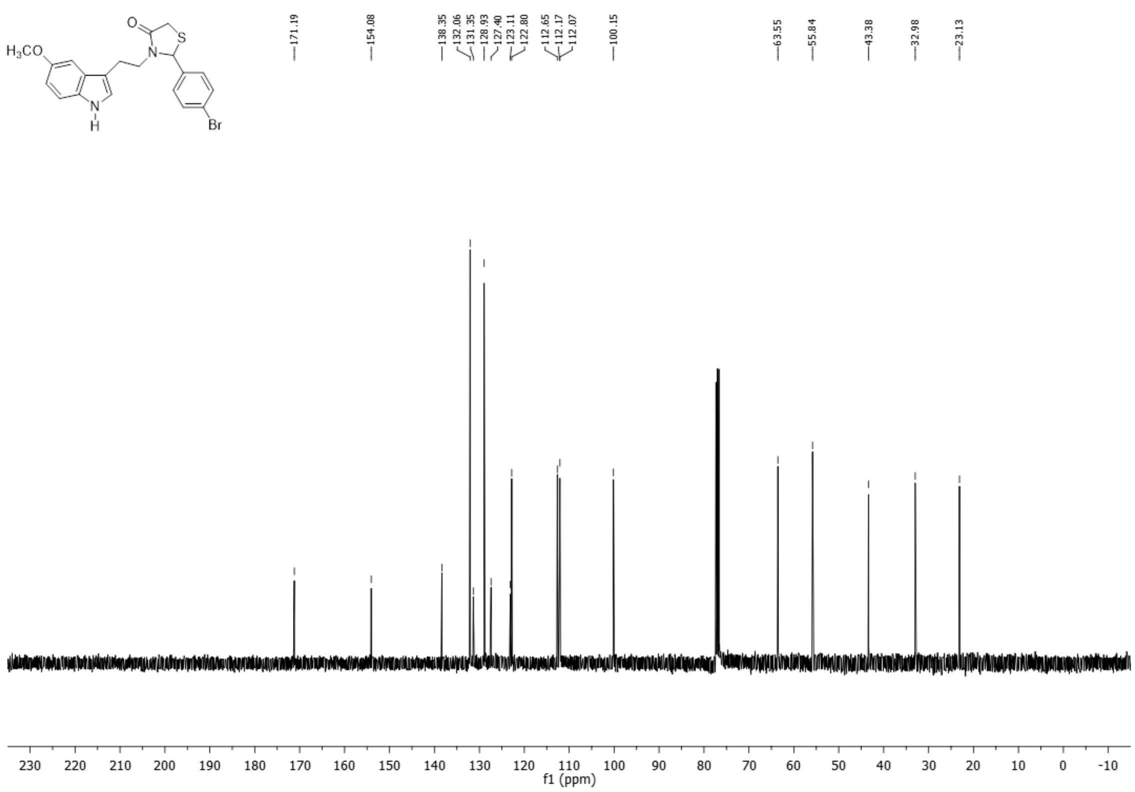

Figure S31: ^{13}C-NMR Spectrum of Compound **YS8**

Elemental Composition Report

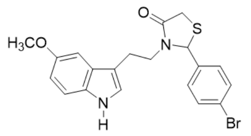

Single Mass Analysis

Tolerance = 10000.0 PPM / DBE: min = -5.5, max = 1000.0
Element prediction: Off
Number of isotope peaks used for i-FIT = 9

Monoisotopic Mass, Odd and Even Electron Ions
29 formula(e) evaluated with 1 results within limits (all results (up to 1000) for each mass)
Elements Used:
C: 20-20 H: 16-21 N: 2-5 O: 2-2 S: 1-1 79Br: 0-2 81Br: 0-2

Seher Aydin
41102_20230928_08-01 13 (0.518) Cm (10:13)

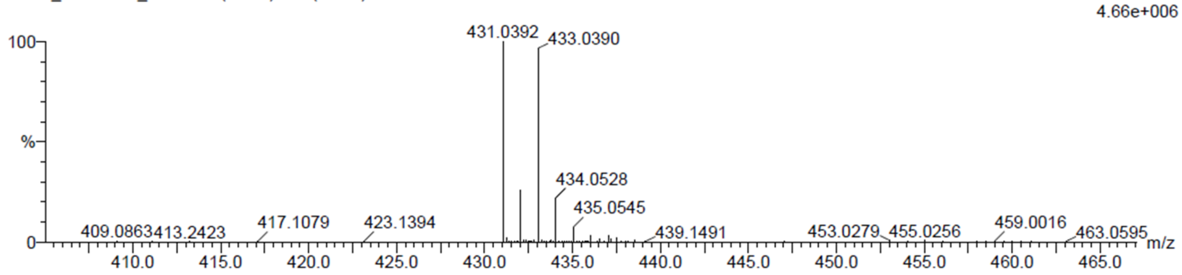

Minimum:				-5.5			
Maximum:		1000.0	10000.0	1000.0			
Mass	Calc. Mass	mDa	PPM	DBE	i-FIT	i-FIT (Norm)	Formula
433.0390	433.0408	-1.8	-4.2	11.5	1204.4	0.0	C20 H20 N2 O2 S 81Br

Figure S32: HRMS Spectrums of Compound **YS8**

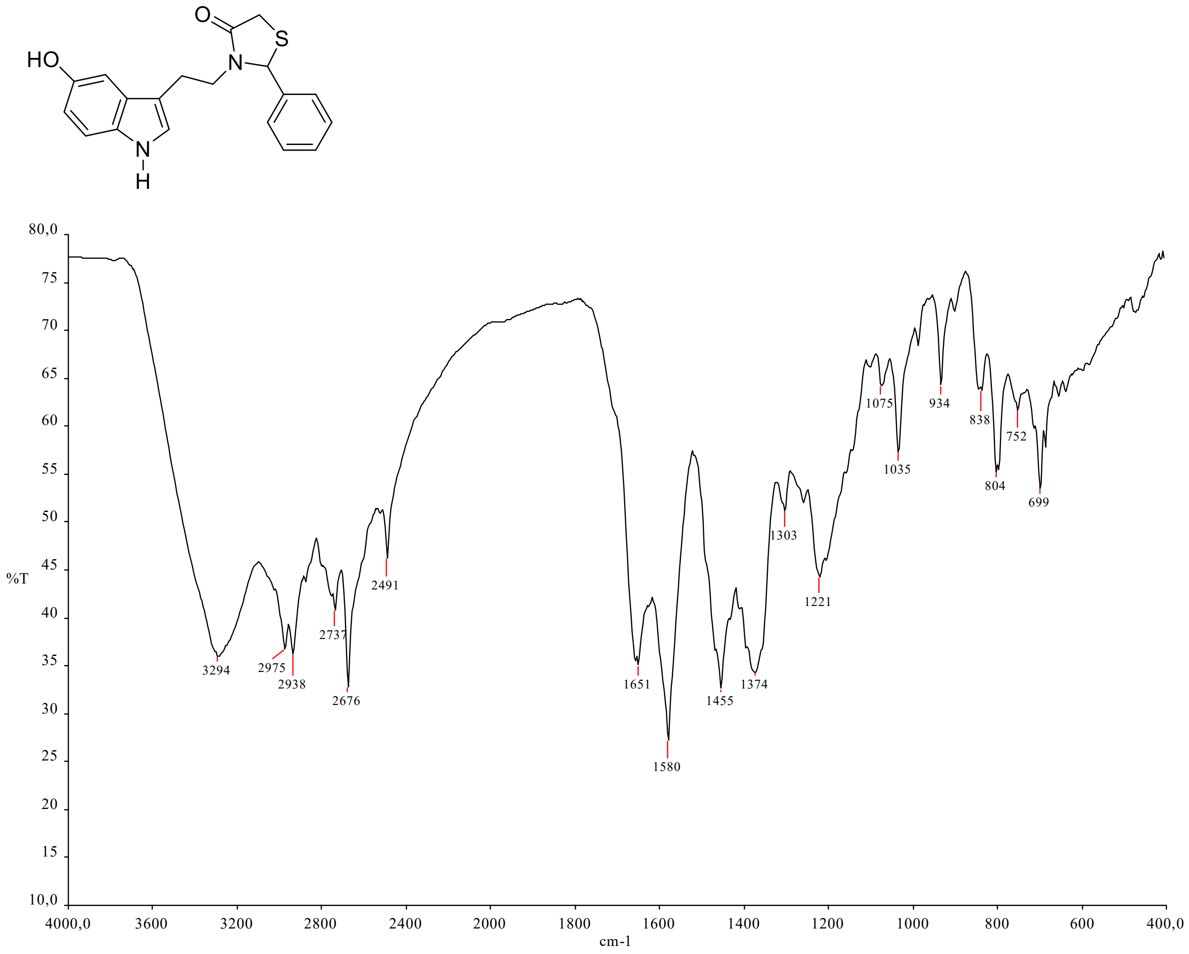

Figure S33: IR Spectrum of Compound **YS9**

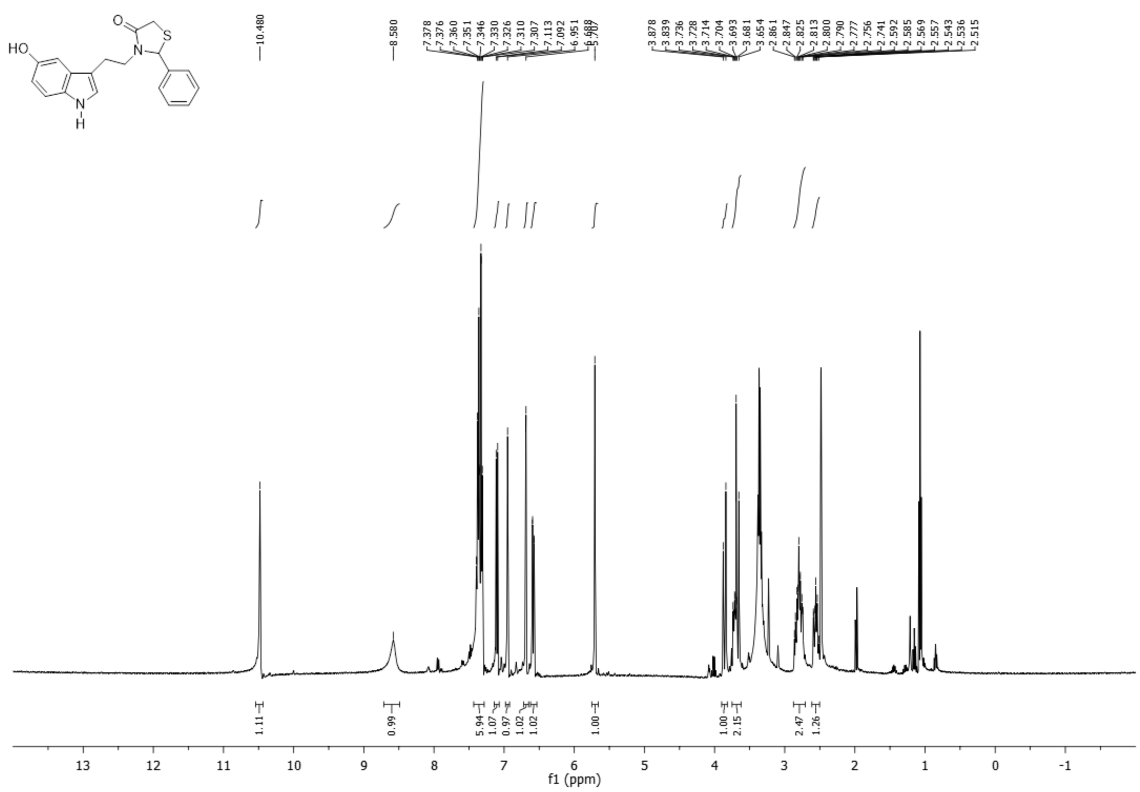

Figure S34: ^{1}H-NMR Spectrum of Compound **YS9**

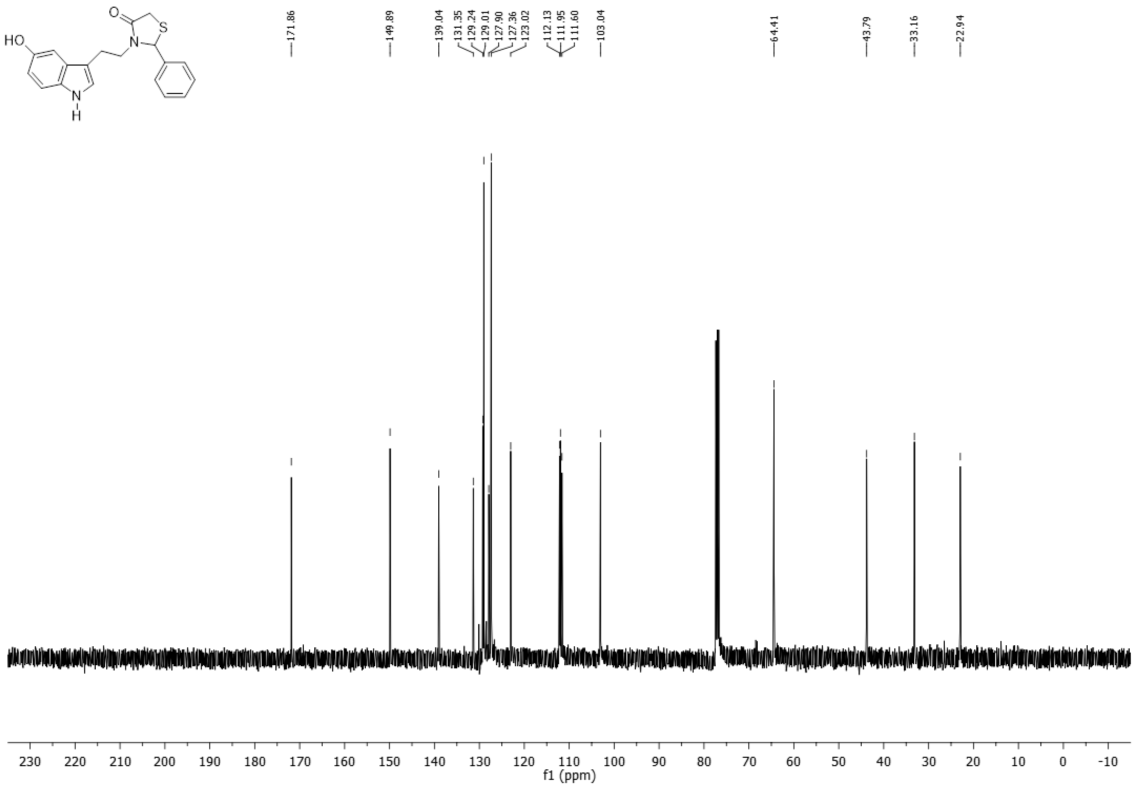

Figure S35: ^{13}C-NMR Spectrum of Compound **YS9**

Elemental Composition Report

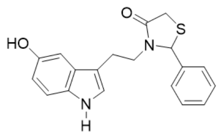

Single Mass Analysis
Tolerance = 10000.0 PPM / DBE: min = -5.5, max = 1000.0
Element prediction: Off
Number of isotope peaks used for i-FIT = 9

Monoisotopic Mass, Odd and Even Electron Ions
4 formula(e) evaluated with 1 results within limits (all results (up to 1000) for each mass)
Elements Used:
C: 19-19 H: 17-19 N: 2-5 O: 1-2 S: 1-1
Seher Aydin
41102_20230926_09-01 1 (0.070) Cm (1:11)

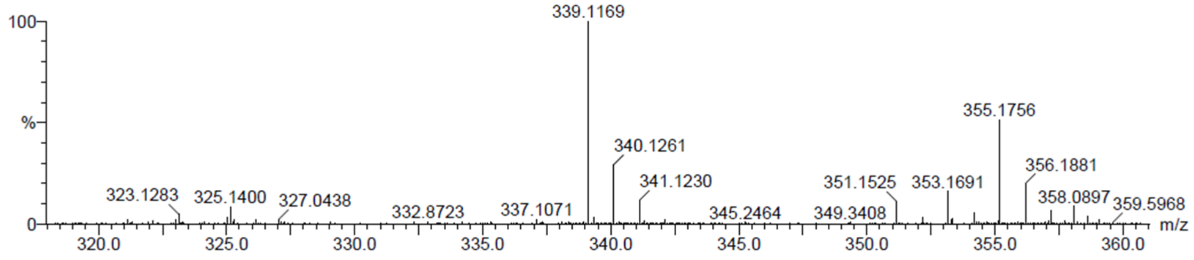

Minimum:				-5.5			
Maximum:		1000.0	10000.0	1000.0			
Mass	Calc. Mass	mDa	PPM	DBE	i-FIT	i-FIT (Norm)	Formula
339.1169	339.1167	0.2	0.6	11.5	802.3	0.0	C19 H19 N2 O2 S

Figure S36: HRMS Spectrums of Compound **YS9**

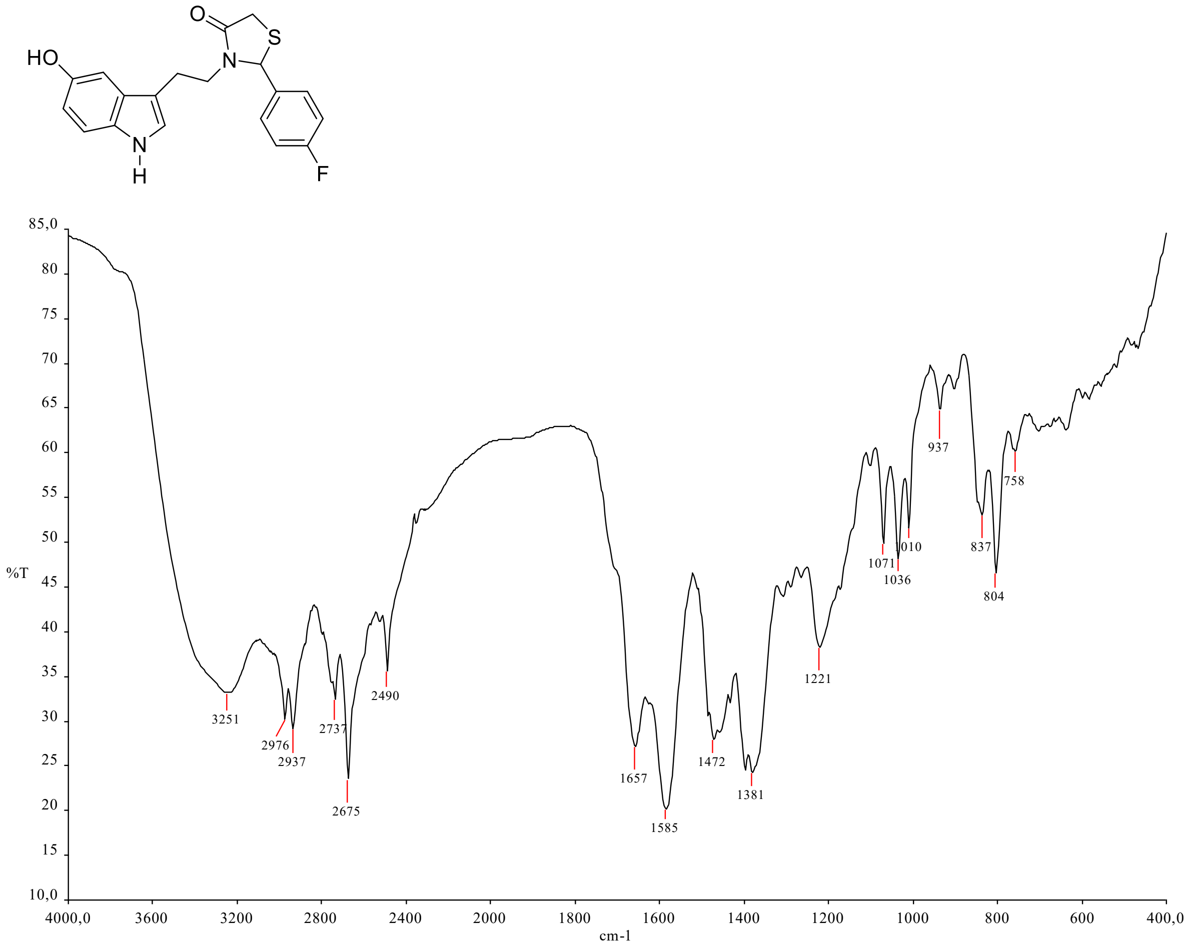

Figure S37: IR Spectrum of Compound **YS10**

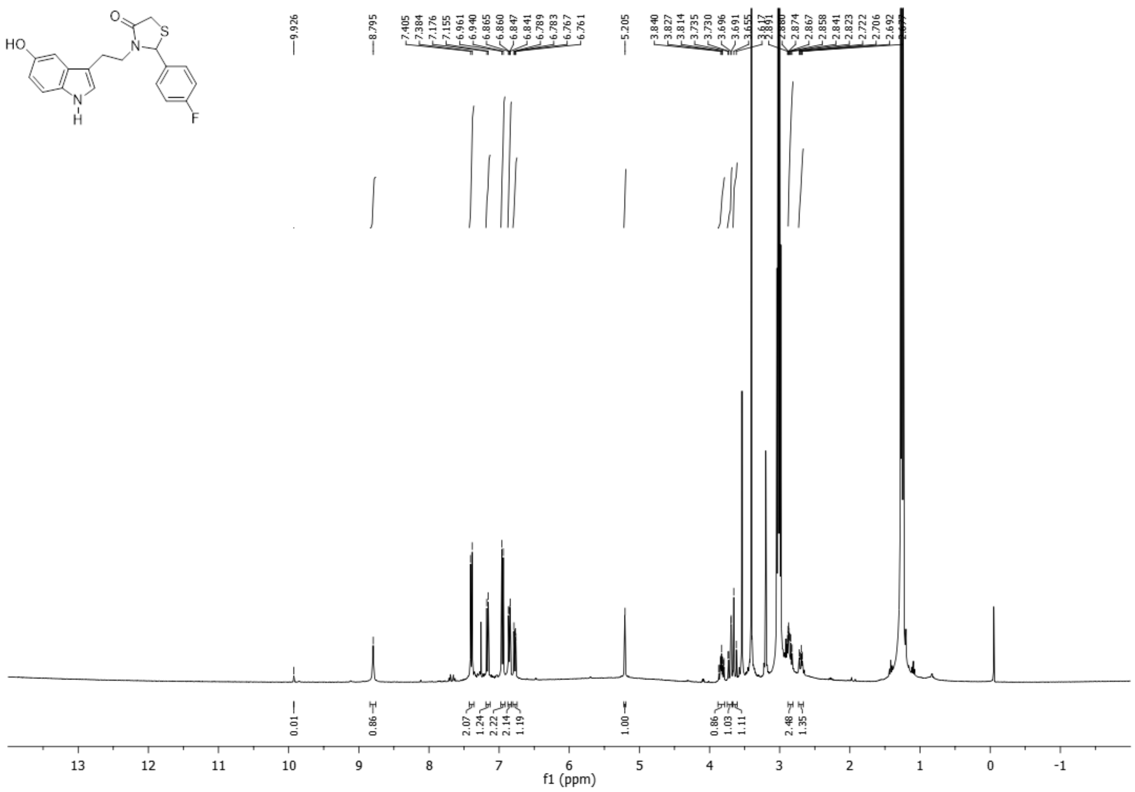

Figure S38: ^{1}H-NMR Spectrum of Compound **YS10**

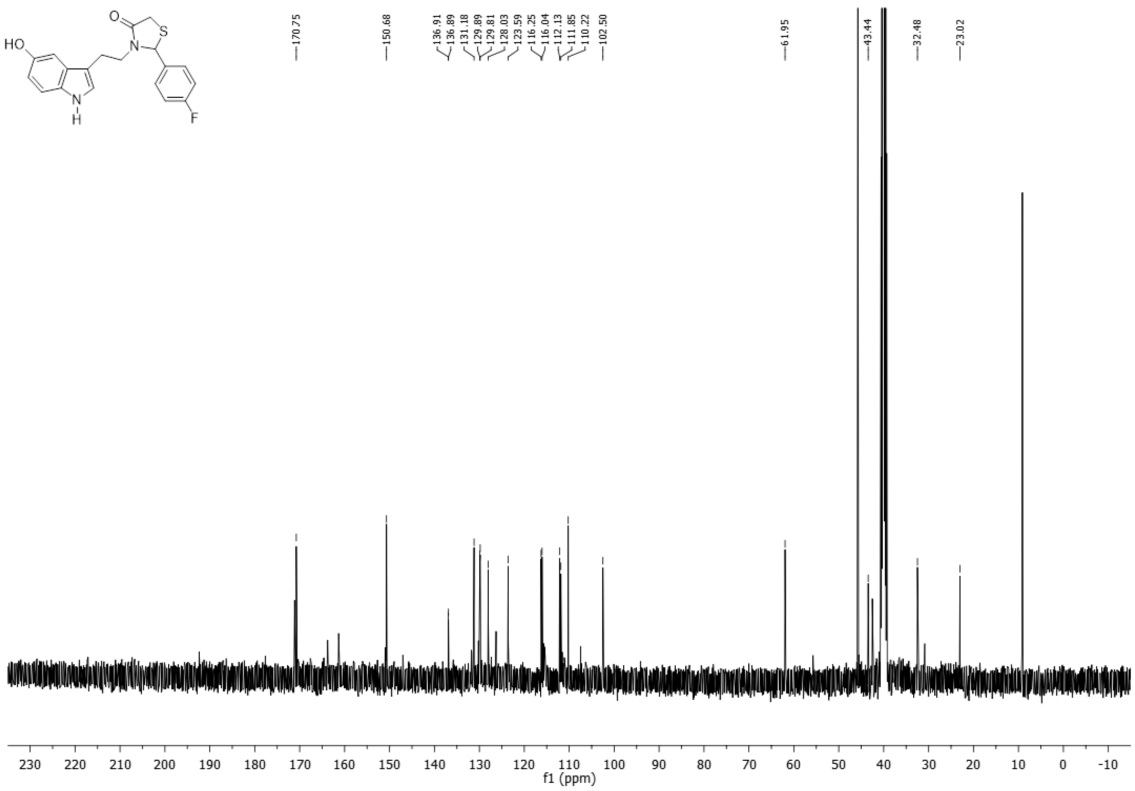

Figure S39: ^{13}C-NMR Spectrum of Compound **YS10**

Elemental Composition Report

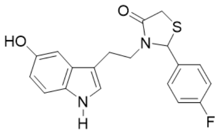

Single Mass Analysis

Tolerance = 10000.0 PPM / DBE: min = -5.5, max = 1000.0
Element prediction: Off
Number of isotope peaks used for i-FIT = 9

Monoisotopic Mass, Even Electron Ions
6 formula(e) evaluated with 1 results within limits (all results (up to 1000) for each mass)
Elements Used:
C: 19-19 H: 16-19 N: 2-5 O: 2-2 F: 1-2 S: 1-1
Seher Aydin
41102_20230926_10-N01 15 (0.586) Cm (14:25)

1: TOF MS ES-
1.10e+004

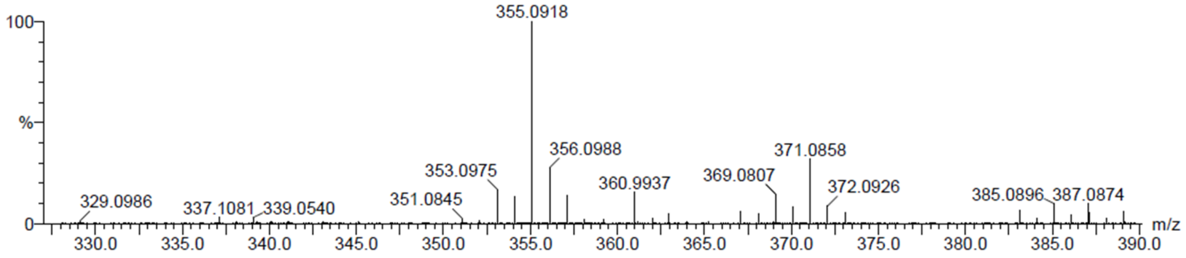

Minimum:				-5.5			
Maximum:		1000.0	10000.0	1000.0			
Mass	Calc. Mass	mDa	PPM	DBE	i-FIT	i-FIT (Norm)	Formula
355.0918	355.0917	0.1	0.3	12.5	773.9	0.0	C19 H16 N2 O2 F S

Figure S40: HRMS Spectrums of Compound **YS10**

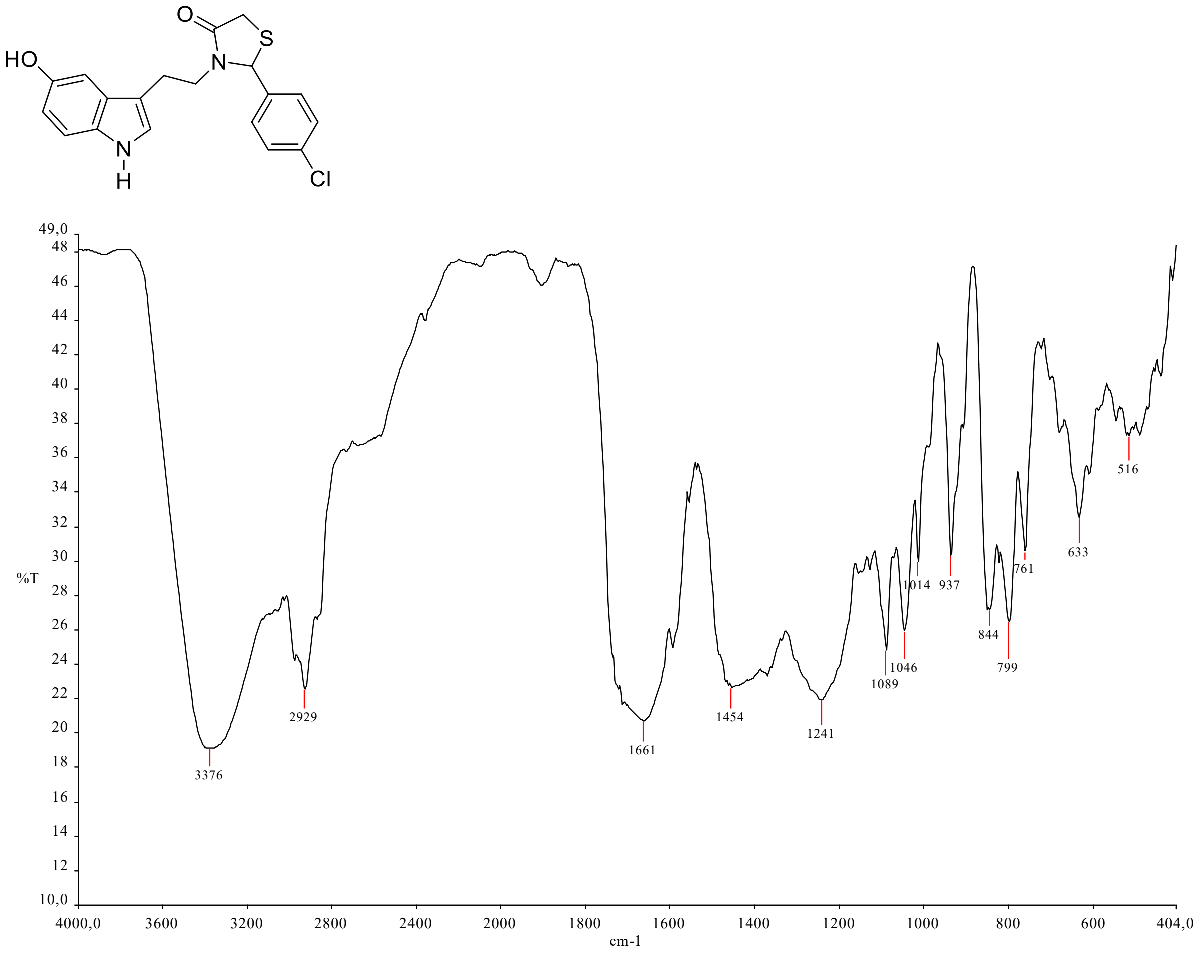

Figure S41: IR Spectrum of Compound **YS11**

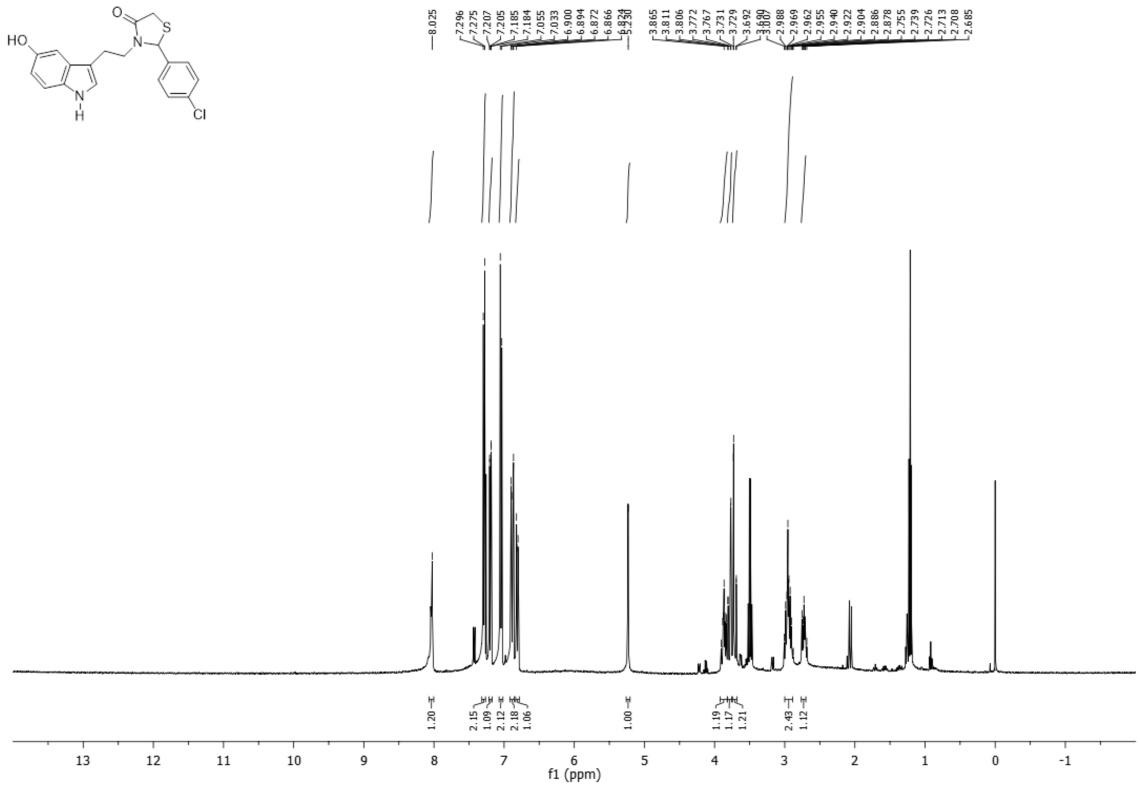

Figure S42: ^{1}H-NMR Spectrum of Compound **YS11**

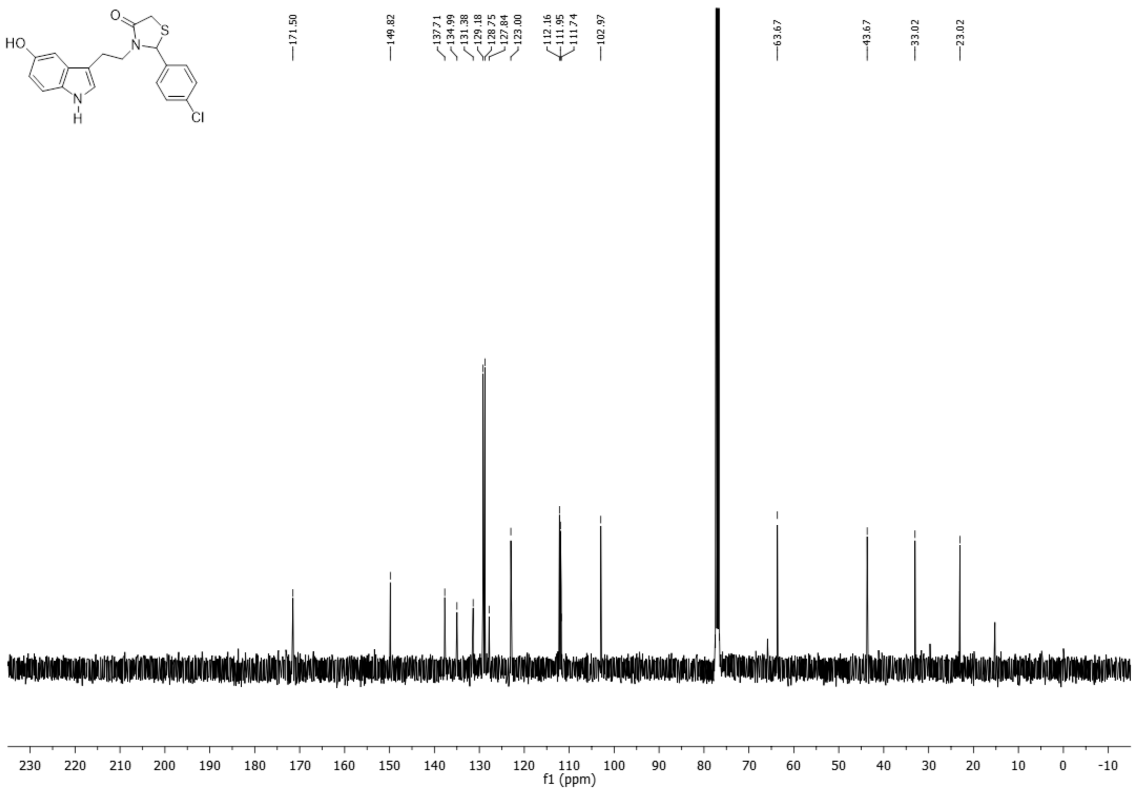

Figure S43: ^{13}C-NMR Spectrum of Compound **YS11**

Elemental Composition Report

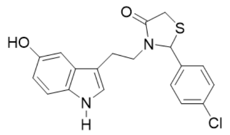

Single Mass Analysis

Tolerance = 10000.0 PPM / DBE: min = -5.5, max = 1000.0
Element prediction: Off
Number of isotope peaks used for i-FIT = 9

Monoisotopic Mass, Odd and Even Electron Ions
11 formula(e) evaluated with 1 results within limits (all results (up to 1000) for each mass)
Elements Used:
C: 19-19 H: 16-19 N: 2-5 O: 2-2 S: 1-1 Cl: 1-3
Seher Aydin
41102_20230926_11-01 4 (0.172) Cm (4:22)

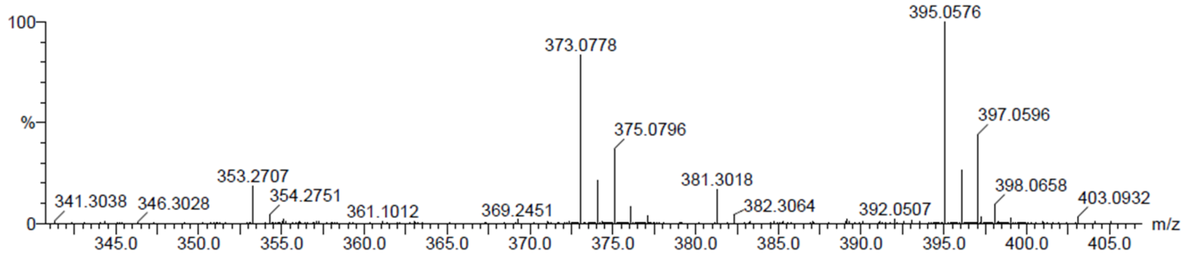

Minimum:				-5.5			
Maximum:		1000.0	10000.0	1000.0			
Mass	Calc. Mass	mDa	PPM	DBE	i-FIT	i-FIT (Norm)	Formula
373.0778	373.0778	0.0	0.0	11.5	1262.4	0.0	C19 H18 N2 O2 S Cl

Figure S44: HRMS Spectrums of Compound **YS11**

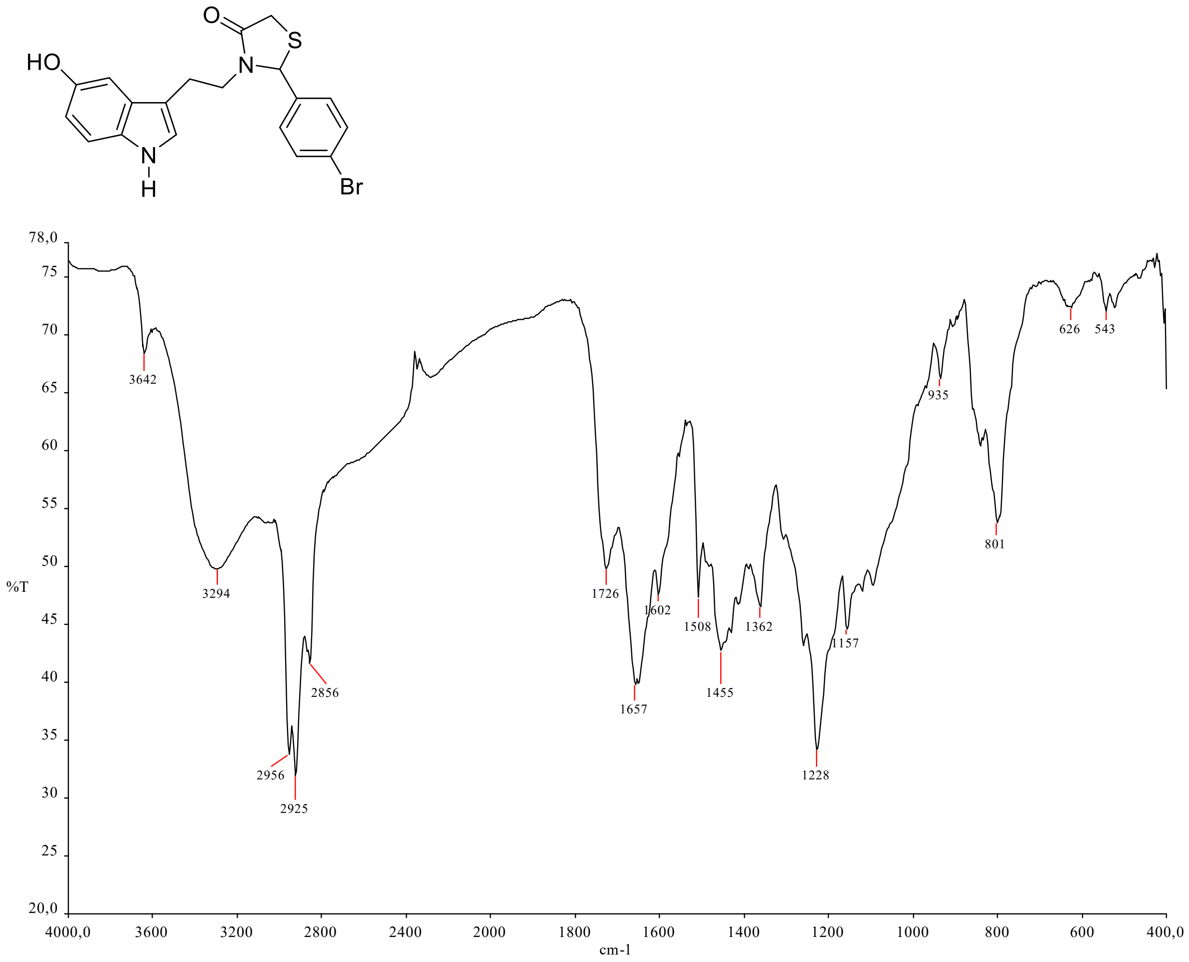

Figure S45: IR Spectrum of Compound **YS12**

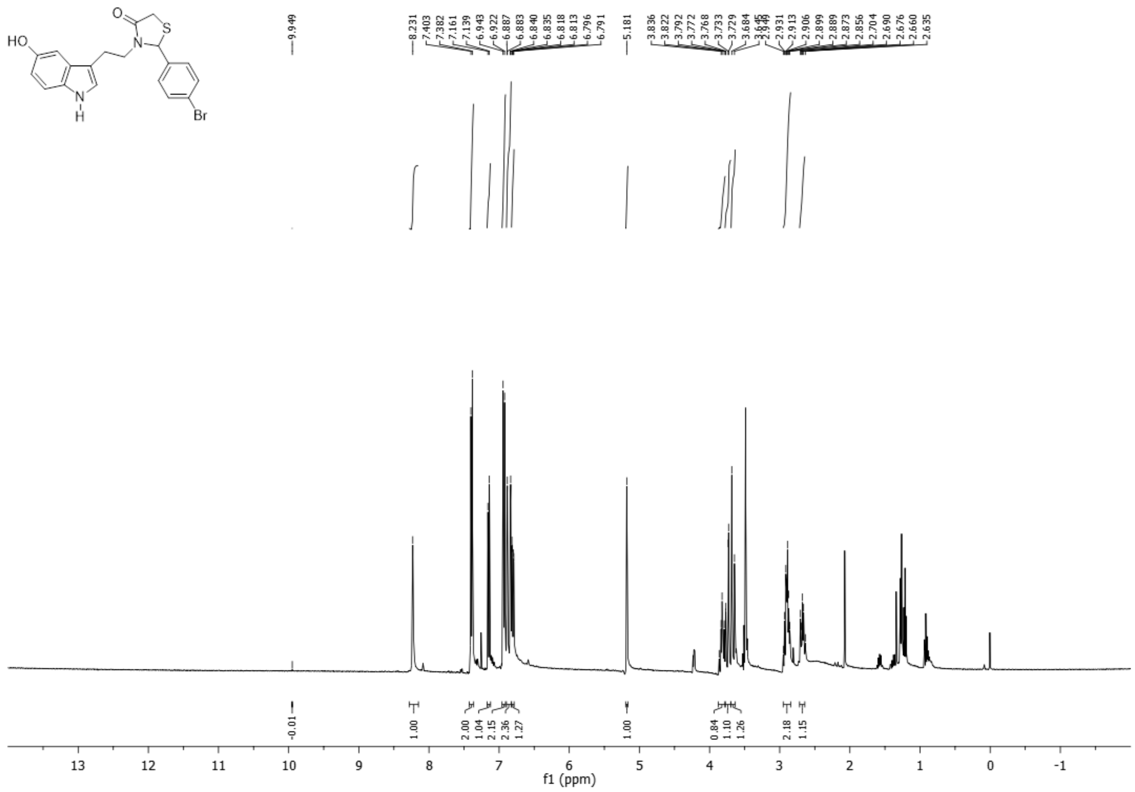

Figure S46: ^{1}H-NMR Spectrum of Compound **YS12**

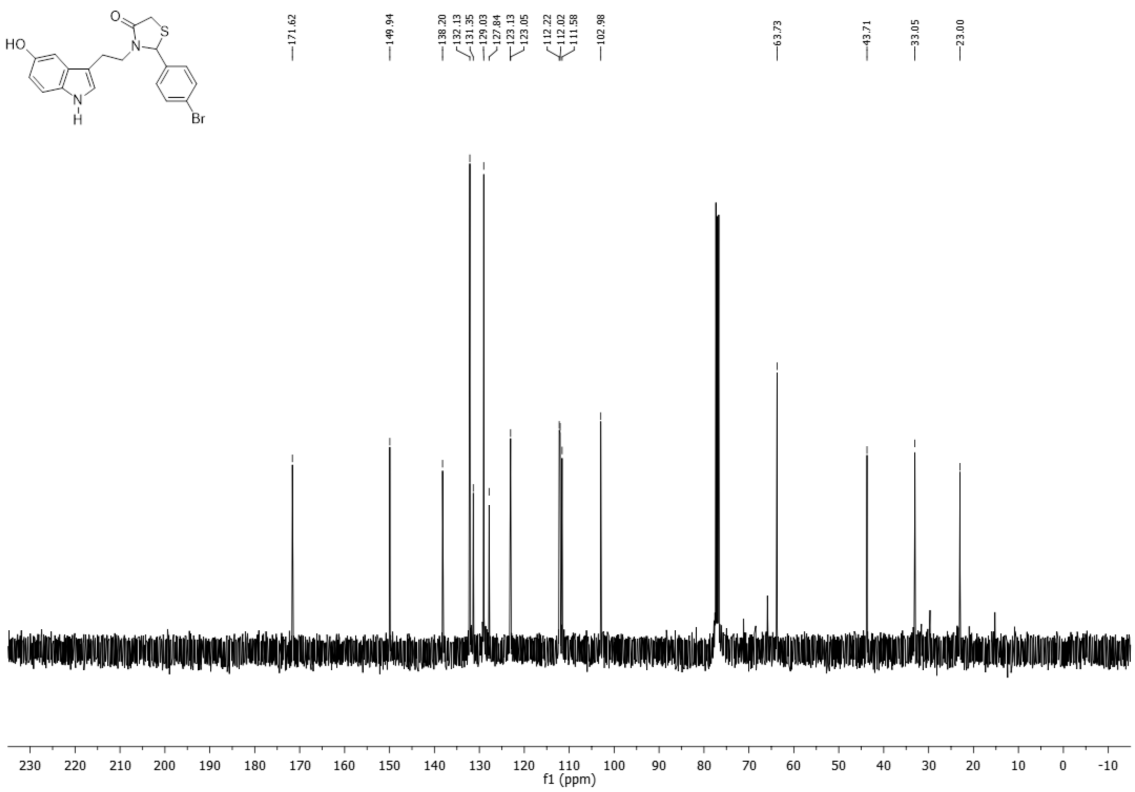

Figure S47: ^{13}C-NMR Spectrum of Compound **YS12**

Elemental Composition Report

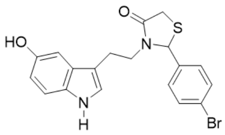

Single Mass Analysis
Tolerance = 10000.0 PPM / DBE: min = -5.5, max = 1000.0
Element prediction: Off
Number of isotope peaks used for i-FIT = 9

Monoisotopic Mass, Odd and Even Electron Ions
29 formula(e) evaluated with 1 results within limits (all results (up to 1000) for each mass)
Elements Used:
C: 19-19 H: 16-19 N: 2-5 O: 2-2 S: 1-1 79Br: 0-2 81Br: 0-2

Seher Aydin
41102_20230927_12-02 10 (0.396) Cm (2:11)

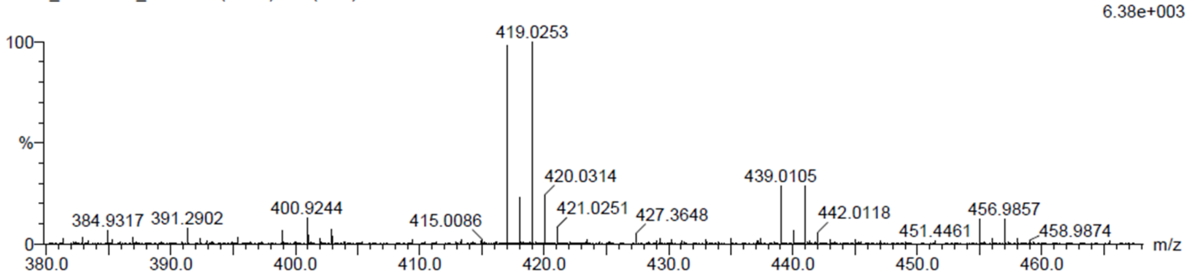

Minimum:				-5.5			
Maximum:		1000.0	10000.0	1000.0			
Mass	Calc. Mass	mDa	PPM	DBE	i-FIT	i-FIT (Norm)	Formula
419.0253	419.0252	0.1	0.2	11.5	705.6	0.0	C19 H18 N2 O2 S 81Br

Figure S48: HRMS Spectrums of Compound **YS12**

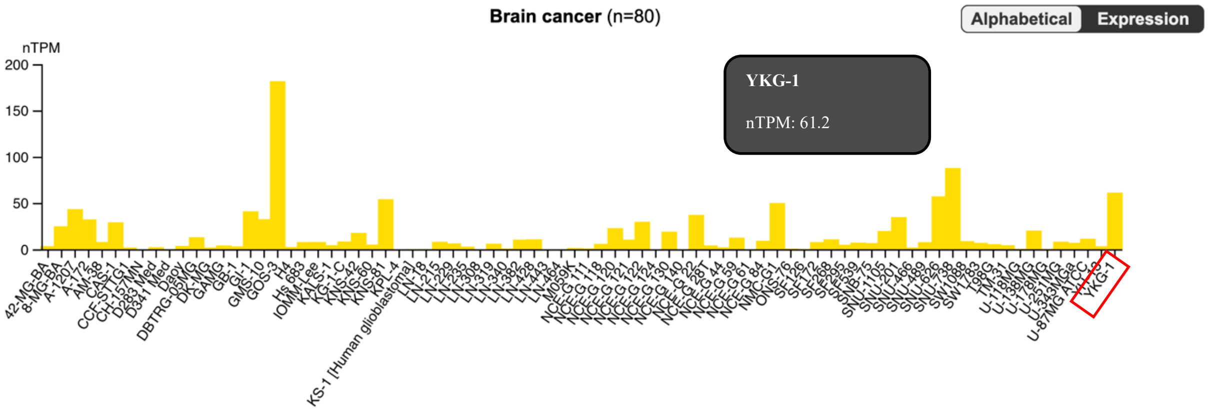

Figure S49: Overexpression of A1R1 in YKG-1 cell line.

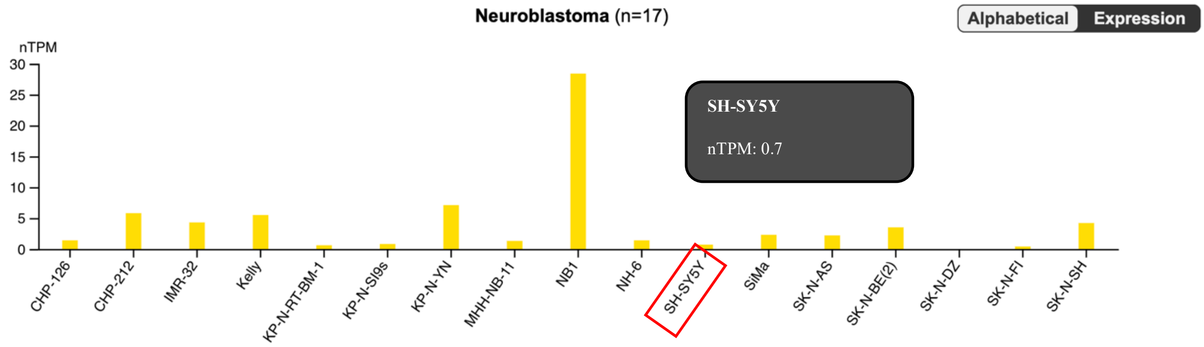

Figure S50: Overexpression of A1R1 in SH-SY5Y cell line.

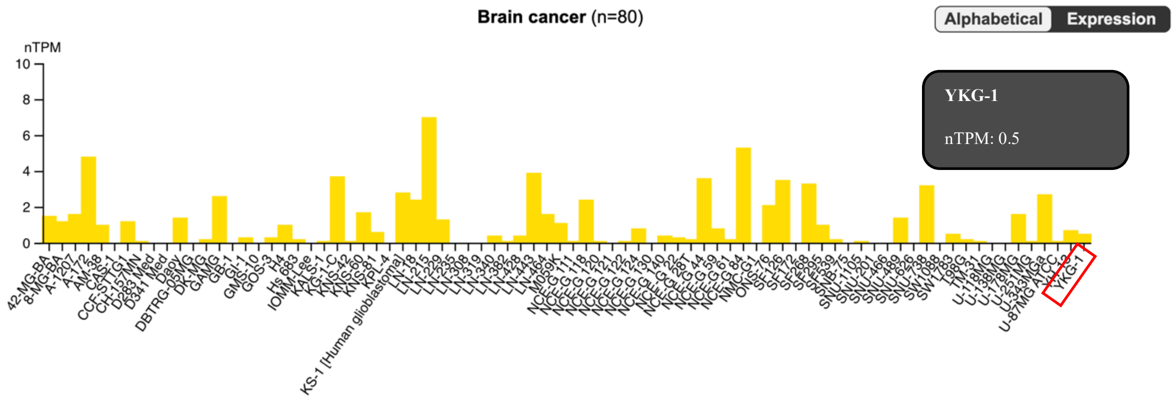

Figure S51: Overexpression of PDE10A in YKG-1 cell line.

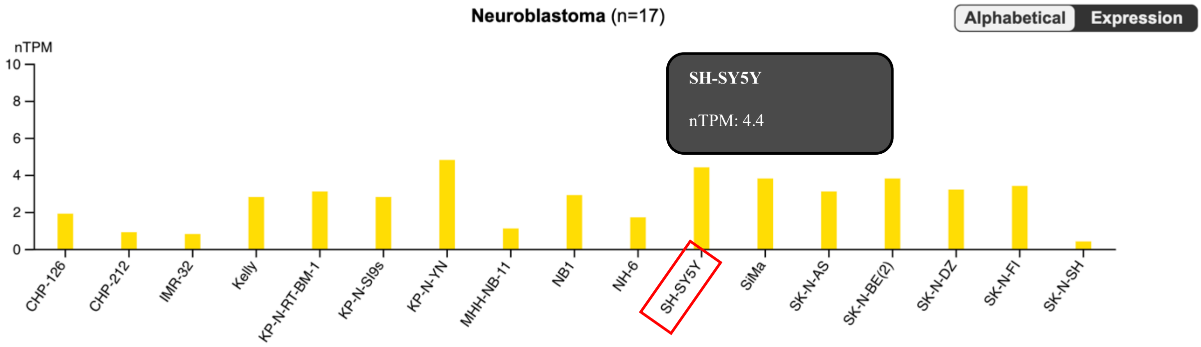

Figure S52: Overexpression of PDE10A in SH-SY5Y cell line.

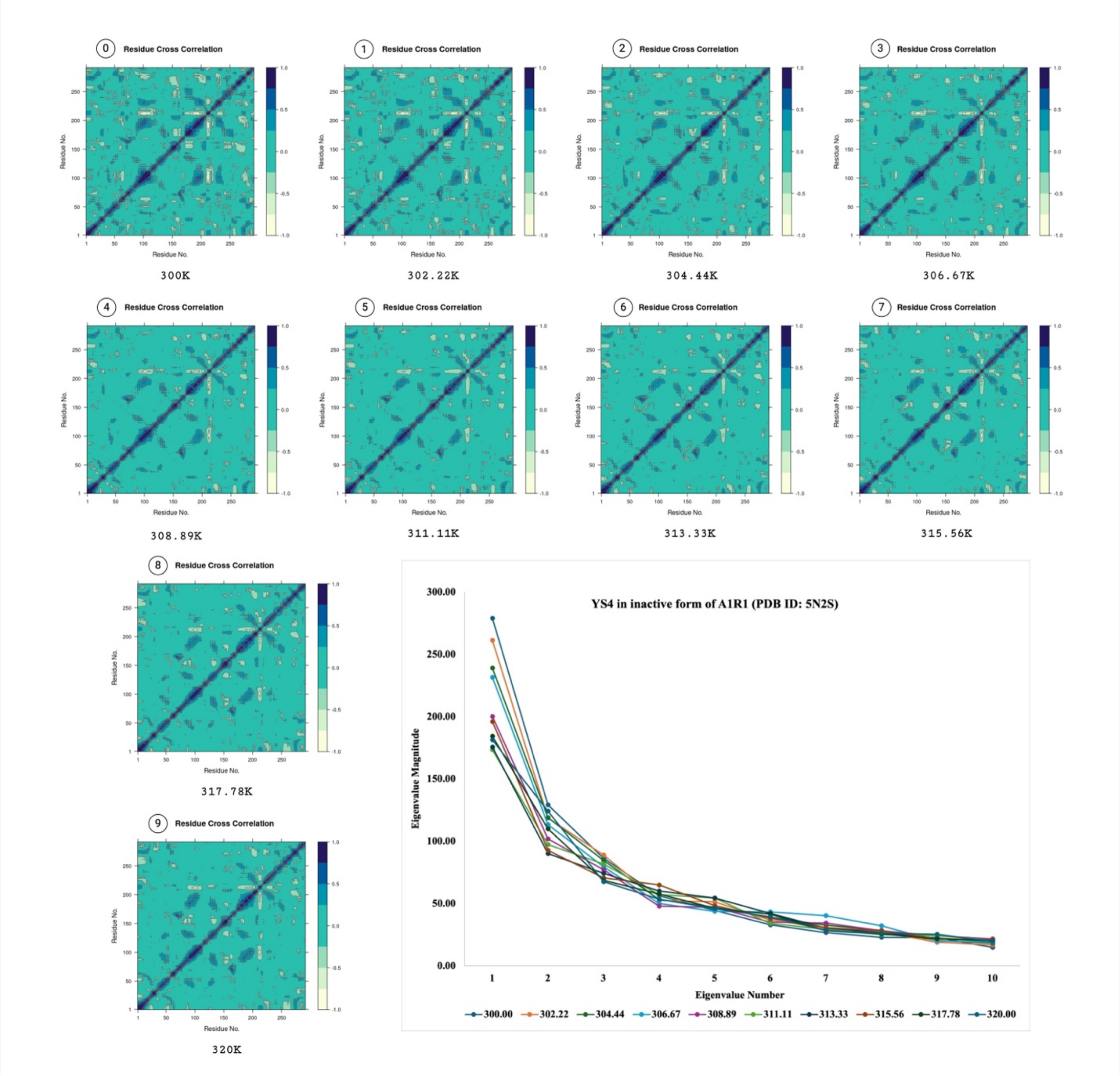

Figure S53: PCA showing the eigenvalue magnitudes of 10ns temperature-based replica exchange MD simulations (bottom-right) of **YS4** in the inactive form of A1R1 (PDB ID: 5N2S). 10 replicas have been run with a temperature range of 300-320K. Figure sections 0-9 show the residue cross correlation graphs between residues of the protein's first frame against the remaining simulation duration. Created with BioRender.com

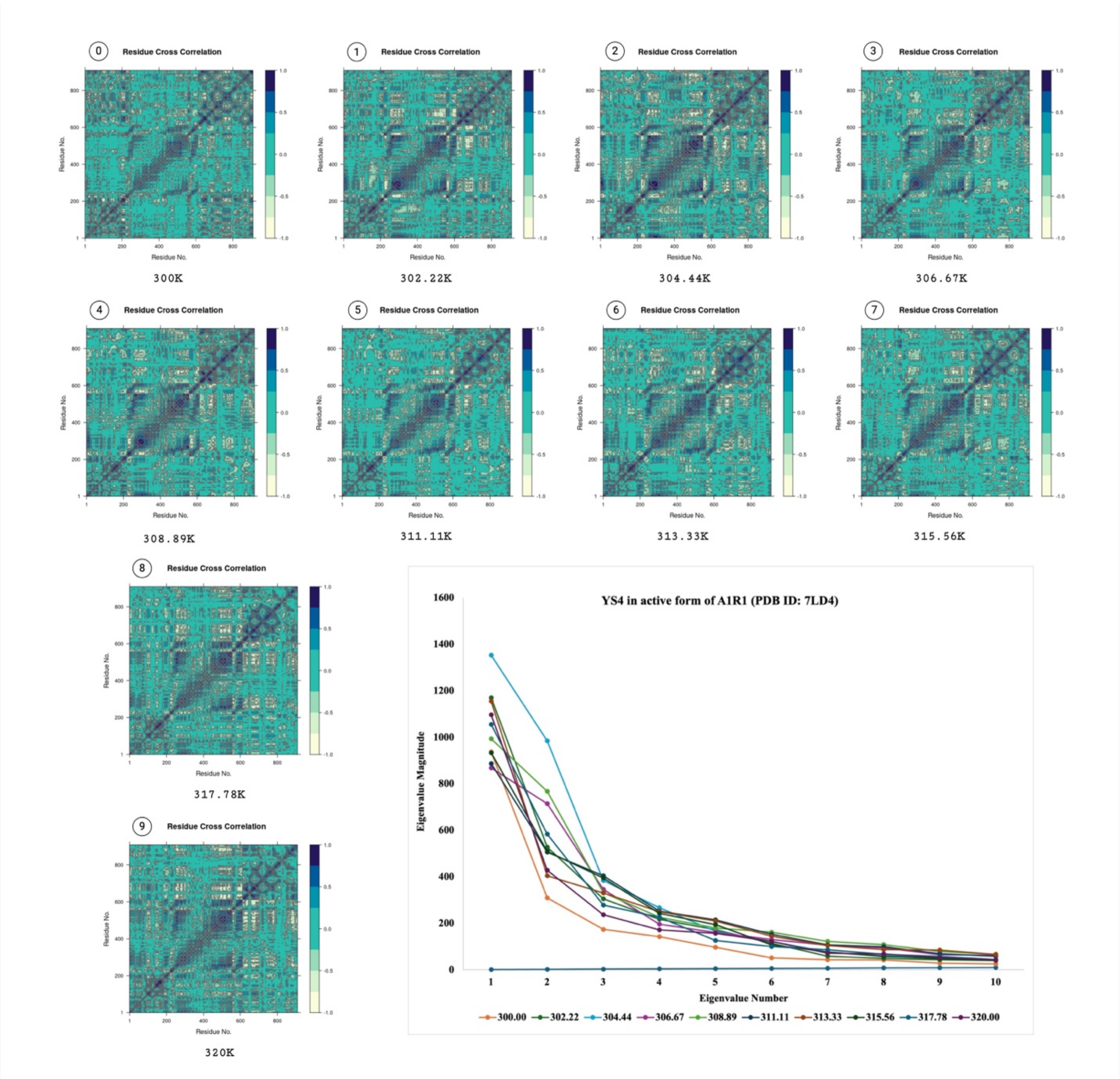

Figure S54: PCA showing the eigenvalue magnitudes of 10ns temperature-based replica exchange MD simulations (bottom-right) of **YS4** in the active form of A1R1 (PDB ID: 7LD4). 10 replicas have been run with a temperature range of 300-320K. Figure sections 0-9 show the residue cross correlation graphs between residues of the protein's first frame against the remaining simulation duration. Created with BioRender.com

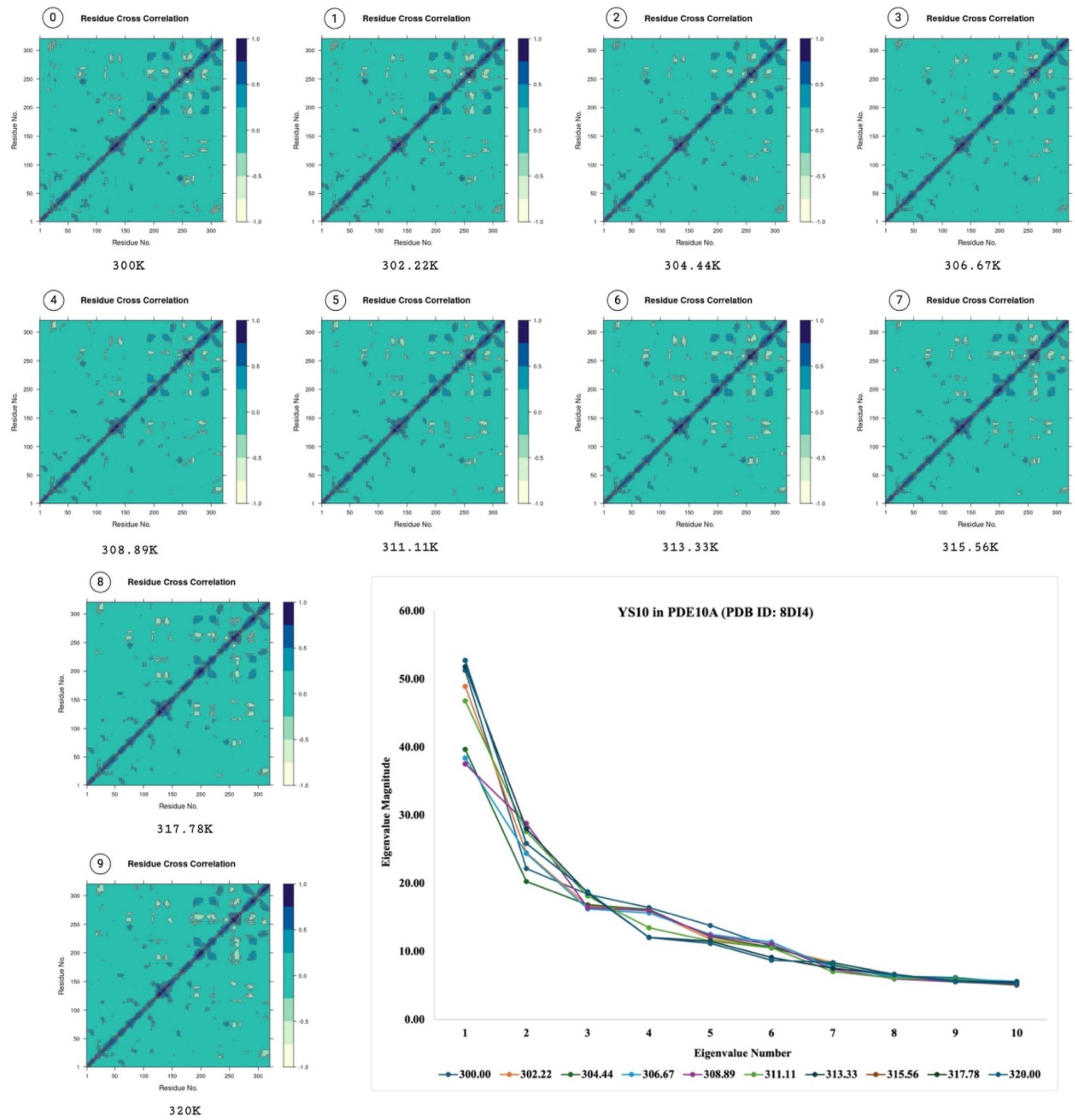

Figure S55: PCA showing the eigenvalue magnitudes of 10ns temperature-based replica exchange MD simulations (bottom-right) of **YS10** in PDE10A (PDB ID: 8DI4). 10 replicas have been run with a temperature range of 300-320K. Figure sections 0-9 show the residue cross correlation graphs between residues of the protein's first frame against the remaining simulation duration. Created with BioRender.com

Table S1: Binary QSAR Disease Model Parameters

Disease	Parameters
Depression	Evaluating potential activity against depression, the cutoff is set at 0.5. Values surpassing 0.5 suggest potentially active compounds. The training set comprises approved drugs, with a model description involving a training set of N=335, test set of N=62, sensitivity of 0.93, specificity of 0.82, accuracy of 0.87, and MCC of 0.75.
Bacterial	Assessing potential antibacterial activity, the cutoff is established at 0.5. Values exceeding 0.5 indicate potential activity. The training set includes approved drugs, with a model description featuring a training set of N=530, test set of N=97, sensitivity of 0.87, specificity of 0.90, accuracy of 0.89, and MCC of 0.77.
Cancer	Investigating potential activity against cancer, the cutoff is set at 0.5. Values surpassing 0.5 signify potential activity. The training set encompasses approved drugs, with a model description consisting of a training set of N=886, test set of N=167, sensitivity of 0.89, specificity of 0.83, accuracy of 0.86, and MCC of 0.72.
HIV	Exploring potential activity against HIV, the cutoff is fixed at 0.5. Values exceeding 0.5 indicate potential activity. The training set involves approved drugs, drug candidates in clinical trials, and preclinical compounds with in vivo activity. The model description includes a training set of N=491, test set of N=80, sensitivity of 0.80, specificity of 0.86, accuracy of 0.84, and MCC of 0.67.
Heart Failure	Examining potential activity against heart failure, the cutoff is set at 0.5. Values surpassing 0.5 suggest potential activity. The training set comprises approved drugs, with a model description featuring a training set of N=204, test set of N=33, sensitivity of 0.78, specificity of 0.87, accuracy of 0.82, and MCC of 0.64.
Hyperlipidemia	Assessing potential antihyperlipidemic activity, the cutoff is established at 0.5. Values exceeding 0.5 indicate potential activity. The training set includes approved drugs, with a model description consisting of a training set of N=185, test set of N=24, sensitivity of 0.75, specificity of 0.92, accuracy of 0.83, and MCC of 0.68.
Obesity	Exploring potential activity against obesity, the cutoff is fixed at 0.5. Values exceeding 0.5 indicate potential activity. The training set involves approved drugs, drug candidates in clinical trials, and preclinical compounds with in vivo activity. The model description includes a training set of N=472, test set of N=75, sensitivity of 0.89, specificity of 0.97, accuracy of 0.93, and MCC of 0.87.
Allergy	Investigating potential antiallergic activity, the cutoff is set at 0.5. Values surpassing 0.5 suggest potential activity. The training set comprises approved drugs, with a model description featuring a training set of N=258, test set of N=47, sensitivity of 0.87, specificity of 0.88, accuracy of 0.87, and MCC of 0.74.
Alzheimer	Evaluating potential activity against Alzheimer's disease, the cutoff is set at 0.5. Values exceeding 0.5 indicate potential activity. The training set encompasses approved drugs, drug candidates in clinical trials, and preclinical compounds with in vivo activity. The model description includes a training set of N=261, test set of N=44, sensitivity of 0.91, specificity of 0.82, accuracy of 0.86, and MCC of 0.73.
Parkinson	Examining potential activity against Parkinson's disease, the cutoff is set at 0.5. Values surpassing 0.5 suggest potential activity. The training set comprises approved drugs, drug candidates in clinical trials, and preclinical compounds with in vivo activity. The model description features a training set of N=298, test set of N=49, sensitivity of 0.96, specificity of 0.96, accuracy of 0.96, and MCC of 0.92.
Thrombosis	Assessing potential antithrombotic activity, the cutoff is established at 0.5. Values exceeding 0.5 indicate potential activity. The training set includes approved drugs, drug candidates in clinical trials, and preclinical compounds with in vivo activity. The model description consists of a training set of N=453, test set of N=80, sensitivity of 0.98, specificity of 0.95, accuracy of 0.97, and MCC of 0.93.
Diabetes	Exploring potential antidiabetic activity, the cutoff is fixed at 0.5. Values exceeding 0.5 indicate potential activity. The training set involves approved drugs, drug candidates in clinical trials, and preclinical compounds with in vivo activity. The model description features a training set of N=195, test set of N=34, sensitivity of 0.85, specificity of 0.93, accuracy of 0.88, and MCC of 0.77.

Psoriasis	Investigating potential activity against psoriasis, the cutoff is set at 0.5. Values surpassing 0.5 suggest potential activity. The training set comprises approved drugs, drug candidates in clinical trials, and preclinical compounds with in vivo activity. The model description includes a training set of N=199, test set of N=32, sensitivity of 0.93, specificity of 0.82, accuracy of 0.89, and MCC of 0.74.
Viral	Assessing potential antiviral activity, the cutoff is established at 0.5. Values exceeding 0.5 indicate potential activity. The training set includes approved drugs, with a model description consisting of a training set of N=206, test set of N=35, sensitivity of 0.92, specificity of 0.95, accuracy of 0.94, and MCC of 0.88.
Migraine	Examining potential activity against migraine, the cutoff is set at 0.5. Values surpassing 0.5 suggest potential activity. The training set comprises approved drugs, drug candidates in clinical trials, and preclinical compounds with in vivo activity. The model description features a training set of N=515, test set of N=98, sensitivity of 0.81, specificity of 0.84, accuracy of 0.83, and MCC of 0.65.
Hypertension	Evaluating potential antihypertensive activity, the cutoff is set at 0.5. Values exceeding 0.5 indicate potential activity. The training set includes approved drugs, with a model description involving a training set of N=554, test set of N=111, sensitivity of 0.89, specificity of 0.81, accuracy of 0.85, and MCC of 0.70.
Asthma	Exploring potential activity against asthma, the cutoff is fixed at 0.5. Values exceeding 0.5 indicate potential activity. The training set involves approved drugs, drug candidates in clinical trials, and preclinical compounds with in vivo activity. The model description includes a training set of N=366, test set of N=63, sensitivity of 0.92, specificity of 0.86, accuracy of 0.89, and MCC of 0.78.
Pain	Investigating potential analgesic activity, the cutoff is set at 0.5. Values surpassing 0.5 suggest potential activity. The training set comprises approved drugs, with a model description featuring a training set of N=525, test set of N=84, sensitivity of 0.92, specificity of 0.67, accuracy of 0.79, and MCC of 0.60.
Skin Diseases	Assessing potential activity against skin diseases, the cutoff is established at 0.5. Values exceeding 0.5 indicate potential activity. The training set includes approved drugs, with a model description consisting of a training set of N=255, test set of N=36, sensitivity of 1.00, specificity of 0.76, accuracy of 0.86, and MCC of 0.76.
Osteoporosis	Examining potential anti-osteoporosis activity, the cutoff is set at 0.5. Values surpassing 0.5 suggest potential activity. The training set comprises approved drugs, drug candidates in clinical trials, and preclinical compounds with in vivo activity. The model description features a training set of N=595, test set of N=86, sensitivity of 0.84, specificity of 0.85, accuracy of 0.85, and MCC of 0.70.
Inflammation	Investigating potential anti-inflammatory activity, the cutoff is set at 0.5. Values exceeding 0.5 indicate potential activity. The training set involves approved drugs, with a model description including a training set of N=598, test set of N=93, sensitivity of 0.86, specificity of 0.84, accuracy of 0.85, and MCC of 0.69.
Angina	Assessing potential antianginal activity, the cutoff is established at 0.5. Values exceeding 0.5 indicate potential activity. The training set includes approved drugs, drug candidates in clinical trials, and preclinical compounds with in vivo activity. The model description consists of a training set of N=546, test set of N=95, sensitivity of 0.90, specificity of 0.93, accuracy of 0.92, and MCC of 0.83.
Arthritis	Exploring potential activity against arthritis, the cutoff is fixed at 0.5. Values exceeding 0.5 indicate potential activity. The training set involves approved drugs, drug candidates in clinical trials, and preclinical compounds with in vivo activity. The model description features a training set of N=460, test set of N=77, sensitivity of 0.98, specificity of 0.94, accuracy of 0.96, and MCC of 0.92.
Mycosis	Investigating potential antifungal activity, the cutoff is set at 0.5. Values surpassing 0.5 suggest potential activity. The training set comprises approved drugs, with a model description including a training set of N=172, test set of N=47, sensitivity of 0.90, specificity of 0.88, accuracy of 0.89, and MCC of 0.79.
Schizophrenia	Assessing potential activity against schizophrenia, the cutoff is established at 0.5. Values exceeding 0.5 indicate potential activity. The training set includes approved drugs, drug candidates in clinical trials, and preclinical compounds with in vivo activity. The model description consists of a training set of N=616, test set of N=93, sensitivity of 0.89, specificity of 0.91, accuracy of 0.90, and MCC of 0.80.

Table S2: MetaDrug/Metacore Parameters of Physicochemical Properties

#	Property	Model Description
**1**	Formula	Represents the molecular formula.
**2**	HBA	Indicates the number of hydrogen bond acceptors.
**3**	HBD	Indicates the number of hydrogen bond donors.
**4**	MW	Represents the molecular weight.
**5**	RBN	Indicates the number of rotatable bonds.
**6**	Reactive	Identifies reactive groups within molecules. "OK" signifies that the metabolite lacks spontaneously reactive groups, while "R" indicates their presence. MetaDrug includes 89 rules to predict reactive metabolites such as quinones, aromatic and hydroxyl amines, acyl glucuronides, acyl halides, nepoxides, thiophene-S-oxides, furans, phenoxyl radicals, phenols, and aniline radicals.
**7**	RuleOf5	Refers to the Lipinski rule of five, which predicts the oral bioavailability of a molecule. A molecule should have no more than 5 hydrogen bond donors, no more than 10 hydrogen bond acceptors, a molecular weight below 500, and a partition coefficient (log P) under 5. Molecules compliant with these criteria are marked "OK," while non-compliant molecules are marked "Poor." Reference: Lipinski, et al., 2001 (PMID: 11259830).

Table S3: MetaDrug/MetaCore parameters of ADME QSAR Models

Property	Model Description
BBB, log ratio	The blood-brain barrier penetration model presents data as logarithmic values representing the ratio of metabolite concentrations in the brain to those in the plasma. A cutoff point of -0.3 is used, with higher values indicating a greater likelihood of metabolite penetration into the brain. Reference: Clarivate Analytics. Model specifications include a sample size (N) of 107, a coefficient of determination (R^2) of 0.89, and a root mean square error (RMSE) of 0.26.
G-LogP	Lipophilicity is represented as the logarithm of the compound's octanol-water distribution coefficient. The cutoffs range from -0.4 to 5.6, with compounds having values above 5.6 considered excessively hydrophobic. Reference: Syracuse Research, PHYSPROP database. Model specifications include a sample size (N) of 13,474, a coefficient of determination (R^2) of 0.95, and a root mean square error (RMSE) of 0.21.
Prot-bind, %	Human serum protein binding is expressed as a percentage. The cutoff point is set at 50%. Compounds with a binding percentage exceeding 95% are considered highly bound, while those below 50% are classified as low binding metabolites. Reference: Thummel and Shen, 2001, in Goodman & Gilman's The Pharmacological Basis of Therapeutics. Model specifications include a sample size (N) of 265, a coefficient of determination (R^2) of 0.909, and a root mean square error (RMSE) of 10.11.

Prot-bind, log t	Affinity to human serum albumin is represented by the logarithm of the retention time. A cutoff point of 0 is used, where positive values indicate higher protein binding, and negative values suggest lower protein binding. The acceptable level of binding varies depending on the project's requirements. The model is based on the retention times of compounds analyzed via HPLC using an immobilized HSA column, with retention times expressed as logarithmic values. Reference: Colmenarejo, Alvarez-Pedraglio, et al., 2001 (PMID: 11728183). Model specifications include a sample size (N) of 95, a coefficient of determination (R^2) of 0.904, and a root mean square error (RMSE) of 0.2.
WSol, log mg/L	Water solubility at 25 degrees Celsius is expressed as the logarithm of milligrams per liter (mg/L). The cutoffs range from 2 to 4, with the acceptable level of solubility varying based on the project's requirements. Reference: Syracuse Research, PHYSPROP database. Model specifications include a sample size (N) of 2,871, a coefficient of determination (R^2) of 0.91, and a root mean square error (RMSE) of 0.54.

Table S4: MetaDrug/MetaCore Parameters of Prediction of Toxic Effects

Property	Model Description
AMES	The potential for mutagenicity (AMES positive) is evaluated on a scale from 0 to 1. A score of 1 indicates AMES positivity (mutagenicity), while a score of 0 denotes AMES negativity (non-mutagenicity). The cutoff point is set at 0.5, with values closer to zero considered preferable. The AMES assay detects mutations in the histidine operon of Salmonella enterica sv Typhimurium. Reference: Young, Gombar, et al., 2002 (DOI: 10.1016/S0169-7439(01)00181-2). Model specifications include a sample size (N) of 1,780, a coefficient of determination (R^2) of 0.69, and a root mean square error (RMSE) of 0.29.
Anemia	The potential to cause anemia is evaluated using a cutoff point set at 0.5. Values above 0.5 indicate the potential presence of toxic compounds. The training set comprises chemicals and drugs known to induce anemia in vivo.<br><br>Model description: Training set size (N) = 324, Test set size (N) = 51, Sensitivity = 0.82, Specificity = 0.90, Accuracy = 0.86, and Matthews Correlation Coefficient (MCC) = 0.72. Reference: Clarivate Analytics.
Carcinogenicity	The potential to induce carcinogenicity in rats and mice is assessed with a cutoff threshold set at 0.5. Values above 0.5 indicate the potential presence of hazardous compounds. The training dataset includes chemicals and drugs known to cause carcinogenic effects in vivo, utilizing mice and rats as model organisms.<br><br>Reference: ISSCAN data. Model description: Training set size (N) = 1,210, Test set size (N) = 185, Sensitivity

	= 0.96, Specificity = 0.90, Accuracy = 0.93, and Matthews Correlation Coefficient (MCC) = 0.86.
Carcinogenicity Mouse Female	The potential to induce carcinogenicity specifically in female mice is evaluated with a cutoff set at 0.5. Values above 0.5 suggest the presence of potentially harmful compounds. The training dataset includes chemicals and drugs known to elicit carcinogenic effects in vivo, exclusively using female mice as model organisms.<br><br>Reference: ISSCAN data. Model description: Training set size (N) = 640, Test set size (N) = 94, Sensitivity = 0.90, Specificity = 0.93, Accuracy = 0.92, and Matthews Correlation Coefficient (MCC) = 0.83.
Carcinogenicity Mouse Male	The potential to induce carcinogenicity specifically in male mice is under evaluation with a cutoff set at 0.5. Values exceeding 0.5 indicate the potential presence of harmful compounds. The training dataset comprises chemicals and drugs known to cause carcinogenic effects in vivo, utilizing exclusively male mice as model organisms.<br><br>Reference: ISSCAN data. Model description: Training set size (N) = 584, Test set size (N) = 93, Sensitivity = 0.91, Specificity = 0.88, Accuracy = 0.89, Matthews Correlation Coefficient (MCC) = 0.78.
Carcinogenicity Rat Female	The potential to induce carcinogenicity specifically in female rats is under evaluation with a cutoff set at 0.5. Values exceeding 0.5 suggest the potential presence of harmful compounds. The training dataset includes chemicals and drugs known to cause carcinogenic effects in vivo, using exclusively female rats as model organisms.<br><br>Reference: ISSCAN data. Model description: Training set size (N) = 667, Test set size (N) = 120, Sensitivity = 0.90, Specificity = 0.96, Accuracy = 0.93, Matthews Correlation Coefficient (MCC) = 0.86.
Carcinogenicity Rat Male	The potential to induce carcinogenicity specifically in male rats is under evaluation, with a cutoff set at 0.5. Values exceeding 0.5 suggest the potential presence of harmful compounds. The training dataset includes chemicals and drugs known to cause carcinogenic effects in vivo, using exclusively male rats as model organisms.<br><br>Reference: ISSCAN data. Model description: Training set size (N) = 715, Test set size (N) = 117, Sensitivity = 0.92, Specificity = 0.88, Accuracy = 0.90, Matthews Correlation Coefficient (MCC) = 0.79.
Cardiotoxicity	The potential to induce cardiotoxicity is being assessed with a cutoff set at 0.5. Values exceeding 0.5 suggest the potential presence of toxic compounds. The training dataset comprises chemicals and drugs known to cause cardiotoxic effects in vivo across mouse, rat, and human models.<br><br>Model description: Training set size (N) = 143, Test set size (N) = 30, Sensitivity = 0.80, Specificity = 1.00, Accuracy = 0.90, Matthews Correlation Coefficient

	(MCC) = 0.82. Reference: Clarivate Analytics.
Cytotoxicity model, -log GI50 (M)	The growth inhibition of the MCF7 cell line (human Caucasian breast adenocarcinoma) is quantified using the pGI50 metric. A cutoff of 6 is utilized, where values ranging from 6 to 8 suggest the presence of a potentially toxic metabolite. Lower values are preferred, with values less than 6 considered more desirable, and values less than 3 indicating decreased toxicity.<br><br>Reference: DTP/NCI. Model description: Sample size (N) = 1,474, Coefficient of determination (R^2) = 0.9, Root Mean Square Error (RMSE) = 0.05.
Epididymis toxicity	The potential to induce epididymis toxicity is under evaluation with a cutoff set at 0.5. Values exceeding 0.5 indicate the potential presence of toxic compounds. The training dataset includes chemicals and drugs known to cause epididymis toxicity in vivo across mouse, rat, and human models.<br><br>Model description: Training set size (N) = 252, Test set size (N) = 42, Sensitivity = 0.90, Specificity = 0.86, Accuracy = 0.88, Matthews Correlation Coefficient (MCC) = 0.76. Reference: Clarivate Analytics.
Genotoxicity	The potential to induce epididymis toxicity is under evaluation with a cutoff set at 0.5. Values exceeding 0.5 indicate the potential presence of toxic compounds. The training dataset includes chemicals and drugs known to cause epididymis toxicity in vivo across mouse, rat, and human models.<br><br>Model description: Training set size (N) = 252, Test set size (N) = 42, Sensitivity = 0.90, Specificity = 0.86, Accuracy = 0.88, Matthews Correlation Coefficient (MCC) = 0.76. Reference: Clarivate Analytics.
Hepatotoxicity	The potential to induce hepatotoxicity is under evaluation with a cutoff set at 0.5. Values exceeding 0.5 suggest the potential presence of toxic compounds. The training dataset includes chemicals and drugs known to cause hepatotoxicity in vivo across mouse, rat, and human models.<br><br>Model description: Training set size (N) = 1,380, Test set size (N) = 231, Sensitivity = 0.73, Specificity = 0.88, Accuracy = 0.81, Matthews Correlation Coefficient (MCC) = 0.62. Reference: Clarivate Analytics.
Kidney Necrosis	The capacity to induce kidney necrosis is under assessment with a cutoff set at 0.5. Values surpassing 0.5 indicate the potential presence of toxic compounds. The training dataset includes chemicals and drugs known to induce renal necrosis in vivo across mouse, rat, and human models.<br><br>Model description: Training set size (N) = 221, Test set size (N) = 42, Sensitivity = 0.96, Specificity = 1.00, Accuracy = 0.98, Matthews Correlation Coefficient (MCC) = 0.95. Reference: Clarivate Analytics.
Kidney Weight Gain	The capacity to induce kidney weight gain is being assessed with a cutoff set at 0.5. Values exceeding 0.5

	suggest the potential presence of compounds capable of altering kidney weight. The training dataset includes chemicals and drugs known to cause kidney weight gain in vivo across mouse and rat models.<br><br>Model description: Training set size (N) = 240, Test set size (N) = 49, Sensitivity = 0.95, Specificity = 1.00, Accuracy = 0.98, Matthews Correlation Coefficient (MCC) = 0.96. Reference: Clarivate Analytics.
Liver Cholestasis	The potential to induce kidney weight gain is under evaluation with a cutoff set at 0.5. Values exceeding 0.5 suggest the presence of compounds capable of altering kidney weight. The training dataset comprises chemicals and drugs known to cause kidney weight gain in vivo across mouse and rat models.<br><br>Model details: Training set size (N) = 240, Test set size (N) = 49, Sensitivity = 0.95, Specificity = 1.00, Accuracy = 0.98, Matthews Correlation Coefficient (MCC) = 0.96. Reference: Clarivate Analytics.
Liver Lipid Accumulation	The potential to induce liver lipid accumulation is being assessed with a cutoff set at 0.5. Values exceeding 0.5 indicate the potential presence of compounds that may cause lipid accumulation. The training dataset includes chemicals and drugs known to induce lipid accumulation in vivo across mouse, rat, and human models.<br><br>Model details: Training set size (N) = 172, Test set size (N) = 28, Sensitivity = 0.80, Specificity = 0.85, Accuracy = 0.82, Matthews Correlation Coefficient (MCC) = 0.64. Reference: Clarivate Analytics.
Liver Necrosis	The capacity to induce liver necrosis is under assessment with a cutoff set at 0.5. Values exceeding 0.5 indicate the potential presence of compounds that could induce hepatic necrosis. The training dataset includes chemicals and drugs known to cause liver necrosis in vivo across mouse, rat, and human models.<br><br>Model details: Training set size (N) = 300, Test set size (N) = 57, Sensitivity = 0.91, Specificity = 0.91, Accuracy = 0.91, Matthews Correlation Coefficient (MCC) = 0.82. Reference: Clarivate Analytics.
Liver Weight Gain	The capacity to induce liver weight gain is being assessed with a cutoff set at 0.5. Values exceeding 0.5 suggest the potential presence of compounds capable of altering liver weight. The training dataset includes chemicals and drugs known to cause liver weight gain in vivo across mouse and rat models.<br><br>Model details: Training set size (N) = 292, Test set size (N) = 52, Sensitivity = 1.00, Specificity = 1.00, Accuracy = 1.00, Matthews Correlation Coefficient (MCC) = 1.00. Reference: Clarivate Analytics.
MRTD	The logarithmic Maximum Recommended Therapeutic Dose (log MRTD) is expressed in milligrams per kilogram body mass per day (mg/kg-bm/day), ranging from -5 to 3. A cutoff point of 0.5 is applied. Compounds with higher log MRTDs may be categorized as mildly toxic, whereas those with lower log MRTDs may be

	classified as highly toxic. Reference: Matthews, Kruhlak, et al., 2004 (PMID: 16472220). Model details: Sample size (N) = 1209, Coefficient of determination (R^2) = 0.86, Root Mean Square Error (RMSE) = 0.42.
Nasal pathology	The logarithmic Maximum Recommended Therapeutic Dose (log MRTD) is expressed in milligrams per kilogram body mass per day (mg/kg-bm/day), ranging from -5 to 3. A cutoff point of 0.5 is applied. Compounds with higher log MRTDs may be categorized as mildly toxic, whereas those with lower log MRTDs may be classified as highly toxic. Reference: Matthews, Kruhlak, et al., 2004 (PMID: 16472220). Model details: Sample size (N) = 1209, Coefficient of determination (R^2) = 0.86, Root Mean Square Error (RMSE) = 0.42.
Nephron Injury	The potential to induce nephron injury is under investigation, with a cutoff set at 0.5. Values exceeding 0.5 indicate the potential presence of toxic compounds. The training dataset includes chemicals and drugs known to cause nephron injury in vivo across mouse, rat, and human models. Model description: Training set size (N) = 598, Test set size (N) = 109, Sensitivity = 0.91, Specificity = 1.00, Accuracy = 0.96, Matthews Correlation Coefficient (MCC) = 0.93. Reference: Clarivate Analytics.
Nephrotoxicity	The potential to induce nephrotoxicity is under evaluation, with a cutoff set at 0.5. Values exceeding 0.5 indicate the potential presence of toxic compounds. The training dataset includes chemicals and drugs known to cause nephrotoxicity in vivo across mouse, rat, and human models. Model description: Training set size (N) = 847, Test set size (N) = 154, Sensitivity = 0.90, Specificity = 0.84, Accuracy = 0.87, Matthews Correlation Coefficient (MCC) = 0.74. Reference: Clarivate Analytics.
Neurotoxicity	The potential to induce neurotoxicity is under evaluation, with a cutoff set at 0.5. Values exceeding 0.5 suggest the potential presence of toxic compounds. The training dataset includes chemicals and drugs known to cause neurotoxicity in vivo across mouse, rat, and human models. Model description: Training set size (N) = 175, Test set size (N) = 34, Sensitivity = 0.94, Specificity = 0.94, Accuracy = 0.94, Matthews Correlation Coefficient (MCC) = 0.88. Reference: Clarivate Analytics.
Pulmonary toxicity	The potential to induce pulmonary toxicity is under assessment. The training dataset comprises chemicals and drugs known to elicit pulmonary toxicity in vivo across mouse, rat, and human models. A cutoff threshold of 0.5 is utilized, where values surpassing 0.5 suggest the presence of potentially toxic compounds. Model description: Training set size (N) = 482, Test set size (N) = 87, Sensitivity = 0.89, Specificity = 0.88, Accuracy = 0.89, Matthews Correlation Coefficient (MCC) = 0.77. Reference: Clarivate Analytics.
SkinSens, EC3	The skin sensitization potential is indicated by the effective concentration 3 (EC3), expressed as a percentage. Compounds with values greater than 10% are categorized as weak to moderate sensitizers.

	Reference: Ren et al. (PMID: 17723489). Model description: Sample size (N) = 89, Coefficient of determination (R^2) = 0.67, Root Mean Square Error (RMSE) = 22.56.
Testicular toxicity	The potential for inducing testicular toxicity is under evaluation, using a training dataset that includes chemicals and drugs known to cause testicular toxicity in vivo across mouse, rat, and human models. A cutoff value of 0.5 is applied, where values exceeding 0.5 indicate the presence of potentially toxic compounds. Model description: Training set size (N) = 439, Test set size (N) = 88, Sensitivity = 0.81, Specificity = 0.85, Accuracy = 0.83, Matthews Correlation Coefficient (MCC) = 0.66. Reference: Clarivate Analytics.
